# Inequalities in cancer screening participation between adults with and without severe mental illness: results from a cross-sectional analysis of primary care data on English Screening Programmes

**DOI:** 10.1038/s41416-023-02249-3

**Published:** 2023-05-04

**Authors:** Robert Stephen Kerrison, Alex Jones, Jianhe Peng, Gabriele Price, Julia Verne, Elizabeth Alexandra Barley, Cam Lugton

**Affiliations:** 1 School of Health Sciences, University of Surrey, Kate Granger Building, Surrey, Guildford, GU2 7XH UK; 2 Office for Health Improvement and Disparities, Department of Health and Social Care, 39 Victoria Street, London, SW1H 0EU UK

**Keywords:** Epidemiology, Population screening

## Abstract

**Background:**

People with severe mental illness (SMI) are 2.5 times more likely to die prematurely from cancer in England. Lower participation in screening may be a contributing factor.

**Methods:**

Clinical Practice Research Datalink data for 1.71 million, 1.34 million and 2.50 million adults were assessed (using multivariate logistic regression) for possible associations between SMI and participation in bowel, breast and cervical screening, respectively.

**Results:**

Screening participation was lower among adults with SMI, than without, for bowel (42.11% vs. 58.89%), breast (48.33% vs. 60.44%) and cervical screening (64.15% vs. 69.72%; all *p* < 0.001). Participation was lowest in those with schizophrenia (bowel, breast, cervical: 33.50%, 42.02%, 54.88%), then other psychoses (41.97%, 45.57%, 61.98%), then bipolar disorder (49.94%, 54.35%, 69.69%; all *p*-values < 0.001, except cervical screening in bipolar disorder; *p*-value > 0.05). Participation was lowest among people with SMI who live in the most deprived quintile of areas (bowel, breast, cervical: 36.17%, 40.23%, 61.47%), or are of a Black ethnicity (34.68%, 38.68%, 64.80%). Higher levels of deprivation and diversity, associated with SMI, did not explain the lower participation in screening.

**Conclusions:**

In England, participation in cancer screening is low among people with SMI. Support should be targeted to ethnically diverse and socioeconomically deprived areas, where SMI prevalence is greatest.

## Background

People with severe mental illness (SMI) are nearly two and half times more likely to die from cancer, under the age of 75, than those without SMI (cancer accounts for around 20% of premature deaths in adults with SMI) [1–9]. Several possible explanations for lower cancer survival, among those with SMI, have been suggested, including: delayed help-seeking, difficulties in communication and poorer adherence to treatment [10–13].

Although only contributing to earlier diagnosis and better outcomes in individuals with a diagnosis of bowel, breast or cervical cancer, it has been proposed that lower screening participation, by people with SMI, may partially explain their higher rates of premature mortality [14–16]. Recent research by Public Health England (PHE) supports this hypothesis, and reports that people with SMI are considerably less likely to participate in all three cancer screening programmes, with people with SMI being 18% less likely to participate in breast screening, 20% less likely to participate in cervical screening, and 31% less likely to participate in bowel screening (compared to people without SMI) [15].

While valuable, PHE’s analysis did not differentiate between SMI conditions (such as schizophrenia and bipolar disorder), and did not explore variation among those with SMI by sociodemographic characteristics [15]. As such, it is not known whether specific SMIs are associated with lower participation in individual cancer screening programmes, nor whether inequalities are exacerbated by known correlates of screening participation, such as age, sex and deprivation [17], some of which (e.g. Black or Minority Ethnic group, deprivation, smoking) are also associated with higher prevalence of SMI [18–20]. Greater understanding would enable better targeting of public health interventions and support for this vulnerable group, and may help to reduce the health inequalities experienced by them.

### Aim

The aims of this study were to: (1) verify whether inequalities in cancer screening participation, reported by PHE, persist after controlling for additional factors, which are known to be associated with screening participation, (2) test for possible associations between specific SMIs (such as schizophrenia and bipolar) and breast, bowel and cervical screening participation and (3) identify subgroups of individuals (with SMI) who are least likely to participate in screening (with a view of informing targeted strategies to promote participation within these groups).

## Methodology

### Study setting/context

The National Health Service in England offers free, universal screening for the early detection of breast, bowel and cervical cancers. Bowel cancer screening is offered once every two years (for men and women, aged between 60 and 74 years), breast cancer screening once every three years (for women, between the ages of 50 and 73) and cervical screening once every three and a half years (for women between the ages of 25 and 49), or once every five years (for women between the ages of 50 and 64), depending on age.

During the study period (defined below), guaiac faecal occult blood test (gFOBT) screening was the main bowel cancer screening method used (gFOBT was used up until June 2019, after which it was replaced with the faecal immunochemical test), mammography was offered for breast screening, and microscopic examination of cervical smears (i.e. ‘Pap smears’) were primarily offered for cervical screening (up until December 2019, when microscopic examination was replaced with primary Human Papilloma Virus [HPV] testing of cervical smears). gFOBT and FIT require the patient to collect and provide sample from one or more bowel motions, which can later be tested for blood. Cervical smears and primary HPV testing, meanwhile, involve the collection of cells, from the lining of the cervix, and is performed by a primary care nurse, using a speculum and swab. Mammography, finally, comprises an X-ray of the breasts, and is performed by a radiographer.

### Study design

To test the associations between SMI status (i.e. SMI, no SMI) and SMI condition (i.e. schizophrenia, other psychoses, bipolar disorder, no SMI) with cancer screening participation, we performed a cross-sectional analysis of adults who were registered with a general practice, in England, contributing to the Clinical Practice Research Datalink (CPRD).

### Study dataset

CPRD is a database comprised of medical records from patients registered at over 1000 General Practices in England. As of 2017, CPRD included 13% of England’s population, and was reported to be “broadly representative of the general population, with respect to deprivation, age and gender” [21]. The database is continuously updated and provides a reliable dataset to identify both cancer screening participation and SMI status (the latter is not available within routine national cancer screening programme databases). For the purposes of this study, data from 1987, to September 2020, were available to us. As individuals within the CPRD dataset are assigned a new patient ID each time they register with a new practice, individuals’ participation in the most recent screening round, only, was assessed (this was to avoid double counting and ensure the reliability and validity of the data). The actual time period of the analysis varied according to the type of screening being assessed (bowel, breast, or cervical), as each has a different interval length and eligibility criteria (see below).

For bowel screening, the analytic period was March 2017–September 2020 (the screening interval [two years], plus six months leeway to allow for rescheduling of appointments, reminders, etc.). For breast screening, the analytic period was March 2016–September 2020 (the screening interval [three yeas], plus 6 months leeway). Finally, for cervical screening, the analytic period was March 2016–September 2020 for women aged 25–49 (the screening interval [three and a half years], plus six months leeway), and March 2014–September 2020 for women aged 50–64 years (the screening interval [three and a half years], plus six months leeway). These are the groups who were eligible for the screening programmes as of September 2020, when data were first obtained. The dates were agreed by all members of the research team.

### Primary outcome measures

Participation in bowel, breast and cervical cancer screening programmes comprised the primary outcomes for this study. Participation was assessed using SNOMED (a structured clinical vocabulary for use in electronic health records) and Read codes (a clinical terminology system that was in widespread use in General Practice in England until 2018, when NHS England switched to using SNOMED codes). A patient was considered to have participated in a cancer screening programme if they were eligible for that programme (based on age and gender) and had a record of participating in that screening, within the recommended time period, running up to 1 September 2020, either from the Quality Outcomes Framework (version 45) expanded cluster list (SNOMED), the Primary Care Domain Reference Sets (SNOMED), or the NHS Code Browser (Read) (a list of the SNOMED and READ codes used in this research is provided in Appendix 1).

### Primary exposure

#### SMI status and condition

Each patient was assigned an SMI status and SMI group by the research team. SMI status was binary (SMI, no SMI), while SMI group was categorical, and comprised four groups; namely: schizophrenia, bipolar disorder, other psychoses (which included a range of psychotic conditions, some of which may be reflective of the individual’s stage within the diagnostic pathway [e.g. paranoid states], and some individuals being assigned to other SMI groups at a later stage [N.B. Non-SMI-related forms of psychosis, such as dementia, and drug and alcohol induced psychosis, were not included]) and no SMI (see Appendix 2 for an overview of the Read and SNOMED codes comprising the SMI groups). SMI was defined as bipolar disorder, schizophrenia and other psychotic disorders (excluding organic and substance induced psychotic disorders). The approach used to define SMI is relatively common and consistent with other work [22] and is similar to the Quality and Outcomes Framework (QOF) mental health register [23]. Sensitivity analysis indicated no impact on study outcomes for the cohort based on QOF or the amended SMI definition.

As SMI diagnosis can take some time from symptomatic presentation, no time restrictions were applied to SMI diagnosis. In other words, individuals with all diagnoses, which were not recorded as resolved, within the dataset, as of September 2020, were included in the analysis.

### Explanatory variables—demographics

To adjust for the effects of known correlates of screening participation, several demographic variables were extracted for inclusion in the analysis:

#### Age

Age was determined by subtracting the patient’s year of birth (available on the CPRD database) from the year at which data were extracted and analysed: 2020. To determine whether inequalities exist for specific age groups, each patient was then assigned an ‘age’ category’. Each age category comprised a five year period (e.g. 60–64), so as to ensure category had a sufficient number of patients available for statistical comparisons.

#### Sex

Sex was binary and extracted and coded as either male or female.

#### Ethnicity

Ethnicity was identified using SNOMED and READ codes, and categorised based on records in CPRD and Census groupings [24]: Asian (Indian, Pakistani, Bangladeshi, Chinese, Any other Asian background), Black (Caribbean, African, Any other Black, Black British or Caribbean background), Mixed (White and Black Caribbean, White and Black African, White and Asian, Any other Mixed or multiple ethnic background), White (English, Welsh, Scottish, Northern Irish or British, Irish, Gypsy or Irish Traveller, Roma, Any other White background), Other (Arab, Any other ethnic group) or not recorded (including prefer not to say/unknown). Where a patient had more than one record of observed ethnicity, the most recent observation was used.

#### Area-level deprivation

Area-level deprivation was derived from each person’s postcode, using the index of multiple deprivation (IMD): the government’s official measure of relative deprivation for small areas in England [25]. It uses administrative data and census-derived indicators of income, education, employment, living environment, health and disability, barriers to housing and services and crime at small-area level to allocate areas a deprivation score. CPRD sorts areas into quintiles of deprivation and assigns quintiles as 1 (least deprived) to 5 (most deprived). Where IMD based on patient’s postcode was not available, GP practice IMD was used.

#### Region

The address of each person’s GP was converted into one of (then) ten Strategic Health Authorities within England [26], namely: Northeast, Northwest, Yorkshire and Humber, East Midlands, West Midlands, East of England, South West, South Central, London and South East Coast.

### Explanatory variables—lifestyle risk factors

In addition to demographic factors associated with screening participation, several lifestyle risk factors, known to be associated with screening participation [27], and known to be more prevalent among people with SMI [28], were also extracted for inclusion in the analysis.

#### Smoking status

A patient’s current smoking status was identified with SNOMED concept ID codes within the Primary Care Domain reference set cluster ID ‘LSMOK_COD’/ PCD_Refset_ID ‘999004211000230104’. Individuals were coded as ‘Non-smokers’ or ‘smokers’.

#### Body mass index (BMI)

A patient’s BMI status was identified with SNOMED concept ID codes within the reference set cluster ID ‘BMI30_COD’/ PCD_Refset_ID ‘999011051000230106’. Individuals were coded as ‘BMI < 30’ and ‘BMI > 30’.

### Missing data

Missing data were coded as ‘unknown’ (there were missing data for ethnicity and area-level deprivation). Sensitivity analysis was carried out on imputed ‘unknown’ data and ethnicity coded as ‘White’, in line with previous research using primary care data that suggest that more than 93% of individuals without ethnicity recorded are from a white ethnic group [29]—there was no impact on study outcomes compared to coding data as ‘unknown’. In addition, patients who were not registered at their current GP at the start of the period under observation were removed from the population sample, before analysis was initiated. This was because it was not known if they participated in screening within the recommended time period, before joining their current GP. Previous analysis by PHE shows a similar proportion of SMI and non-SMI patients are excluded based on GP registration date [15] and, therefore, the impact on the study outcomes is likely to be negligible.

### Analysis

Descriptive statistics were used to report breast, bowel and cervical screening participation by SMI status, SMI group and demographic and lifestyle strata for SMI status (i.e. age, sex, ethnicity, area-level deprivation, region, smoking status and BMI). Univariate and multivariate logistic regression were then used to test for associations between SMI status and SMI group with bowel, breast and cervical screening participation (before and after adjusting for co-variates). Finally, univariate and multivariate logistic regression were used to test for subgroup differences in bowel, breast and cervical screening participation by SMI status. Analyses of subgroup differences in bowel, breast and cervical screening participation by SMI group were not conducted as the number of participants would be too small for conclusions to be made for individual conditions. The threshold for statistical significance was set at 0.05. All analyses were performed using R (Ver 4.0.2).

## Results

### Sample characteristics

In September 2020, the CPRD Aurum dataset included 13.1 million active patients in England, registered at 1309 different practices. 1.71 million were eligible for bowel screening, 1.34 million were eligible for breast screening and 2.50 million were eligible for cervical screening. One hundred and twenty-seven thousand one hundred and forty-two (127,142) adults had a recorded diagnosis of SMI, 10.31 million had a recorded ethnicity, 11.42 million had linked area-based deprivation data, 3.41 million were current smokers, and 421,161 had a BMI greater than 30 (sample characteristics vary according to the eligibility of individuals for each programme and are presented in Tables 2–4).

### Participation in bowel, breast and cervical screening by people with SMI

Overall, participation was lower among adults with SMI for bowel, breast and cervical screening (see Table 1). The largest differences in participation, between people with and without SMI, were observed for bowel cancer screening (42.11% vs. 58.89%; aOR: 0.57, 95% CI: 0.56–0.59; *p* < 0.001), followed by breast (48.33% vs. 60.44%; aOR: 0.68, 95% CI: 0.66–0.70; *p* < 0.001) and cervical (64.15% vs. 69.72%; aOR: 0.78, 95% CI: 0.76–0.80; *p* < 0.001, respectively; Table 1).

**Table 1:** Cancer screening participation by SMI status and SMI subgroup (unadjusted and adjusted models).

	*N* (%)	Proportion of eligible people who participated (95% CIs)	Unadjusted OR (95% CIs)	Adjusted OR (95% CIs)^a^
Bowel cancer screening (*n* = 1,711,429)				
No SMI	1,689,477 (98.72)	58.89 (58.82, 58.97)	1.00	1.00
SMI (all)	21,952 (1.28)	42.11 (41.45, 42.76)	0.51*** (0.49, 0.52)	
Bipolar	8052 (0.47)	49.94 (48.84, 51.04)	0.70*** (0.67, 0.73)	0.73*** (0.70, 0.77)
Other psychoses	6674 (0.39)	41.97 (40.78, 43.16)	0.50*** (0.48, 0.53)	0.57*** (0.54, 0.60)
Schizophrenia	7226 (0.42)	33.50 (32.42, 34.61)	0.35*** (0.33, 0.37)	0.43*** (0.41, 0.45)
Breast cancer screening (*n* = 1,347,238)				
No SMI	1,328,651 (98.62)	60.44 (60.36, 60.52)	1.00	1.00
SMI (all)	18,587 (1.38)	48.33 (47.61, 49.05)	0.61*** (0.59, 0.63)	
Bipolar	7776 (0.58)	54.35 (53.23, 55.46)	0.78*** (0.75, 0.81)	0.81*** (0.78, 0.85)
Other psychoses	6044 (0.45)	45.57 (44.31, 46.83)	0.55*** (0.52, 0.58)	0.62*** (0.59, 0.66)
Schizophrenia	4767 (0.35)	42.02 (40.61, 43.44)	0.47*** (0.45, 0.50)	0.58*** (0.54, 0.61)
Cervical Screening (*n* = 2,502,659)				
No SMI	2,473,557 (98.84)	69.72 (69.67, 69.78)	1.00	1.00
SMI (all)	29,102 (1.16)	64.15 (63.59, 64.70)	0.78*** (0.76, 0.80)	
Bipolar	13,611 (0.54)	69.69 (68.90, 70.46)	1.00 (0.96, 1.04)	0.97 (0.93, 1.00)
Other psychoses	9602 (0.38)	61.98 (61.00, 62.95)	0.71*** (0.68, 0.74)	0.73*** (0.70, 0.76)
Schizophrenia	5889 (0.24)	54.88 (53.60, 56.16)	0.53*** (0.50, 0.56)	0.55*** (0.52, 0.58)

*P < 0.05, **p  < 0.01, ***p < 0.001.

^a^Adjusted for age, sex (Bowel cancer screening, only), ethnicity, area-level deprivation, region, smoking status and BMI.

When broken down by SMI group, participation in all three programmes was lowest for people with schizophrenia, followed by people with other psychoses and bipolar disorder (see Table 1). When assessed using multivariate regression, participation for all three programmes was significantly lower for those with schizophrenia, compared to those with no SMI (Bowel: 33.50% vs. 58.89%; aOR: 0.43, 95% CI: 0.41–0.45; *p* < 0.001; Breast: 42.02% vs. 60.44%; aOR: 0.58, 95% CI: 0.54–0.61; *p* < 0.001; Cervical: 54.88% vs. 69.72%; aOR: 0.55, 95% CI: 0.52–0.58; *p* < 0.001; Table 1). The same was true for those with other psychoses, with participation in all three programmes being significantly lower, when compared with those without SMI (Bowel: 41.97% vs. 58.89%; aOR: 0.57, 95% CI: 0.54–0.60; *p* < 0.001; Breast: 45.57% vs. 60.44%; aOR: 0.62, 95% CI: 0.59–0.66; *p* < 0.001; Cervical: 61.98% vs. 69.72%; aOR: 0.73, 95% CI: 0.70–0.76; *p* < 0.001; Table 1). Results were slightly different for people with bipolar disorder, however. Indeed, while participation was significantly lower for these individuals, when compared to those without SMI, for bowel and breast cancer screening (Bowel: 49.94% vs. 58.89%; aOR: 0.73, 95% CI: 0.70–0.77; *p* < 0.001; Breast: 54.35% vs. 60.44%; aOR: 0.81, 95% CI: 0.78–0.85; *p* < 0.001), it was not significantly different, between populations, for cervical screening (69.69% vs. 69.72%; aOR: 0.97, 95% CI: 0.93, 1.00; *p* > 0.05; Table 1).

### Variation in participation in bowel, breast and cervical screening by people with SMI

Subgroup analyses revealed that participation in all three programmes was universally lower among people with SMI, compared to people without SMI (i.e. when stratified by age, sex, ethnicity, geographic region, area-level deprivation, BMI and smoking status; all *p*-values <0.05; see Tables 2–4). The only exceptions to this were participation in cervical screening, between women with and without SMI, aged 25–29 years, and participation in cervical screening between women, with and without SMI, from ‘Other’ ethnic groups (both *P*’s > 0.05; see Table 4).

**Table 2:** Variation in Bowel cancer screening participation, for adults with and without SMIs, by demographic characteristic, smoking status and BMI.

	Number of people		Proportion of eligible people who participated (95% CIs)		Unadjusted OR (95% CIs)	Adjusted OR (95%CIs)
	No SMI *N* (%)	SMI *N* (%)	No SMI % (95% CIs)	SMI % (95% CIs)		
Age^a^						
60–64	631,845 (37.40)	8866 (40.39)	48.70 (48.58, 48.82)	36.60 (35.60, 37.61)	0.61*** (0.58, 0.64)	0.68*** (0.65, 0.71)
65–69	528,181 (31.26)	6993 (31.86)	63.55 (63.42, 63.68)	44.84 (43.68, 46.02)	0.47*** (0.44, 0.49)	0.53*** (0.50, 0.55)
70–74	529,451 (31.34)	6093 (27.76)	66.42 (66.29, 66.55)	46.97 (45.71, 48.23)	0.45*** (0.43, 0.47)	0.50*** (0.47, 0.52)
Sex^b^						
Female	853,520 (50.52)	11,615 (52.91)	61.26 (61.15, 61.36)	44.74 (43.84, 45.65)	0.51*** (0.49, 0.53)	0.58*** (0.55, 0.60)
Male	835,957 (49.48)	10,337 (47.09)	56.48 (56.38, 56.59)	39.14 (38.20, 40.09)	0.50*** (0.48, 0.52)	0.57*** (0.55, 0.59)
Ethnicity^c^						
White	1,226,878 (72.62)	16,339 (74.43)	62.23 (62.15, 62.32)	44.01 (43.24, 44.77)	0.48*** (0.46, 0.49)	0.56*** (0.54, 0.58)
Asian	79,539 (4.71)	1351 (6.15)	48.78 (48.43, 49.12)	35.90 (33.35, 38.53)	0.59*** (0.53, 0.66)	0.60*** (0.54, 0.67)
Black	39,450 (2.34)	1093 (4.98)	47.73 (47.23, 48.22)	34.86 (32.05, 37.78)	0.59*** (0.52, 0.66)	0.60*** (0.53, 0.68)
Mixed	10,418 (0.62)	276 (1.26)	50.99 (50.02, 51.95)	39.49 (33.73, 45.55)	0.63*** (0.49, 0.80)	0.71** (0.56, 0.92)
Other	8767 (0.52)	134 (0.61)	49.05 (48.00, 50.10)	38.81 (30.63, 47.64)	0.66* (0.46, 0.93)	0.65* (0.46, 0.93)
Unknown	324,425 (19.20)	2759 (12.57)	50.63 (50.46, 50.80)	37.19 (35.39, 39.03)	0.58*** (0.53, 0.62)	0.60*** (0.55, 0.65)
Deprivation^d^						
Quintile 1 (least deprived)	398,985 (23.62)	2998 (13.66)	65.81 (65.67, 65.96)	50.27 (48.46, 52.07)	0.53*** (0.49, 0.56)	0.51*** (0.47, 0.55)
Quintile 2	360,624 (21.35)	3469 (15.80)	62.34 (62.18, 62.50)	47.82 (46.15, 49.50)	0.55*** (0.52, 0.59)	0.56*** (0.52, 0.60)
Quintile 3	321,234 (19.01)	3976 (18.11)	59.35 (59.18, 59.52)	44.29 (42.74, 45.85)	0.54*** (0.51, 0.58)	0.58*** (0.54, 0.62)
Quintile 4	301,819 (17.86)	4963 (22.61)	54.24 (54.07, 54.42)	38.75 (37.39, 40.12)	0.53*** (0.50, 0.57)	0.56*** (0.53, 0.59)
Quintile 5 (most deprived)	248,548 (14.71)	5773 (26.30)	47.86 (47.66, 48.06)	36.17 (34.93, 37.43)	0.62*** (0.58, 0.65)	0.64*** (0.60, 0.67)
Unknown	58,267 (3.45)	773 (3.52)	58.84 (58.44, 59.24)	39.46 (36.01, 43.01)	0.46*** (0.39, 0.53)	0.49*** (0.42, 0.57)
Region^e^						
North East	62,089 (3.68)	793 (3.61)	62.49 (62.11, 62.87)	46.15 (42.65, 49.70)	0.51*** (0.45, 0.59)	0.58*** (0.50, 0.67)
North West	312,572 (18.50)	4161 (18.95)	58.66 (58.49, 58.84)	41.05 (39.55, 42.56)	0.49*** (0.46, 0.52)	0.56*** (0.52, 0.60)
Yorkshire and Humber	65,446 (3.87)	806 (3.67)	63.38 (63.01, 63.74)	47.39 (43.91, 50.91)	0.52*** (0.45, 0.60)	0.58*** (0.50, 0.66)
East Midlands	35,822 (2.12)	340 (1.55)	62.60 (62.10, 63.10)	47.94 (42.54, 53.39)	0.55*** (0.44, 0.68)	0.59*** (0.48, 0.74)
West Midlands	292,386 (17.31)	3354 (15.28)	58.38 (58.20, 58.56)	41.83 (40.16, 43.52)	0.51*** (0.48, 0.55)	0.57*** (0.53, 0.62)
East of England	76,096 (4.50)	827 (3.77)	59.76 (59.41, 60.11)	46.92 (43.48, 50.38)	0.60*** (0.52, 0.68)	0.65*** (0.56, 0.75)
South West	221,017 (13.08)	2454 (11.18)	60.27 (60.06, 60.47)	43.19 (41.23, 45.18)	0.50*** (0.46, 0.54)	0.55*** (0.50, 0.59)
South Central	212,918 (12.60)	2378 (10.83)	62.10 (61.89, 62.31)	45.46 (43.45, 47.49)	0.51*** (0.47, 0.55)	0.56*** (0.52, 0.61)
London	249,518 (14.77)	4912 (22.38)	52.33 (52.14, 52.53)	37.66 (36.31, 39.04)	0.55*** (0.52, 0.58)	0.59*** (0.55, 0.62)
South East Coast	161,613 (9.57)	1927 (8.78)	59.88 (59.64, 60.12)	43.69 (41.47, 45.95)	0.52*** (0.47, 0.57)	0.55*** (0.51, 0.61)
Smoking status^f^						
Non-smoker	1,094,308 (64.77)	10,478 (47.73)	62.47 (62.38, 62.56)	47.00 (46.04, 47.96)	0.53*** (0.51, 0.55)	0.55*** (0.53, 0.58)
Smoker	595,169 (35.23)	11,474 (52.27)	52.33 (52.20, 52.45)	37.63 (36.75, 38.53)	0.55*** (0.53, 0.57)	0.59*** (0.57, 0.61)
BMI^g^						
BMI ≤ 30	1,574,918 (93.22)	19,469 (88.69)	59.04 (58.96, 59.12)	41.83 (41.14, 42.53)	0.50*** (0.48, 0.51)	0.56*** (0.54, 0.58)
BMI > 30	114,559 (6.78)	2483 (11.31)	56.90 (56.61, 57.19)	44.26 (42.30, 46.24)	0.60*** (0.56, 0.65)	0.67*** (0.62, 0.73)

*P < 0.05, **p < 0.01, ***p < 0.001.

^a^Adjusted for sex, ethnicity, region, area-level deprivation, smoking status and BMI.

^b^Adjusted for age, ethnicity, region, area-level deprivation, smoking status and BMI.

^c^Adjusted for age, sex, region, area-level deprivation, smoking status and BMI.

^d^Adjusted for age, sex, ethnicity, region, and smoking status and BMI.

^e^Adjusted for age, sex, ethnicity, area-level deprivation, smoking status and BMI.

^f^Adjusted for age, sex, ethnicity, region, area-level deprivation and BMI.

^g^Adjusted for age, sex, region, ethnicity, area-level deprivation and smoking status.

**Table 3:** Variation in breast cancer screening participation, for adults with and without SMIs, by demographic characteristic, smoking status and BMI.

	Number of people		Proportion of eligible people who participated (95% CIs)		Unadjusted OR (95% CIs)	Adjusted OR (95% CIs)
	No SMI *N* (%)	SMI *N* (%)	No SMI	SMI		
Age^a^						
50–54	365,609 (27.52)	4929 (26.52)	51.03 (50.86, 51.19)	40.88 (39.51, 42.27)	0.66*** (0.63, 0.70)	0.74*** (0.70, 0.78)
55–59	355,596 (26.76)	5218 (28.07)	63.22 (63.06, 63.38)	50.86 (49.50, 52.23)	0.60*** (0.57, 0.64)	0.69*** (0.65, 0.73)
60–64	299,753 (22.56)	4287 (23.06)	64.22 (64.05, 64.39)	50.52 (49.02, 52.03)	0.57*** (0.54, 0.60)	0.64*** (0.61, 0.69)
65–69	257,554 (19.38)	3484 (18.74)	64.74 (64.56, 64.93)	51.41 (49.73, 53.08)	0.58*** (0.54, 0.62)	0.64*** (0.60, 0.69)
70–74	50,139 (3.77)	669 (3.60)	64.69 (64.27, 65.10)	53.36 (49.50, 57.19)	0.62*** (0.54, 0.73)	0.70*** (0.60, 0.82)
Ethnicity^b^						
White	941,953 (70.90)	13,339 (71.77)	62.98 (62.88, 63.08)	50.53 (49.68, 51.38)	0.60*** (0.58, 0.62)	0.67*** (0.65, 0.69)
Asian	73,041 (5.50)	1248 (6.71)	49.73 (49.36, 50.09)	40.95 (38.21, 43.74)	0.70*** (0.63, 0.79)	0.72*** (0.64, 0.81)
Black	48,665 (3.66)	1334 (7.18)	44.95 (44.50, 45.39)	38.68 (36.07, 41.36)	0.77*** (0.69, 0.86)	0.78*** (0.70, 0.88)
Mixed	12,970 (0.98)	333 (1.79)	51.03 (50.17, 51.90)	38.74 (33.52, 44.22)	0.61*** (0.49, 0.76)	0.68** (0.54, 0.86)
Other	8688 (0.65)	135 (0.73)	46.41 (45.36, 47.46)	43.70 (35.27, 52.50)	0.90 (0.64, 1.26)	0.87 (0.61, 1.23)
Unknown	243,334 (18.31)	2198 (11.83)	57.91 (57.72, 58.11)	46.77 (44.67, 48.88)	0.64*** (0.59, 0.69)	0.67*** (0.61, 0.73)
Deprivation^c^						
Quintile 1 (least deprived)	306,516 (23.07)	2489 (13.39)	67.54 (67.37, 67.70)	59.46 (57.50, 61.39)	0.70*** (0.65, 0.76)	0.69*** (0.64, 0.75)
Quintile 2	273,336 (20.57)	2782 (14.97)	65.16 (64.98, 65.34)	56.11 (54.24, 57.96)	0.68*** (0.63, 0.74)	0.70*** (0.64, 0.75)
Quintile 3	248,205 (18.68)	3237 (17.42)	61.13 (60.94, 61.32)	50.91 (49.17, 52.65)	0.66*** (0.62, 0.71)	0.68*** (0.63, 0.73)
Quintile 4	244,503 (18.40)	4363 (23.47)	55.26 (55.06, 55.45)	44.65 (43.17, 46.14)	0.65*** (0.62, 0.69)	0.68*** (0.63, 0.72)
Quintile 5 (most deprived)	211,370 (15.91)	5088 (27.37)	50.36 (50.15, 50.57)	40.23 (38.88, 41.60)	0.66*** (0.63, 0.70)	0.67*** (0.64, 0.71)
Unknown	44,721 (3.37)	628 (3.38)	55.05 (54.59, 55.51)	47.61 (43.65, 51.60)	0.74*** (0.63, 0.87)	0.79** (0.67, 0.93)
Region^d^						
North East	47,272 (3.56)	630 (3.39)	65.59 (65.16, 66.02)	53.02 (49.03, 56.96)	0.59*** (0.51, 0.69)	0.60*** (0.51, 0.71)
North West	242,187 (18.23)	3689 (19.85)	63.02 (62.82, 63.21)	49.74 (48.12, 51.37)	0.58*** (0.54, 0.62)	0.66*** (0.62, 0.7)
Yorkshire and Humber	48,287 (3.63)	668 (3.59)	70.62 (70.21, 71.02)	58.98 (55.14, 62.72)	0.60*** (0.51, 0.70)	0.68*** (0.58, 0.8)
East Midlands	27,587 (2.08)	281 (1.51)	68.44 (67.89, 68.99)	54.45 (48.43, 60.35)	0.55*** (0.44, 0.70)	0.66** (0.51, 0.85)
West Midlands	22,1641 (16.68)	2698 (14.52)	54.45 (54.24, 54.66)	45.63 (43.74, 47.53)	0.70*** (0.65, 0.76)	0.75*** (0.70, 0.81)
East of England	61,391 (4.62)	700 (3.77)	69.45 (69.08, 69.81)	60.29 (56.54, 63.91)	0.67*** (0.57, 0.78)	0.72*** (0.61, 0.84)
South West	165,136 (12.43)	1926 (10.36)	65.17 (64.94, 65.40)	51.40 (49.14, 53.65)	0.57*** (0.52, 0.62)	0.61*** (0.56, 0.67)
South Central	169,436 (12.75)	2019 (10.86)	65.52 (65.30, 65.75)	55.32 (53.12, 57.51)	0.65*** (0.60, 0.71)	0.67*** (0.61, 0.73)
London	222,894 (16.78)	4439 (23.88)	49.06 (48.85, 49.27)	38.50 (37.07, 39.95)	0.65*** (0.61, 0.69)	0.68*** (0.64, 0.72)
South East Coast	122,820 (9.24)	1537 (8.27)	61.16 (60.88, 61.43)	51.92 (49.39, 54.44)	0.69*** (0.62, 0.76)	0.77*** (0.70, 0.86)
Smoking status^e^						
Non-smoker	858,462 (64.61)	8696 (46.79)	62.22 (62.12, 62.33)	51.91 (50.85, 52.96)	0.66*** (0.63, 0.68)	0.71*** (0.68, 0.74)
Smoker	470,189 (35.39)	9891 (53.21)	57.18 (57.04, 57.32)	45.18 (44.20, 46.17)	0.62*** (0.59, 0.64)	0.66*** (0.63, 0.69)
BMI^f^						
BMI < 30	1,237,757 (93.16)	16,046 (86.33)	60.47 (60.38, 60.56)	48.16 (47.38, 48.93)	0.61*** (0.59, 0.63)	0.68*** (0.65, 0.70)
BMI > 30	90,894 (6.84)	2541 (13.67)	60.02 (59.70, 60.34)	49.43 (47.47, 51.39)	0.65*** (0.60, 0.70)	0.72*** (0.67, 0.78)

*P < 0.05, **p < 0.01, ***p < 0.001.

^a^Adjusted for ethnicity, region, area-level deprivation, smoking status and BMI.

^b^Adjusted for age, region, area-level deprivation, smoking status and BMI.

^c^Adjusted for age, ethnicity, region, and smoking status and BMI.

^d^Adjusted for age, ethnicity, area-level deprivation, smoking status and BMI.

^e^Adjusted for age, ethnicity, region, area-level deprivation and BMI.

^f^Adjusted for age, region, ethnicity, area-level deprivation and smoking status.

**Table 4:** Variation in cervical cancer screening participation, for adults with and without SMIs, by demographic characteristic, smoking status and BMI.

	Number of people		Proportion of eligible people who participated (95% CIs)		Unadjusted OR (95% CIs)	Adjusted OR (95% CIs)
	No SMI *N* (%)	SMI *N* (%)	No SMI	SMI		
Age^a^						
25–29	244,212 (9.87)	1745 (6.00)	57.30 (57.10, 57.49)	57.59 (55.23, 59.92)	1.01 (0.92, 1.11)	0.92 (0.84, 1.02)
30–34	296,599 (11.99)	2780 (9.55)	68.49 (68.32, 68.65)	66.69 (64.90, 68.44)	0.92* (0.85, 1.00)	0.85*** (0.79, 0.92)
35–39	325,903 (13.18)	3362 (11.55)	70.72 (70.57, 70.88)	66.69 (65.06, 68.27)	0.83*** (0.77, 0.89)	0.83*** (0.77, 0.89)
40–44	327,468 (13.24)	3782 (13.00)	73.01 (72.85, 73.16)	65.89 (64.35, 67.40)	0.71*** (0.67, 0.76)	0.75*** (0.70, 0.80)
45–49	338,905 (13.70)	4467 (15.35)	72.36 (72.21, 72.51)	63.98 (62.55, 65.39)	0.68*** (0.64, 0.72)	0.72*** (0.68, 0.76)
50–54	335,234 (13.55)	4364 (15.00)	77.76 (77.62, 77.90)	69.84 (68.45, 71.20)	0.66*** (0.62, 0.71)	0.70*** (0.65, 0.74)
55–59	327,923 (13.26)	4710 (16.18)	69.61 (69.45, 69.77)	63.04 (61.64, 64.41)	0.74*** (0.70, 0.79)	0.78*** (0.74, 0.83)
60–64	277,313 (11.21)	3892 (13.37)	64.13 (63.96, 64.31)	56.53 (54.95, 58.09)	0.73*** (0.68, 0.78)	0.76*** (0.71, 0.81)
Ethnicity^b^						
White	1,645,992 (66.54)	19,964 (68.60)	72.93 (72.86, 73.00)	65.95 (65.29, 66.61)	0.72*** (0.7, 0.74)	0.78*** (0.75, 0.80)
Asian	201,340 (8.14)	2272 (7.81)	61.79 (61.58, 62.01)	57.92 (55.86, 59.96)	0.85*** (0.78, 0.93)	0.71*** (0.65, 0.77)
Black	106,633 (4.31)	2173 (7.47)	69.46 (69.18, 69.73)	64.80 (62.74, 66.80)	0.81*** (0.74, 0.88)	0.71*** (0.65, 0.78)
Mixed	36,724 (1.48)	690 (2.37)	67.23 (66.75, 67.71)	64.49 (60.78, 68.04)	0.89 (0.76, 1.04)	0.81* (0.69, 0.95)
Other	28,087 (1.14)	274 (0.94)	59.68 (59.10, 60.25)	62.04 (55.98, 67.76)	1.10 (0.86, 1.41)	0.88 (0.69, 1.14)
Unknown	454,781 (18.39)	3729 (12.81)	62.52 (62.38, 62.66)	57.98 (56.37, 59.57)	0.83*** (0.77, 0.88)	0.81*** (0.76, 0.87)
Deprivation^c^						
Quintile 1 (Least deprived)	496,894 (20.09)	3671 (12.61)	75.14 (75.02, 75.26)	70.14 (68.63, 71.62)	0.78*** (0.72, 0.83)	0.77*** (0.71, 0.82)
Quintile 2	463,336 (18.73)	4114 (14.14)	72.58 (72.45, 72.71)	65.75 (64.27, 67.20)	0.73*** (0.68, 0.77)	0.70*** (0.66, 0.75)
Quintile 3	451,718 (18.26)	4986 (17.13)	69.86 (69.73, 69.99)	63.76 (62.40, 65.09)	0.76*** (0.72, 0.80)	0.74*** (0.70, 0.78)
Quintile 4	504,382 (20.39)	6926 (23.80)	66.52 (66.39, 66.65)	63.80 (62.66, 64.93)	0.89*** (0.84, 0.93)	0.83*** (0.79, 0.87)
Quintile 5 (Most deprived)	474,147 (19.17)	8362 (28.73)	64.59 (64.45, 64.72)	61.47 (60.41, 62.51)	0.87*** (0.84, 0.91)	0.82*** (0.78, 0.85)
Unknown	83,080 (3.36)	1043 (3.58)	69.44 (69.12, 69.75)	62.32 (59.29, 65.26)	0.73*** (0.64, 0.83)	0.74*** (0.65, 0.84)
Region^d^						
North East	82,770 (3.35)	1028 (3.53)	71.4 (71.09, 71.710)	61.58 (58.52, 64.55)	0.64*** (0.57, 0.73)	0.66*** (0.58, 0.75)
North West	443,253 (17.92)	5956 (20.47)	69.58 (69.45, 69.72)	64.19 (62.95, 65.40)	0.78*** (0.74, 0.83)	0.81*** (0.76, 0.85)
Yorkshire and Humber	84,418 (3.41)	890 (3.06)	71.22 (70.91, 71.52)	65.39 (62.15, 68.50)	0.76*** (0.66, 0.88)	0.81** (0.70, 0.93)
East Midlands	47,333 (1.91)	498 (1.71)	73.63 (73.23, 74.02)	68.47 (64.16, 72.50)	0.78** (0.64, 0.94)	0.80* (0.66, 0.97)
West Midlands	392,267 (15.86)	4168 (14.32)	69.79 (69.65, 69.94)	63.84 (62.36, 65.30)	0.76*** (0.72, 0.81)	0.79*** (0.74, 0.84)
East of England	107,474 (4.34)	1076 (3.70)	72.3 (72.03, 72.570)	66.08 (63.15, 68.89)	0.75*** (0.66, 0.85)	0.75*** (0.66, 0.85)
South West	285,547 (11.54)	2870 (9.86)	72.42 (72.25, 72.58)	66.69 (64.93, 68.41)	0.76*** (0.71, 0.82)	0.79*** (0.73, 0.85)
South Central	309,843 (12.53)	3349 (11.51)	71.22 (71.06, 71.37)	65.09 (63.45, 66.71)	0.75*** (0.70, 0.81)	0.77*** (0.71, 0.82)
London	510,476 (20.64)	6919 (23.77)	65.29 (65.16, 65.42)	62.51 (61.35, 63.65)	0.89*** (0.84, 0.93)	0.75*** (0.72, 0.79)
South East Coast	210,176 (8.50)	2348 (8.07)	71.34 (71.15, 71.53)	63.80 (61.81, 65.74)	0.71*** (0.65, 0.77)	0.72*** (0.66, 0.79)
Smoking status^e^						
Non-smoker	1,595,922 (64.52)	12,605 (43.31)	69.91 (69.84, 69.98)	63.22 (62.37, 64.06)	0.74*** (0.71, 0.77)	0.73*** (0.70, 0.75)
Smoker	877,635 (35.48)	16,497 (56.69)	69.39 (69.29, 69.49)	64.85 (64.12, 65.58)	0.81*** (0.79, 0.84)	0.83*** (0.80, 0.86)
BMI^f^						
BMI < 30	2,347,395 (94.90)	25,633 (88.08)	69.67 (69.61, 69.73)	63.89 (63.30, 64.48)	0.77*** (0.75, 0.79)	0.78*** (0.76, 0.80)
BMI > 30	126,162 (5.10)	3469 (11.92)	70.72 (70.47, 70.97)	66.04 (64.43, 67.61)	0.81*** (0.75, 0.86)	0.81*** (0.75, 0.87)

*P < 0.05, **p < 0.01, ***p < 0.001.

^a^Adjusted for ethnicity, region, area-level deprivation, smoking status and BMI.

^b^Adjusted for age, region, area-level deprivation, smoking status and BMI.

^c^Adjusted for age, ethnicity, region, and smoking status and BMI.

^d^Adjusted for age, ethnicity, area-level deprivation, smoking status and BMI.

^e^Adjusted for age, ethnicity, region, area-level deprivation and BMI.

^f^Adjusted for age, region, ethnicity, area-level deprivation and smoking status.

#### Age

Inequalities in bowel cancer screening participation, between people with and without SMI, increased with age (the mean difference in participation between people with and without SMI increased from 12.1 percentage points at ages 60–64 [36.60% vs. 48.70%; aOR: 0.68, 95% CI: 0.65, 0.71], to 19.5 percentage points at ages 70–74 years [46.97% vs. 66.42%; aOR: 0.50, 95% CI: 0.47, 0.52]).

Patterns were similar for cervical screening, with inequalities in participation increasing with age (the mean difference in participation, between people with and without SMI, increased from 0.29 percentage points at ages 25–29 years [57.59% vs. 57.30%; aOR: 0.92, 95% CI: 0.84, 1.02], to 7.6 percentage points at ages 60–64 years [56.53% vs. 64.13%; aOR: 0.76, 95% CI: 0.71, 0.81]).

Patterns were slightly different for breast cancer screening, with differences in participation remaining relatively stable with age (the mean difference in participation, between people with and without a SMI, increased from 10.1 percentage points at ages 50–54 [40.88% vs. 51.03%; aOR: 0.74, 95% CI: 0.70, 078], to 11.3 percentage points at ages 70–74 years [53.36% vs. 64.69%; aOR: 0.70, 95% CI: 0.60, 0.82]).

#### Sex

Inequalities in bowel cancer screening participation, between people with and without SMI, were similar for both sexes (the mean difference in participation between men with and without SMI was 16.5 percentage points [39.14% vs. 56.48%; aOR: 0.57, 95% CI: 0.55, 0.59]; the mean difference in participation between women with and without SMI was also 16.5 percentage points [44.74% vs. 61.26%; aOR: 0.58, 95% CI: 0.55, 0.60]).

Men with SMI were notably less likely to participate in bowel cancer screening than women with SMI (39.14% vs. 44.74%).

#### Ethnicity

Participation by adults of an ethnic minority background was notably lower for all three screening programmes and was particularly low for those of an ethnic minority group who had SMI (see Tables 2–4). For bowel cancer screening, participation was lowest among Black adults (34.86% and 47.23% for those with and without SMI, respectively) and highest among White adults (44.01% and 62.23% for those with and without SMI, respectively). For breast screening, similarly, participation was also lowest for Black women (38.68% and 44.95% for those with and without SMI, respectively) and highest for White women (50.53% and 62.98% for those with and without SMI, respectively). Results were different for cervical screening, with participation being lowest among Asian women within the SMI cohort, and Other women among those without SMI (participation was 57.92% and 59.68%, respectively). Again, participation in cervical screening was highest among White women (participation was 65.95% and 72.93% among those with and without SMI, respectively).

After adjusting for the impact of other factors, adults with SMI, across all ethnic groups, show significantly lower participation for all three cancer screening programmes. Adults with SMI from White ethnic groups show higher screening participation than their peers with SMI from other ethnic groups, but the difference in participation, between those with and without SMI, was consistently wider for White adults than any other ethnic group. For bowel cancer screening, there was an 18 percentage points difference in participation between White adults, with and without SMI (44.01% vs. 62.23%; aOR: 0.56, 95% CI: 0.54, 0.58), compared to an 11 percentage points difference for those of a Mixed ethnic background (39.49% vs. 50.99% [aOR: 0.71, 95% CI: 0.56, 0.92])—the ethnic group with the smallest difference in participation, between those with and without SMI. Patterns were slightly different for breast and cervical screening with the absolute differences between adults with and without SMI for each ethnic group smaller. For breast cancer, the greatest difference in participation, between those with and without SMI, remained between White adults (50.53% vs. 62.98% [aOR: 0.67, 95% CI: 0.65, 0.69]), and the smallest being between those of Other ethnicity (43.70% vs. 46.41% [aOR: 0.87, 95% CI: 0.61, 1.23]). Similar to breast, for cervical screening, the greatest difference in participation, between those with and without SMI, was among White adults (65.95% vs. 72.93% [aOR: 0.78, 95% CI: 0.75, 0.80]), and the smallest was for adults of Other ethnicity (62.04% vs. 59.68% [aOR: 0.88, 95% CI: 0.69, 1.14]).

#### Area-level deprivation

For all three screening programmes, participation was lowest among people living in the most deprived quintile of areas, both for the SMI and non-SMI populations (Bowel: 36.17% and 47.86%, respectively; Breast: 40.23% vs. 50.36%; Cervical: 61.47% vs. 64.59%). Similar to ethnicity, after adjusting for the impact of other factors, adults with SMI across all deprivation quintiles show significantly lower participation for all three cancer screening programmes. Participation in bowel cancer screening, by deprivation quintile, was consistently 15 percentage points lower among those with SMI, with the exception of the most deprived quintile, where the difference in participation, between those with and without SMI, was around 11 percentage points [47.86% vs. 36.17%; aOR: 0.64, 95% CI: 0.60, 0.67]. For cervical screening inequality increased with increasing deprivation, with the difference in participation, between women with and without SMI, being 5.00 percentage points in the least deprived quintile of areas, and 7.12 percentage points in the most deprived quintile of areas. As with cervical screening, differences in participation for breast cancer screening differences, between adults with and without SMI, widened gradually, as deprivation increased (from 8.08 percentage points [67.54 vs. 59.46%; aOR: 0.69, 95% CI: 0.64, 0.75] in the least deprived quintile, to around 10.61 percentage points [55.26 vs. 44.65%; aOR: 0.68, 95% CI: 0.63, 0.72] in the second most deprived quintile [and the difference narrowing again for the most deprived quintile of areas]).

#### Region

Participation for all three screening programmes was lowest in London, both for people with and without SMI (participation in bowel cancer screening, for people with and without SMI, living in London, was 37.66% and 52.33%, respectively; participation in breast cancer screening, for people with and without SMI, living in London, was 38.50% and 49.06%, respectively; participation in cervical screening, for people with and without SMI, living in London, was 62.51% and 65.29%, respectively).

Across all regions in England, adults with SMI, compared to adults without SMI, experience significantly lower participation in all three cancer screening programmes, after adjusting for the impact of other factors. The widest regional inequalities in cancer screening participation, between people with and without SMI, were observed in the South West for bowel cancer screening, and the North East for breast and cervical screening (participation in bowel cancer screening, by people with and without SMI, living in the South West, was 43.19% and 60.27%, respectively [aOR: 0.55, 95% CI: 0.50, 0.59]; participation in breast cancer screening, by people with and without SMI, living in the North East, was 53.02% and 65.59%, respectively [aOR: 0.60, 95% CI: 0.51, 0.71]; participation in cervical screening, by people with and without SMI, living in the North East, was 61.58% and 71.40%, respectively, [aOR: 0.66, 95% CI: 0.58, 0.75]).

#### Smoking status

Participation in bowel, breast and cervical screening was lower among smokers than non-smokers, both for adults with and without SMI (see Tables 2–4). Inequalities in bowel cancer screening participation, between people with and without SMI, did not appear to be exacerbated by smoking status (there was a 15.5 percentage points difference in participation in bowel cancer screening between non-smokers, with and without SMI [47.00% vs. 62.47%; aOR: 0.55, 95% CI: 0.53, 0.58], and a 14.7 percentage points difference in participation between smokers [37.63% vs. 52.33% [aOR: 0.59, 95% CI: 0.57, 0.61]). The same was true for breast cancer screening (there was a 10.3 percentage points difference in participation in breast cancer screening [51.91% vs. 62.22%; aOR: 0.71, 95% CI: 0.68, 0.74], and a 12.0 percentage point difference in participation between smokers [45.18% vs. 57.18% [aOR: 0.66, 95% CI: 0.63, 0.69]) and cervical screening (there was a 6.7 percentage point difference in participation in cervical screening between non-smokers, with and without SMI [63.22% vs. 69.91%; aOR: 0.73, 95% CI: 0.70, 0.75], and a 4.5 percentage point difference in participation between smokers [64.85% vs. 69.39% [aOR: 0.83, 95% CI: 0.80, 0.86]).

#### BMI

For people with SMI, participation in bowel cancer screening was slightly higher among those with a BMI > 30 (see Table 2). Conversely, for people without SMI, it was slightly lower for those with a BMI > 30. As a result, differences in bowel cancer screening participation, between people with and without SMI, reduced with increased BMI (there was a 17.2 percentage point difference in participation in bowel cancer screening between people with a BMI < 30, [41.83% vs. 59.04%; aOR: 0.56, 95% CI: 0.54, 0.58], and a 12.6 percentage point difference in participation between people with a BMI > 30 [44.26% vs. 56.90% [aOR: 0.67, 95% CI: 0.62, 0.73]). With regards to breast cancer screening, participation was the same for people with a BMI < 30, as people with a BMI > 30, irrespective of whether they had SMI (see Table 3).

For cervical screening, participation did not vary by BMI for those without SMI. For people with SMI, however, participation was slightly lower among people with a BMI < 30 compared to those with a BMI > 30 (see Table 4). As with bowel screening, therefore, inequalities in cervical screening participation, between people with and without SMI, were slightly reduced with increasing BMI (there was a 5.8 percentage point difference in participation in cervical cancer screening among those with a BMI < 30 [63.89% vs. 69.67%; aOR: 0.78, 95% CI: 0.76, 0.80], and a 4.7 percentage point difference in participation between people with a BMI > 30 [66.04% vs. 70.72% [aOR: 0.81, 95% CI: 0.75, 0.87]).

## Discussion

### Summary of main findings

This study confirms that, in England, people with SMI are less likely to participate in breast, bowel and cervical screening, compared with people who do not have SMI. This study adds to current knowledge, showing that participation is lowest for people with schizophrenia, followed by people with other psychoses and people with bipolar disorder.

Importantly, this study shows that inequalities in participation, between those with and without SMI, persist across sociodemographic subgroups, such as age and sex, and are exacerbated by several key characteristics. Specifically, inequalities in participation, between people with and without SMI, increased with age for breast cancer screening, while for bowel and cervical screening, inequalities increased with area-level deprivation (it should be noted that, as deprivation increases, the proportion of the population that have SMI also increases) [15].

When considering variation by ethnicity, it was found that adults with SMI, who were of an ethnic minority group, had lower participation than their White counterparts. Adults with SMI from Black ethnic groups had the lowest participation in bowel and breast screening, while female adults with SMI, from Asian ethnic groups, had the lowest participation in cervical screening. The widest gaps in participation, between people with and without SMI, however, were observed in the White population. Despite this, adults of an ethnic minority background were often less likely to participate in screening if they did not have SMI, than White adults with SMI, highlighting the width of the gap in screening participation between those of a White and non-White ethnicity.

### Comparison with previous literature

The findings of this research are consistent with a previous study by PHE [15], as well as a recent meta-analysis of global studies [30], both of which found participation in cancer screening programmes to be lower among people with SMI. The present study builds upon these studies’ findings, however, by highlighting that schizophrenia, bipolar disorder and other psychoses are differentially associated with screening participation, and that screening participation is lowest among people with schizophrenia. Our study also builds upon these studies’ findings by highlighting that inequalities in participation, between people with and without SMI, exist across all sociodemographic strata, are exacerbated by age (breast) and area-level deprivation (bowel and cervical), and are independent of IMD and ethnicity [15, 17, 31].

Our findings are also consistent with previous studies documenting lower screening participation in London (observed even after adjusting for population characteristics) [32]. As speculated by others [33], the increased mobility of people in London may artificially lower the observed participation for this region (through a greater number of invitations being sent to out of date addresses).

### Implications for policy and future research

The results of this study have implications for future research. Specifically, they highlight the need for qualitative research to better understand why people with SMI (particularly those from an ethnic minority background, or who live in socioeconomically deprived areas) are less likely to participate in all three cancer screening programmes. Such research is needed to support the development and co-production of interventions to address the intrapersonal, individual-level and organisational barriers to screening experienced by these groups (previous qualitative research has been conducted in the UK, but has not focussed on ethnic and socioeconomic subgroups with the lowest participation, specifically [16]). In addition, further research, focussing on GP visits, may provide further insights into why people with SMI are diagnosed at a later stage, and possible opportunities for early diagnosis [34].

The results of this study also have implications for policy. Indeed, they support the NHS Long Term Plan, which set the ambition to increase access to annual physical health checks, for adults with SMI, with the aim of 390,000 people receiving physical health checks by 2023/2024 (access to national screening programmes is outlined as an additional element of this comprehensive annual health assessment, and provides an opportunity for intervention and support to improve screening access for this group [for example, by allowing staff to identify any ‘reasonable adjustments’ that the patient may require, in order to participate in screening, such as the need for a separate waiting area, by using a decisional conflict aid, as has been done for cervical screening [35]]) [36–38]. They also support the NHS Core 20PLUS5 initiative (an approach to reducing health inequalities), which describes five focus clinical areas requiring accelerated improvement, including the most deprived 20% of the national population (as identified by the national IMD), ethnic minority communities, SMI and early cancer diagnosis (i.e. by highlighting the need for improved cancer screening across these clinical areas) [38].

The results also highlight the need for specific action to support people with schizophrenia, as well as the need for extra support for people with SMI living in socioeconomically deprived and/or ethnically diverse areas (people with SMI in these areas are the least likely to participate in screening, and the prevalence of SMI in these areas is also greatest, as is premature mortality in people with SMI) [18, 39–42]. This is particularly true for those who smoke and have an elevated BMI, as both characteristics are associated with increased cancer incidence [43, 44].

Finally, the results support the need for routine data linkage to allow analysis (and monitoring of inequalities) to be carried out as routine, rather than having to rely on sample data, such as CPRD. This could build on the success and expansion of the Health and Care of People with Learning Disabilities collections [45], where population with SMI are currently flagged as a comparator group, but reporting of health outcomes (including cancer screening participation) for SMI cohort is not carried out.

### Strengths and limitations

This study has several strengths. First, the sample size was large, with over 1.7 million, 1.3 million and 2.5 million participants included in the breast, bowel and cervical screening analyses, respectively. Second, it used objective measures of screening participation (as opposed to self-reported measures), improving the validity of the findings. Finally, the effects of a wide range of known factors were included in the multivariate analysis, minimising the risk that the associations observed were due to external factors.

This study also has several limitations. First, it was restricted to data available within CPRD, meaning that it was not possible to include psychological variables (such as perceived risk of cancer)/additional demographic factors (such as employment status and educational attainment), or identify populations no longer residing at their home address (such as those in contact with the justice system), both of which are known to affect participation in screening. Second, adults with SMI, who are inpatients in mental health hospitals, for 6 months or more, are removed from the GP register and, therefore, were not included in our analysis (our study may, consequently, underestimate inequalities; further research exploring cancer screening use in these individuals would be valuable, given that SMI severity has been linked with participation, and those not registered with a GP do not receive screening invitations [46]). Third, the study period includes the initial months of the COVID-19 pandemic, and so overall participation may be lower because of this, and inequalities in participation exacerbated. Fourth, at the point of data extraction, no method for identifying transgender adults, within CPRD, had been described, and so it was not possible for us to explore whether inequalities disproportionately affect this population (such methods have since been described, and should be employed in future analyses [Trans men (assigned female at birth) do not receive invitations if registered as male with their GP, but are still entitled to screening if they have a cervix]) [47]. Fifth, an area-level and composite measure of socioeconomic deprivation was used, meaning that it was not possible to determine which specific aspects of socioeconomic deprivation (e.g. housing, education, employment, etc.) are most likely to impact on people with SMI (this also does not account for the way an individual experiences socioeconomic deprivation within the area they live; further research is needed to address this). Finally, this study only examined participation during a single round of screening. As such, it is not clear to what extent patterns in participation (i.e. regular participation, partial participation and regular non-participation [48]) vary between people with and without SMI. Given that these patterns have been shown to be associated with bowel cancer outcomes [49], further research, investigating these, would be valuable.

## Conclusions

This study highlights the complexity of inequality in participation in cancer screening, within and between populations with and without SMI. It confirms that participation is lower in those with SMI and highlights it is particularly low among those with schizophrenia, those living in the most deprived areas (with SMI), and those of a Black or Asian ethnicity. The results, consequently, indicate which conditions and population groups should receive extra support.

## Supplementary information

Appendix 1

Appendix 2

## Data Availability

CPRD data are available through a formal data request process.

## Reporting guidelines

This manuscript was written in accordance with the Strengthening of Reporting of Observational Studies in Epidemiology (STROBE) guidelines.
