# Supplementary material for: Inequalities in cancer screening participation between adults with and without severe mental illness: results from a cross-sectional analysis of primary care data on English Screening Programmes
Source: Br J Cancer. 2023 May 4;129(1):81–93. doi: 10.1038/s41416-023-02249-3 (PMC10307861; doi:10.1038/s41416-023-02249-3)
Supplement: Supplementary file 1 — Appendix 1 [file 41416_2023_2249_MOESM1_ESM.pdf]

This document lists the current version (20/08/2021) of the code lists associated with ISAC proposal 21_000523. It includes tables for:

Table 1: ScreeningProgram_SNOMED 1
Table 2: ScreeningProgram_Read 13
Table 3: SMIdiagnosis_SNOMED_Read_EMIS 18
Table 4: Ethnicity_SNOMED_Read_EMIS 49
Table 5: BMIover30_SNOMED 74
Table 6: CurrentSmoker_SNOMED 75

The content of the tables is as follows:

- ScreeningProgram_SNOMED
    - (SNOMED codes to identify observations of bowel, breast and cervical screening)
- ScreeningProgram_Read
    - (Read codes to identify observations of bowel, breast and cervical screening)
- SMIdiagnosis_SNOMED_Read_EMIS
    - (SNOMED, Read and EMIS codes to identify diagnoses of severe mental illnesses)
- Ethnicity_SNOMED_Read_EMIS
    - (SNOMED, Read and EMIS codes to identify observations about ethnicity)
- BMIover30_SNOMED
    - (SNOMED codes to identify where someone has been recorded with a body mass index greater than 30)
- CurrentSmoker_SNOMED
    - (SNOMED codes to identify where someone has been recorded as a current smoker)

The primary lists for screening programmes, BMI and smoking status have developed from SNOMED codes. A supplementary list of Read and EMIS codes has been created for the screening programmes, and will be completed for the BMI and smoking statuses.

Table 1: ScreeningProgram_SNOMED

Cluster_ID	Cluster_description	SNOMED_code	SNOMED_code_description	PCD_Refset_ID	Service_and_Ruleset
SMEAR_COD	Cervical screening codes	1020451000000104	Cervical cytology test (observable entity)	999007251000230109	INLIQ Mental health | Other LDO | PLE GDPPR | PLE PHSMI | QOF Cervical screening
SMEAR_COD	Cervical screening codes	1104601000000107	Cervical smear screening done by other healthcare provider (situation)	999007251000230109	INLIQ Mental health | Other LDO | PLE GDPPR | PLE PHSMI | QOF Cervical screening
SMEAR_COD	Cervical screening codes	168406009	Severe dyskaryosis on cervical smear cannot exclude invasive carcinoma (finding)	999007251000230109	INLIQ Mental health | Other LDO | PLE GDPPR | PLE PHSMI | QOF Cervical screening
SMEAR_COD	Cervical screening codes	168407000	Cannot exclude glandular neoplasia on cervical smear (finding)	999007251000230109	INLIQ Mental health | Other LDO | PLE GDPPR | PLE PHSMI | QOF Cervical screening
SMEAR_COD	Cervical screening codes	168408005	Cervical smear - atrophic changes (finding)	999007251000230109	INLIQ Mental health | Other LDO | PLE GDPPR | PLE PHSMI | QOF Cervical screening
SMEAR_COD	Cervical screening codes	168410007	Cervical smear - borderline changes (finding)	999007251000230109	INLIQ Mental health | Other LDO | PLE GDPPR | PLE PHSMI | QOF Cervical screening
SMEAR_COD	Cervical screening codes	168411006	Cervical smear - endocervical cells present (finding)	999007251000230109	INLIQ Mental health | Other LDO | PLE GDPPR | PLE PHSMI | QOF Cervical screening
SMEAR_COD	Cervical screening codes	168414003	Cervical smear - inflammatory change (finding)	999007251000230109	INLIQ Mental health | Other LDO | PLE GDPPR

Cluster_ID	Cluster_description	SNOMED_code	SNOMED_code_description	PCD_Refset_ID	Service_and_Ruleset
					| PLE PHSMI | QOF Cervical screening
SMEAR_COD	Cervical screening codes	168415002	Cervical smear - no inflammation (finding)	999007251000230109	INLIQ Mental health | Other LDO | PLE GDPPR | PLE PHSMI | QOF Cervical screening
SMEAR_COD	Cervical screening codes	168416001	Cervical smear - severe inflammation (finding)	999007251000230109	INLIQ Mental health | Other LDO | PLE GDPPR | PLE PHSMI | QOF Cervical screening
SMEAR_COD	Cervical screening codes	168417005	Cervical smear - trichomonas (finding)	999007251000230109	INLIQ Mental health | Other LDO | PLE GDPPR | PLE PHSMI | QOF Cervical screening
SMEAR_COD	Cervical screening codes	168418000	Cervical smear - candida (finding)	999007251000230109	INLIQ Mental health | Other LDO | PLE GDPPR | PLE PHSMI | QOF Cervical screening
SMEAR_COD	Cervical screening codes	168421003	Cervical smear - herpes (finding)	999007251000230109	INLIQ Mental health | Other LDO | PLE GDPPR | PLE PHSMI | QOF Cervical screening
SMEAR_COD	Cervical screening codes	168422005	Cervical smear - actinomyces (finding)	999007251000230109	INLIQ Mental health | Other LDO | PLE GDPPR | PLE PHSMI | QOF Cervical screening
SMEAR_COD	Cervical screening codes	168423000	Cervical smear - gardnerella (finding)	999007251000230109	INLIQ Mental health | Other LDO | PLE GDPPR | PLE PHSMI | QOF Cervical screening

Cluster_ID	Cluster_description	SNOMED_code	SNOMED_code_description	PCD_Refset_ID	Service_and_Ruleset
SMEAR_COD	Cervical screening codes	168424006	Cervical smear - koilocytosis (finding)	999007251000230109	INLIQ Mental health | Other LDO | PLE GDPPR | PLE PHSMI | QOF Cervical screening
SMEAR_COD	Cervical screening codes	168428009	Cervical smear - action needed (situation)	999007251000230109	INLIQ Mental health | Other LDO | PLE GDPPR | PLE PHSMI | QOF Cervical screening
SMEAR_COD	Cervical screening codes	168436000	Cervical smear: colposcopy needed (situation)	999007251000230109	INLIQ Mental health | Other LDO | PLE GDPPR | PLE PHSMI | QOF Cervical screening
SMEAR_COD	Cervical screening codes	168437009	Cervical smear - cervical biopsy needed (situation)	999007251000230109	INLIQ Mental health | Other LDO | PLE GDPPR | PLE PHSMI | QOF Cervical screening
SMEAR_COD	Cervical screening codes	168438004	Cervical smear: uterine curettage needed (situation)	999007251000230109	INLIQ Mental health | Other LDO | PLE GDPPR | PLE PHSMI | QOF Cervical screening
SMEAR_COD	Cervical screening codes	168469000	Vaginal vault smear negative (finding)	999007251000230109	INLIQ Mental health | Other LDO | PLE GDPPR | PLE PHSMI | QOF Cervical screening
SMEAR_COD	Cervical screening codes	168471000	Vaginal vault smear - atrophic (finding)	999007251000230109	INLIQ Mental health | Other LDO | PLE GDPPR | PLE PHSMI | QOF Cervical screening
SMEAR_COD	Cervical screening codes	168472007	Vaginal vault smear abnormal (finding)	999007251000230109	INLIQ Mental health | Other LDO | PLE GDPPR

Cluster_ID	Cluster_description	SNOMED_code	SNOMED_code_description	PCD_Refset_ID	Service_and_Ruleset
					| PLE PHSMI | QOF Cervical screening
SMEAR_COD	Cervical screening codes	171149006	Screening for malignant neoplasm of cervix (procedure)	999007251000230109	INLIQ Mental health | Other LDO | PLE GDPPR | PLE PHSMI | QOF Cervical screening
SMEAR_COD	Cervical screening codes	171155001	Cancer cervix screening - up-to-date (finding)	999007251000230109	INLIQ Mental health | Other LDO | PLE GDPPR | PLE PHSMI | QOF Cervical screening
SMEAR_COD	Cervical screening codes	171160002	Ca cervix screening normal (finding)	999007251000230109	INLIQ Mental health | Other LDO | PLE GDPPR | PLE PHSMI | QOF Cervical screening
SMEAR_COD	Cervical screening codes	171161003	Ca cervix screening abnormal (finding)	999007251000230109	INLIQ Mental health | Other LDO | PLE GDPPR | PLE PHSMI | QOF Cervical screening
SMEAR_COD	Cervical screening codes	171162005	Cancer cervix screening and fee claim (finding)	999007251000230109	INLIQ Mental health | Other LDO | PLE GDPPR | PLE PHSMI | QOF Cervical screening
SMEAR_COD	Cervical screening codes	177072002	Examination of female genital tract under anesthetic and papanicolau smear (procedure)	999007251000230109	INLIQ Mental health | Other LDO | PLE GDPPR | PLE PHSMI | QOF Cervical screening
SMEAR_COD	Cervical screening codes	250538001	Dyskaryosis on cervical smear (finding)	999007251000230109	INLIQ Mental health | Other LDO | PLE GDPPR | PLE PHSMI | QOF Cervical screening

Cluster_ID	Cluster_description	SNOMED_code	SNOMED_code_description	PCD_Refset_ID	Service_and_Ruleset
SMEAR_COD	Cervical screening codes	252991009	Cervical intraepithelial neoplasia grade III with severe dysplasia (disorder)	999007251000230109	INLIQ Mental health | Other LDO | PLE GDPPR | PLE PHSMI | QOF Cervical screening
SMEAR_COD	Cervical screening codes	268408009	Vaginal smear biopsy (procedure)	999007251000230109	INLIQ Mental health | Other LDO | PLE GDPPR | PLE PHSMI | QOF Cervical screening
SMEAR_COD	Cervical screening codes	268543007	Cancer cervix - screening done (finding)	999007251000230109	INLIQ Mental health | Other LDO | PLE GDPPR | PLE PHSMI | QOF Cervical screening
SMEAR_COD	Cervical screening codes	269958004	Cervical smear - negative (finding)	999007251000230109	INLIQ Mental health | Other LDO | PLE GDPPR | PLE PHSMI | QOF Cervical screening
SMEAR_COD	Cervical screening codes	269959007	Cervical smear - mild dyskaryosis (finding)	999007251000230109	INLIQ Mental health | Other LDO | PLE GDPPR | PLE PHSMI | QOF Cervical screening
SMEAR_COD	Cervical screening codes	269960002	Cervical smear - severe dyskaryosis (finding)	999007251000230109	INLIQ Mental health | Other LDO | PLE GDPPR | PLE PHSMI | QOF Cervical screening
SMEAR_COD	Cervical screening codes	269961003	Cervical smear - moderate dyskaryosis (finding)	999007251000230109	INLIQ Mental health | Other LDO | PLE GDPPR | PLE PHSMI | QOF Cervical screening
SMEAR_COD	Cervical screening codes	269963000	Cervical smear - viral inflammation unspecified (finding)	999007251000230109	INLIQ Mental health | Other LDO | PLE GDPPR

Cluster_ID	Cluster_description	SNOMED_code	SNOMED_code_description	PCD_Refset_ID	Service_and_Ruleset
					| PLE PHSMI | QOF Cervical screening
SMEAR_COD	Cervical screening codes	269964006	Cervical smear - wart virus (finding)	999007251000230109	INLIQ Mental health | Other LDO | PLE GDPPR | PLE PHSMI | QOF Cervical screening
SMEAR_COD	Cervical screening codes	275805003	Viral changes on cervical smear (finding)	999007251000230109	INLIQ Mental health | Other LDO | PLE GDPPR | PLE PHSMI | QOF Cervical screening
SMEAR_COD	Cervical screening codes	281101005	Smear: no abnormality detected - no endocervical cells (finding)	999007251000230109	INLIQ Mental health | Other LDO | PLE GDPPR | PLE PHSMI | QOF Cervical screening
SMEAR_COD	Cervical screening codes	285836003	Cervical intraepithelial neoplasia grade 1 (disorder)	999007251000230109	INLIQ Mental health | Other LDO | PLE GDPPR | PLE PHSMI | QOF Cervical screening
SMEAR_COD	Cervical screening codes	285838002	Cervical intraepithelial neoplasia grade 2 (disorder)	999007251000230109	INLIQ Mental health | Other LDO | PLE GDPPR | PLE PHSMI | QOF Cervical screening
SMEAR_COD	Cervical screening codes	309081009	Abnormal cervical smear (finding)	999007251000230109	INLIQ Mental health | Other LDO | PLE GDPPR | PLE PHSMI | QOF Cervical screening
SMEAR_COD	Cervical screening codes	310841002	Cervical smear - mild inflammation (finding)	999007251000230109	INLIQ Mental health | Other LDO | PLE GDPPR | PLE PHSMI | QOF Cervical screening

Cluster_ID	Cluster_description	SNOMED_code	SNOMED_code_description	PCD_Refset_ID	Service_and_Ruleset
SMEAR_COD	Cervical screening codes	310842009	Cervical smear - moderate inflammation (finding)	999007251000230109	INLIQ Mental health | Other LDO | PLE GDPPR | PLE PHSMI | QOF Cervical screening
SMEAR_COD	Cervical screening codes	391148009	Human papillomavirus test negative (finding)	999007251000230109	INLIQ Mental health | Other LDO | PLE GDPPR | PLE PHSMI | QOF Cervical screening
SMEAR_COD	Cervical screening codes	412715009	Cervical smear transformation zone cells present (finding)	999007251000230109	INLIQ Mental health | Other LDO | PLE GDPPR | PLE PHSMI | QOF Cervical screening
SMEAR_COD	Cervical screening codes	417036008	Liquid based cervical cytology screening (procedure)	999007251000230109	INLIQ Mental health | Other LDO | PLE GDPPR | PLE PHSMI | QOF Cervical screening
SMEAR_COD	Cervical screening codes	438633009	Sampling of vaginal vault for smear (procedure)	999007251000230109	INLIQ Mental health | Other LDO | PLE GDPPR | PLE PHSMI | QOF Cervical screening
SMEAR_COD	Cervical screening codes	439074000	Dysplasia on cervical smear (finding)	999007251000230109	INLIQ Mental health | Other LDO | PLE GDPPR | PLE PHSMI | QOF Cervical screening
SMEAR_COD	Cervical screening codes	439333006	Sampling of female genital tract for smear (procedure)	999007251000230109	INLIQ Mental health | Other LDO | PLE GDPPR | PLE PHSMI | QOF Cervical screening
SMEAR_COD	Cervical screening codes	439776006	Cervical Papanicolaou smear positive for malignant neoplasm (finding)	999007251000230109	INLIQ Mental health | Other LDO | PLE GDPPR

Cluster_ID	Cluster_description	SNOMED_code	SNOMED_code_description	PCD_Refset_ID	Service_and_Ruleset
					| PLE PHSMI | QOF Cervical screening
SMEAR_COD	Cervical screening codes	439888000	Abnormal cervical Papanicolaou smear (finding)	999007251000230109	INLIQ Mental health | Other LDO | PLE GDPPR | PLE PHSMI | QOF Cervical screening
SMEAR_COD	Cervical screening codes	439958008	Sampling of cervix for Papanicolaou smear (procedure)	999007251000230109	INLIQ Mental health | Other LDO | PLE GDPPR | PLE PHSMI | QOF Cervical screening
SMEAR_COD	Cervical screening codes	441087007	Atypical squamous cells of undetermined significance on cervical Papanicolaou smear (finding)	999007251000230109	INLIQ Mental health | Other LDO | PLE GDPPR | PLE PHSMI | QOF Cervical screening
SMEAR_COD	Cervical screening codes	441088002	Atypical squamous cells on cervical Papanicolaou smear cannot exclude high grade squamous intraepithelial lesion (finding)	999007251000230109	INLIQ Mental health | Other LDO | PLE GDPPR | PLE PHSMI | QOF Cervical screening
SMEAR_COD	Cervical screening codes	441094005	Atypical endocervical cells on cervical Papanicolaou smear (finding)	999007251000230109	INLIQ Mental health | Other LDO | PLE GDPPR | PLE PHSMI | QOF Cervical screening
SMEAR_COD	Cervical screening codes	441219009	Atypical glandular cells on cervical Papanicolaou smear (finding)	999007251000230109	INLIQ Mental health | Other LDO | PLE GDPPR | PLE PHSMI | QOF Cervical screening
SMEAR_COD	Cervical screening codes	441667007	Abnormal cervical Papanicolaou smear with positive human papillomavirus deoxyribonucleic acid test (finding)	999007251000230109	INLIQ Mental health | Other LDO | PLE GDPPR | PLE PHSMI | QOF Cervical screening

Cluster_ID	Cluster_description	SNOMED_code	SNOMED_code_description	PCD_Refset_ID	Service_and_Ruleset
SMEAR_COD	Cervical screening codes	62051000119105	Low grade squamous intraepithelial lesion on cervical Papanicolaou smear (finding)	999007251000230109	INLIQ Mental health | Other LDO | PLE GDPPR | PLE PHSMI | QOF Cervical screening
SMEAR_COD	Cervical screening codes	62061000119107	High grade squamous intraepithelial lesion on cervical Papanicolaou smear (finding)	999007251000230109	INLIQ Mental health | Other LDO | PLE GDPPR | PLE PHSMI | QOF Cervical screening
SMEAR_COD	Cervical screening codes	700399008	Cervical smear - borderline change in squamous cells (finding)	999007251000230109	INLIQ Mental health | Other LDO | PLE GDPPR | PLE PHSMI | QOF Cervical screening
SMEAR_COD	Cervical screening codes	700400001	Cervical smear - borderline change in endocervical cells (finding)	999007251000230109	INLIQ Mental health | Other LDO | PLE GDPPR | PLE PHSMI | QOF Cervical screening
SMEAR_COD	Cervical screening codes	767357000	Sampling of cervix for Papanicolaou smear done (situation)	999007251000230109	INLIQ Mental health | Other LDO | PLE GDPPR | PLE PHSMI | QOF Cervical screening
SMEAR_COD	Cervical screening codes	812331000000105	Cervical smear pus cells present (finding)	999007251000230109	INLIQ Mental health | Other LDO | PLE GDPPR | PLE PHSMI | QOF Cervical screening
SMEAR_COD	Cervical screening codes	812351000000103	Cervical smear red blood cells present (finding)	999007251000230109	INLIQ Mental health | Other LDO | PLE GDPPR | PLE PHSMI | QOF Cervical screening
SMEAR_COD	Cervical screening codes	813711000000108	Cervical smear epithelial cells absent (situation)	999007251000230109	INLIQ Mental health | Other LDO | PLE GDPPR

Cluster_ID	Cluster_description	SNOMED_code	SNOMED_code_description	PCD_Refset_ID	Service_and_Ruleset
					| PLE PHSMI | QOF Cervical screening
SMEAR_COD	Cervical screening codes	880941000000101	Cervical smear - low grade dyskaryosis (finding)	999007251000230109	INLIQ Mental health | Other LDO | PLE GDPPR | PLE PHSMI | QOF Cervical screening
SMEAR_COD	Cervical screening codes	887481000000104	Cervical smear - high grade dyskaryosis (moderate) (finding)	999007251000230109	INLIQ Mental health | Other LDO | PLE GDPPR | PLE PHSMI | QOF Cervical screening
SMEAR_COD	Cervical screening codes	887501000000108	Cervical smear - high grade dyskaryosis (severe) (finding)	999007251000230109	INLIQ Mental health | Other LDO | PLE GDPPR | PLE PHSMI | QOF Cervical screening
SMEAR_COD	Cervical screening codes	887521000000104	Cervical smear - high grade dyskaryosis with features of invasive squamous carcinoma (finding)	999007251000230109	INLIQ Mental health | Other LDO | PLE GDPPR | PLE PHSMI | QOF Cervical screening
SMEAR_COD	Cervical screening codes	887601000000107	Cervical smear - features of endocervical type glandular neoplasia (finding)	999007251000230109	INLIQ Mental health | Other LDO | PLE GDPPR | PLE PHSMI | QOF Cervical screening
SMEAR_COD	Cervical screening codes	887621000000103	Cervical smear - features of non-cervical type glandular neoplasia (finding)	999007251000230109	INLIQ Mental health | Other LDO | PLE GDPPR | PLE PHSMI | QOF Cervical screening
SMEAR_COD	Cervical screening codes	888091000000108	Cervical smear - human papillomavirus negative (finding)	999007251000230109	INLIQ Mental health | Other LDO | PLE GDPPR | PLE PHSMI | QOF Cervical screening

Cluster_ID	Cluster_description	SNOMED_code	SNOMED_code_description	PCD_Refset_ID	Service_and_Ruleset
SMEAR_COD	Cervical screening codes	888111000000103	Cervical smear - human papillomavirus positive (finding)	999007251000230109	INLIQ Mental health | Other LDO | PLE GDPPR | PLE PHSMI | QOF Cervical screening
SMEAR_COD	Cervical screening codes	98791000119102	Cytological evidence of malignancy on cervical Papanicolaou smear (finding)	999007251000230109	INLIQ Mental health | Other LDO | PLE GDPPR | PLE PHSMI | QOF Cervical screening
BRCANSCR_COD	Breast cancer screening codes	168749009	Mammography normal (finding)	999016171000230107	Other LDO | PLE GDPPR | PLE PHSMI
BRCANSCR_COD	Breast cancer screening codes	168750009	Mammography abnormal (finding)	999016171000230107	Other LDO | PLE GDPPR | PLE PHSMI
BRCANSCR_COD	Breast cancer screening codes	171175005	Breast neoplasm screening normal (finding)	999016171000230107	Other LDO | PLE GDPPR | PLE PHSMI
BRCANSCR_COD	Breast cancer screening codes	171176006	Breast neoplasm screening abnormal (finding)	999016171000230107	Other LDO | PLE GDPPR | PLE PHSMI
BRCANSCR_COD	Breast cancer screening codes	185710003	Breast screening abnormal - told patient (finding)	999016171000230107	Other LDO | PLE GDPPR | PLE PHSMI
BRCANSCR_COD	Breast cancer screening codes	24623002	Screening mammography (procedure)	999016171000230107	Other LDO | PLE GDPPR | PLE PHSMI
BRCANSCR_COD	Breast cancer screening codes	268547008	Screening for malignant neoplasm of breast (procedure)	999016171000230107	Other LDO | PLE GDPPR | PLE PHSMI
BRCANSCR_COD	Breast cancer screening codes	384151000119104	Screening mammography of bilateral breasts (procedure)	999016171000230107	Other LDO | PLE GDPPR | PLE PHSMI
BRCANSCR_COD	Breast cancer screening codes	392521000119107	Screening mammography of right breast (procedure)	999016171000230107	Other LDO | PLE GDPPR | PLE PHSMI
BRCANSCR_COD	Breast cancer screening codes	392531000119105	Screening mammography of left breast (procedure)	999016171000230107	Other LDO | PLE GDPPR | PLE PHSMI
BRCANSCR_COD	Breast cancer screening codes	455921000000104	[V]Screening for malignant neoplasm of breast (situation)	999016171000230107	Other LDO | PLE GDPPR | PLE PHSMI

Cluster_ID	Cluster_description	SNOMED_code	SNOMED_code_description	PCD_Refset_ID	Service_and_Ruleset
BRCANSCR_COD	Breast cancer screening codes	609223006	Magnetic resonance imaging of breast for screening for malignant neoplasm (procedure)	999016171000230107	Other LDO | PLE GDPPR | PLE PHSMI
BRCANSCR_COD	Breast cancer screening codes	71651007	Mammography (procedure)	999016171000230107	Other LDO | PLE GDPPR | PLE PHSMI
BRCANSCR_COD	Breast cancer screening codes	94361000000105	Breast cancer detected by national screening programme (disorder)	999016171000230107	Other LDO | PLE GDPPR | PLE PHSMI
COLCANSCR_COD	Colorectal cancer screening codes	375211000000108	Bowel cancer screening programme faecal occult blood test normal (finding)	999016251000230109	Other LDO | PLE GDPPR | PLE PHSMI
COLCANSCR_COD	Colorectal cancer screening codes	375241000000109	Bowel cancer screening programme faecal occult blood test abnormal (finding)	999016251000230109	Other LDO | PLE GDPPR | PLE PHSMI

Table 2: ScreeningProgram_Read

Category	Chapter	Code_main	Code_ext	Concat_Code	GenericDescription
Breast screening	Radiology & physics in medicine	537	0	537..00	Soft tissue X-ray breast
Breast screening	Radiology & physics in medicine	537	11	537..11	Mammography - X-ray
Breast screening	Radiology & physics in medicine	5372	0	5372	Mammography normal
Breast screening	Radiology & physics in medicine	5373	0	5373	Mammography abnormal
Breast screening	Radiology & physics in medicine	5376	0	5376	Mammography attended
Breast screening	Radiology & physics in medicine	537Z	0	537Z.00	Soft tissue X-ray breast NOS

Category	Chapter	Code_main	Code_ext	Concat_Code	GenericDescription
Breast screening	Preventative procedures	6795	0	6795	Health ed. - breast exam.
Breast screening	Preventative procedures	6862	0	6862	Breast neoplasm screen
Breast screening	Preventative procedures	6862	11	6862.11	Mammography - screening
Breast screening	Preventative procedures	68620	0	6862000	Breast neoplasm screen normal
Breast screening	Preventative procedures	68621	0	6862100	Breast neoplasm screen abnorm
Breast screening	Preventative procedures	6862Z	0	6862Z00	Breast neoplasm screen NOS
Breast screening	Preventative procedures	6865	0	6865	Breast neoplasm screen normal
Breast screening	Operations and procedures	7P0F	1	7P0F.01	Diagnostic imaging of breast
Breast screening	Operations and procedures	7P0F0	2	7P0F002	Scintimammography
Breast screening	Operations and procedures	7P0F1	3	7P0F103	Thermography of breast
Breast screening	Operations and procedures	7P0F2	0	7P0F200	Mammography
Breast screening	Operations and procedures	7P0Fy	4	7P0Fy04	Other specified diagnostic imaging of breast
Breast screening	Operations and procedures	7P0Fz	5	7P0Fz05	Diagnostic imaging of breast NOS
Breast screening	Administration	9OH9	0	9OH9.00	Breast screen abnorm.-told pat
Breast screening	Administration	9OHJ	0	9OHJ.00	Breast screening incomplete

Category	Chapter	Code_main	Code_ext	Concat_Code	GenericDescription
Breast screening	Administration	9Oq8	0	9Oq8.00	Attended breast screening clinic
Breast screening	[D] Symptoms, signs and ill-defined conditions	R138	0	R138.00	[D]Breast imaging abnormal
Breast screening	[D] Symptoms, signs and ill-defined conditions	R1380	0	R138000	[D]Mammogram abnormal
Breast screening	[D] Symptoms, signs and ill-defined conditions	R138z	0	R138z00	[D]Breast imaging abnormal NOS
Breast screening	Unspecified conditions	ZV761	0	ZV76100	[V]Screening for malignant neoplasm of breast
Breast screening	History & symptoms	1A84	0	1A84.00	Breast lump detected by mammogram
Cervical screening	Laboratory procedures	4K22.00	0	4K22.00	Cervical smear: negative
Cervical screening	Laboratory procedures	4K22.11	11	4K22.11	Smear NAD - no endocervical cells
Cervical screening	Laboratory procedures	4K23.00	0	4K23.00	Cerv.smear: mild dyskaryosis
Cervical screening	Laboratory procedures	4K23.11	11	4K23.11	CIN I - mild dsykaryosis
Cervical screening	Laboratory procedures	4K24.00	0	4K24.00	Cerv.smear: severe dyskaryosis
Cervical screening	Laboratory procedures	4K24.11	11	4K24.11	CIN III - severe dsykaryosis
Cervical screening	Laboratory procedures	4K25.00	0	4K25.00	Cerv.smear:severe dysk.?inv.ca
Cervical screening	Laboratory procedures	4K26.00	0	4K26.00	Cervical smear: ? gland neopl.

Category	Chapter	Code_main	Code_ext	Concat_Code	GenericDescription
Cervical screening	Laboratory procedures	4K27.00	0	4K27.00	Cervical smear:atrophic change
Cervical screening	Laboratory procedures	4K27.11	11	4K27.11	Atrophic change on cerv.smear
Cervical screening	Laboratory procedures	4K28.00	0	4K28.00	Cerv.smear: mod.dyskaryosis
Cervical screening	Laboratory procedures	4K28.11	11	4K28.11	CIN II - moderate dsykaryosis
Cervical screening	Laboratory procedures	4K29.00	0	4K29.00	Cerv.smear: borderline changes
Cervical screening	Laboratory procedures	4K29000	0	4K29000	Cervical smear - borderline change in squamous cells
Cervical screening	Laboratory procedures	4K29100	0	4K29100	Cervical smear - borderline change in endocervical cells
Cervical screening	Laboratory procedures	4K2C.00	0	4K2C.00	Smear NAD - no endocervical cells
Cervical screening	Laboratory procedures	4K2F.00	0	4K2F.00	Cervical smear pus cells present
Cervical screening	Laboratory procedures	4K2G.00	0	4K2G.00	Cervical smear red blood cells present
Cervical screening	Laboratory procedures	4K2H.00	0	4K2H.00	Cervical smear epithelial cells absent
Cervical screening	Laboratory procedures	4K2J.00	0	4K2J.00	Cervical smear - low grade dyskaryosis
Cervical screening	Laboratory procedures	4K2K.00	0	4K2K.00	Cervical smear - high grade dyskaryosis (moderate)
Cervical screening	Laboratory procedures	4K2L.00	0	4K2L.00	Cervical smear - high grade dyskaryosis (severe)
Cervical screening	Laboratory procedures	4K2M.00	0	4K2M.00	Cervical smear - high grade dyskaryosis with features of invasive squamous carcinoma

Category	Chapter	Code_main	Code_ext	Concat_Code	GenericDescription
Cervical screening	Laboratory procedures	4K2N.00	0	4K2N.00	Cervical smear - features of endocervical type glandular neoplasia
Cervical screening	Laboratory procedures	4K2P.00	0	4K2P.00	Cervical smear - features of non-cervical type glandular neoplasia
Cervical screening	Laboratory procedures	4K2Q.00	0	4K2Q.00	Cervical smear - human papillomavirus negative
Cervical screening	Laboratory procedures	4K2R.00	0	4K2R.00	Cervical smear - human papillomavirus positive
Cervical screening	Laboratory procedures	4K3E.00	0	4K3E.00	HPV - Human papillomavirus test negative
Cervical screening	Laboratory procedures	4K55.00	0	4K55.00	Cervical cytology test
Cervical screening	Laboratory procedures	4KA1.00	0	4KA1.00	Vaginal vault smear negative
Cervical screening	Laboratory procedures	4KA3.00	0	4KA3.00	Vaginal vault smear-atrophic
Cervical screening	Laboratory procedures	4KA4.00	0	4KA4.00	Vaginal vault smear abnormal
Cervical screening	Preventative procedures	6856	0	6856	Ca cervix screen - up to date
Cervical screening	Preventative procedures	6859	0	6859	Ca cervix - screen done
Cervical screening	Preventative procedures	6859.11	11	6859.11	Cervical cytology examination
Cervical screening	Preventative procedures	685B.00	0	685B.00	Ca cervix screen normal
Cervical screening	Preventative procedures	685C.00	0	685C.00	Ca cervix screen abnormal
Cervical screening	Preventative procedures	685D.00	0	685D.00	Ca cervix screen + fee claim

Category	Chapter	Code_main	Code_ext	Concat_Code	GenericDescription
Cervical screening	Preventative procedures	685R.00	0	685R.00	Liquid based cervical cytology screening
Cervical screening	Operations and procedures	7E2A000	0	7E2A000	Examination of female genital tract under anaesthetic and Papanicolau smear
Cervical screening	Operations and procedures	7E2A200	0	7E2A200	Papanicolau smear NEC
Cervical screening	Operations and procedures	7E2A211	11	7E2A211	Cervical smear NEC
Cervical screening	Operations and procedures	7E2A300	0	7E2A300	Vaginal vault smear
Cervical screening	Unspecified conditions	ZV76200	0	ZV76200	[V]Screening for malignant neoplasm of cervix
Cervical screening	Unspecified conditions	ZV76211	11	ZV76211	[V]Routine Papanicolaou smear
Cervical screening	Unspecified conditions	ZV76212	12	ZV76212	[V]Routine cervical smear
Bowel screening		686A	00	686A.00	Bowel cancer screening programme faecal occult blood test normal
Bowel screening		686B	00	686B.00	Bowel cancer screening programme faecal occult blood test abnormal

Table 3: SMIdiagnosis_SNOMED_Read_EMIS

Code	Term	Group	ParentGroup	CodingSystem	medcodeid
E1005	Schizophrenia in remission	SCH	SMI	Read	9225016
E1032	Chronic paranoid schizophrenia	SCH	SMI	Read	52897013
E1022	Chronic catatonic schizophrenia	SCH	SMI	Read	114616017
E1001	Subchronic schizophrenia	SCH	SMI	Read	28758018
E1021	Subchronic catatonic schizophrenia	SCH	SMI	Read	71539017

Code	Term	Group	ParentGroup	CodingSystem	medcodeid
E13y1	Brief reactive psychosis	PSY	SMI	Read	10122017
E1031	Subchronic paranoid schizophrenia	SCH	SMI	Read	132503015
E1013	Acute exacerbation of subchronic hebephrenic schizophrenia	SCH	SMI	Read	294738017
E111	Recurrent manic episodes	BPD	SMI	Read	294810017
E115z	Bipolar affective disorder, currently depressed, NOS	BPD	SMI	Read	294868016
E120	Simple paranoid state	PSY	SMI	Read	294904016
E13y	Other reactive psychoses	PSY	SMI	Read	294926015
E1014	Acute exacerbation of chronic hebephrenic schizophrenia	SCH	SMI	Read	294739013
E102	Catatonic schizophrenia	SCH	SMI	Read	294742019
E1023	Acute exacerbation of subchronic catatonic schizophrenia	SCH	SMI	Read	294750011
E10yz	Other schizophrenia NOS	SCH	SMI	Read	294789017
E114z	Bipolar affective disorder, currently manic, NOS	BPD	SMI	Read	294858014
E116	Mixed bipolar affective disorder	BPD	SMI	Read	294869012
E123-1	Folie a deux	PSY	SMI	Read	294908018
E100z	Simple schizophrenia NOS	SCH	SMI	Read	294734015
E1106	Single manic episode in full remission	BPD	SMI	Read	294808019
E12z	Paranoid psychosis NOS	PSY	SMI	Read	294913019
E134	Psychogenic paranoid psychosis	PSY	SMI	Read	294924017
E100-1	Schizophrenia simplex	SCH	SMI	Read	294726014
E1103	Single manic episode, severe without mention of psychosis	BPD	SMI	Read	294805016
E1104	Single manic episode, severe, with psychosis	BPD	SMI	Read	294806015
E1142	Bipolar affective disorder, currently manic, moderate	BPD	SMI	Read	294850019
E1055	Latent schizophrenia in remission	PSY	SMI	Read	294770017
E1161	Mixed bipolar affective disorder, mild	BPD	SMI	Read	294871012

Code	Term	Group	ParentGroup	CodingSystem	medcodeid
E101z	Hebephrenic schizophrenia NOS	SCH	SMI	Read	294741014
E1024	Acute exacerbation of chronic catatonic schizophrenia	SCH	SMI	Read	294751010
E1102	Single manic episode, moderate	BPD	SMI	Read	294804017
E12y	Other paranoid states	PSY	SMI	Read	294910016
E1100	Single manic episode, unspecified	BPD	SMI	Read	294802018
E1160	Mixed bipolar affective disorder, unspecified	BPD	SMI	Read	294870013
E1162	Mixed bipolar affective disorder, moderate	BPD	SMI	Read	294872017
Eu23y	[X]Other acute and transient psychotic disorders	PSY	SMI	Read	296083012
E114-1	Manic-depressive - now manic	BPD	SMI	Read	294847017
E1140	Bipolar affective disorder, currently manic, unspecified	BPD	SMI	Read	294848010
E11yz	Other and unspecified manic-depressive psychoses NOS	BPD	SMI	Read	294896010
Eu22z	[X]Persistent delusional disorder, unspecified	PSY	SMI	Read	296066015
E10y-1	Cenesthopathic schizophrenia	SCH	SMI	Read	294788013
E1172	Unspecified bipolar affective disorder, moderate	BPD	SMI	Read	294883014
E1110	Recurrent manic episodes, unspecified	BPD	SMI	Read	294811018
E1151	Bipolar affective disorder, currently depressed, mild	BPD	SMI	Read	294862015
E11y3	Other mixed manic-depressive psychoses	BPD	SMI	Read	294895014
Eu20z	[X]Schizophrenia, unspecified	SCH	SMI	Read	296040018
E1054	Acute exacerbation of chronic latent schizophrenia	PSY	SMI	Read	294769018
E1052	Chronic latent schizophrenia	PSY	SMI	Read	294767016
E1176	Unspecified bipolar affective disorder, in full remission	BPD	SMI	Read	294887010
E11y1	Atypical manic disorder	BPD	SMI	Read	294893019
Eu25z	[X]Schizoaffective disorder, unspecified	PSY	SMI	Read	296096013
E102z	Catatonic schizophrenia NOS	SCH	SMI	Read	294753013

Code	Term	Group	ParentGroup	CodingSystem	medcodeid
E105	Latent schizophrenia	PSY	SMI	Read	294764011
E1166	Mixed bipolar affective disorder, in full remission	BPD	SMI	Read	294876019
E117	Unspecified bipolar affective disorder	BPD	SMI	Read	294880012
E1171	Unspecified bipolar affective disorder, mild	BPD	SMI	Read	294882016
E11z0	Unspecified affective psychoses NOS	BPD	SMI	Read	294898011
E10	Schizophrenic disorders	SCH	SMI	Read	294725013
E100	Simple schizophrenia	SCH	SMI	Read	294727017
E1012	Chronic hebephrenic schizophrenia	SCH	SMI	Read	294737010
E116z	Mixed bipolar affective disorder, NOS	BPD	SMI	Read	294877011
E11z	Other and unspecified affective psychoses	BPD	SMI	Read	294897018
Eu31z	[X]Bipolar affective disorder, unspecified	BPD	SMI	Read	296135011
E1000	Unspecified schizophrenia	SCH	SMI	Read	294728010
E1015	Hebephrenic schizophrenia in remission	SCH	SMI	Read	294740010
E10z	Schizophrenia NOS	SCH	SMI	Read	294790014
E1170	Unspecified bipolar affective disorder, unspecified	BPD	SMI	Read	294881011
E131	Acute hysterical psychosis	PSY	SMI	Read	294919015
E105z	Latent schizophrenia NOS	PSY	SMI	Read	294771018
E121-1	Sanders disease	PSY	SMI	Read	161371000006118
Eu233	[X]Other acute predominantly delusional psychotic disorders	PSY	SMI	Read	401859016
Eu31y	[X]Other bipolar affective disorders	BPD	SMI	Read	401865016
E1115	Recurrent manic episodes, partial or unspecified remission	BPD	SMI	Read	182861000006110
Eu30z	[X]Manic episode, unspecified	BPD	SMI	Read	401864017
Eu220-5	[X]Paranoia	PSY	SMI	Read	215871000000115
E107z	Schizo-affective schizophrenia NOS	SCH	SMI	Read	155161000006117
Eu220-1	[X]Paranoid psychosis	PSY	SMI	Read	215851000000112
286-1	Poor insight into psychotic condition	PSY	SMI	Read	215691000006112
Eu21	[X]Schizotypal disorder	PSY	SMI	Read	401856011

Code	Term	Group	ParentGroup	CodingSystem	medcodeid
E110	Manic disorder, single episode	BPD	SMI	Read	401765010
E1113	Recurrent manic episodes, severe without mention psychosis	BPD	SMI	Read	182871000006115
E10y	Other schizophrenia	SCH	SMI	Read	401764014
E1z	Non-organic psychosis NOS	PSY	SMI	Read	401771016
ZV110	[V]Personal history of schizophrenia	SCH	SMI	Read	460273017
Eu232	[X]Acute schizophrenia-like psychotic disorder	PSY	SMI	Read	362421000006113
Eu2z	[X]Unspecified nonorganic psychosis	PSY	SMI	Read	401862018
E12	Paranoid states	PSY	SMI	Read	243361000006117
E104	Acute schizophrenic episode	SCH	SMI	Read	401763015
E1175	Unspecified bipolar affect disord, partial/unspec remission	BPD	SMI	Read	82091000006116
ZV111-2	[V]Personal history of manic-depressive psychosis	BPD	SMI	Read	1227584015
Eu230-1	[X]Bouffee delirante	PSY	SMI	Read	367961000006110
Eu231-1	[X]Bouffee delirante with symptoms of schizophrenia	PSY	SMI	Read	367971000006115
Eu31	[X]Bipolar affective disorder	BPD	SMI	Read	367101000006112
Eu316	[X]Bipolar affective disorder, current episode mixed	BPD	SMI	Read	367121000006119
Eu312	[X]Bipolar affect disorder cur epi manic with psychotic symp	BPD	SMI	Read	367071000006119
Eu202	[X]Catatonic schizophrenia	SCH	SMI	Read	370451000006110
Eu21-3	[X]Latent schizophrenia	PSY	SMI	Read	395021000006111
E114	Bipolar affective disorder, currently manic	BPD	SMI	Read	513801000006112
Eu24-1	[X]Folie a deux	PSY	SMI	Read	387071000006117
E1155	Bipolar affect disord, now depressed, part/unspec remission	BPD	SMI	Read	513711000006118
E133-1	Bouffee delirante	PSY	SMI	Read	523481000006114
Eu31y-1	[X]Bipolar II disorder	BPD	SMI	Read	367161000006113
Eu20y-1	[X]Cenesthopathic schizophrenia	SCH	SMI	Read	370631000006117

Code	Term	Group	ParentGroup	CodingSystem	medcodeid
E1144	Bipolar affect disord, currently manic,severe with psychosis	BPD	SMI	Read	513701000006116
1S42	Manic mood	BPD	SMI	Read	2157096015
E1072	Chronic schizo-affective schizophrenia	SCH	SMI	Read	556631000006116
E1070	Unspecified schizo-affective schizophrenia	SCH	SMI	Read	78531000006116
Eu315	[X]Bipolar affect dis cur epi severe depres with psyc symp	BPD	SMI	Read	367061000006114
Eu220-3	[X]Paraphrenia - late	PSY	SMI	Read	418251000006110
Eu31y-2	[X]Recurrent manic episodes	BPD	SMI	Read	424661000006111
Eu202-2	[X]Schizophrenic catalepsy	SCH	SMI	Read	425641000006111
Eu20y-3	[X]Schizophrenifrm psychos NOS	SCH	SMI	Read	425711000006116
Eu300	[X]Hypomania	BPD	SMI	Read	389401000006111
Eu250-1	[X]Schizoaffective psychosis, manic type	PSY	SMI	Read	425571000006119
Eu21-7	[X]Pseudopsychopathic schizophrenia	PSY	SMI	Read	423741000006116
Eu251-2	[X]Schizophreniform psychosis, depressive type	PSY	SMI	Read	425691000006119
Eu205	[X]Residual schizophrenia	SCH	SMI	Read	424841000006113
Eu202-3	[X]Schizophrenic catatonia	SCH	SMI	Read	425651000006113
Eu22	[X]Persistent delusional disorders	PSY	SMI	Read	419861000006117
Eu25z-1	[X]Schizoaffective psychosis NOS	PSY	SMI	Read	425551000006112
Eu251-1	[X]Schizoaffective psychosis, depressive type	PSY	SMI	Read	425561000006114
Eu233-2	[X]Psychogenic paranoid psychosis	PSY	SMI	Read	424071000006112
E13z	Nonorganic psychosis NOS	PSY	SMI	Read	223611000000117
Eu200	[X]Paranoid schizophrenia	SCH	SMI	Read	418221000006118
Eu30	[X]Manic episode	BPD	SMI	Read	396741000006113
Eu3y0-1	[X]Mixed affective episode	BPD	SMI	Read	398541000006116
Eu30z-1	[X]Mania NOS	BPD	SMI	Read	396691000006111
E101	Hebephrenic schizophrenia	SCH	SMI	Read	819351000006115
Eu21-6	[X]Pseudoneurotic schizophrenia	PSY	SMI	Read	423731000006114
Eu232-4	[X]Schizophrenic reaction	SCH	SMI	Read	425671000006115

Code	Term	Group	ParentGroup	CodingSystem	medcodeid
Eu220-4	[X]Sensitiver Beziehungswahn	PSY	SMI	Read	425921000006114
Eu311	[X]Bipolar affect disorder cur epi manic wout psychotic symp	BPD	SMI	Read	367081000006116
E107-99	Acute schizo affective psychosis	PSY	SMI	Read	882301000006112
Eu24	[X]Induced delusional disorder	PSY	SMI	Read	389641000006110
E11-99	Manic-depressive psychoses	BPD	SMI	Read	882311000006110
Eu24-3	[X]Induced psychotic disorder	PSY	SMI	Read	389661000006114
E11	Affective psychoses	BPD	SMI	Read	473201000006114
ZV111-1	[V]Personal history of manic-depressive psychosis	BPD	SMI	Read	345141000006111
Eu310	[X]Bipolar affective disorder, current episode hypomanic	BPD	SMI	Read	367111000006110
Eu231	[X]Acute polymorphic psychot disord with symp of schizophren	PSY	SMI	Read	362381000006115
Eu230	[X]Acute polymorphic psychot disord without symp of schizoph	PSY	SMI	Read	362391000006117
Eu221	[X]Delusional misidentification syndrome	PSY	SMI	Read	914471000006113
Eu332-3	[X]Manic-depress psychosis,depressd,no psychotic symptoms	BPD	SMI	Read	396771000006117
Eu302-3	[X]Manic stupor	BPD	SMI	Read	396761000006112
E1146	Bipolar affective disorder, currently manic, full remission	BPD	SMI	Read	513811000006110
Eu20	[X]Schizophrenia	SCH	SMI	Read	425601000006114
Eu21-8	[X]Schizotypal personality disorder	PSY	SMI	Read	425731000006110
Eu319-1	[X]Bipolar II disorder	BPD	SMI	Read	1785871000006117
Eu250	[X]Schizoaffective disorder, manic type	PSY	SMI	Read	425511000006111
Eu223	[X]Paranoid state in remission	PSY	SMI	Read	1667581000000114
Eu23z-2	[X]Reactive psychosis	PSY	SMI	Read	424511000006112
Eu2z-1	[X]Psychosis NOS	PSY	SMI	Read	424231000006116
Eu250-2	[X]Schizophreniform psychosis, manic type	PSY	SMI	Read	425701000006119
Eu318	[X]Bipolar affective disorder type I	BPD	SMI	Read	1785851000006110

Code	Term	Group	ParentGroup	CodingSystem	medcodeid
E1074	Acute exacerbation of chronic schizo-affective schizophrenia	SCH	SMI	Read	456731000006115
EMISICD10|F2098	Schizophrenia, unspecified, other	SCH	SMI	EMIS	1976961000006110
E1165	Mixed bipolar affective disorder, partial/unspec remission	BPD	SMI	Read	701051000006118
^ESCTBO352777	BouffÃ©e dÃ©lirante	PSY	SMI	EMIS	3527771000006116
^ESCTBR258680	Brief psychotic disorder	PSY	SMI	EMIS	2586801000006115
EMISICD10|F2018	Hebephrenic schizophrenia, other	SCH	SMI	EMIS	1976891000006110
^ESCTSU272626	Subchronic disorganised schizophrenia with acute exacerbations	SCH	SMI	EMIS	2726261000006113
^ESCTFO350478	Folie E deux	PSY	SMI	EMIS	3504781000006118
^ESCTIN350475	Induced psychosis	PSY	SMI	EMIS	3504751000006114
^ESCTDI300560	Disorganized schizophrenia in remission	SCH	SMI	EMIS	3005601000006118
^ESCTSH350474	Shared psychotic disorder	PSY	SMI	EMIS	3504741000006112
^ESCTSE349764	Severe bipolar I disorder, most recent episode depressed without psychotic features	BPD	SMI	EMIS	3497641000006117
^ESCTSU293896	Subchronic disorganized schizophrenia	SCH	SMI	EMIS	2938961000006110
EMISCDE13	Delusions	PSY	SMI	EMIS	981141000006111
^ESCTBI476731	Bipolar affective disorder, current episode mixed	BPD	SMI	EMIS	4767311000006117
^ESCTDI306812	Disorganised schizophrenia	SCH	SMI	EMIS	3068121000006112
E10-99	Schizophrenic psychoses	SCH	SMI	Read	882291000006111
^ESCTSC524896	Schizophrenic prodrome	SCH	SMI	EMIS	5248961000006113
^ESCTDI300561	Disorganized schizophrenia, in remission	SCH	SMI	EMIS	3005611000006115
^ESCTIN311671	Involutional paraphrenia	PSY	SMI	EMIS	3116711000006110
^ESCTDE564562	De Clerambaults syndrome	PSY	SMI	EMIS	5645621000006116
^ESCTAC476391	Acute exacerbation of subchronic schizoaffective schizophrenia	SCH	SMI	EMIS	4763911000006114
^ESCTCH476375	Chronic catatonic schizophrenia with acute exacerbation	SCH	SMI	EMIS	4763751000006112
^ESCTSC476369	Schizophrenia, catatonic	SCH	SMI	EMIS	4763691000006113

Code	Term	Group	ParentGroup	CodingSystem	medcodeid
^ESCTNO823254	Non-organic psychosis in remission	PSY	SMI	EMIS	8232541000006114
^ESCTCH270556	Chronic disorganised schizophrenia	SCH	SMI	EMIS	2705561000006112
^ESCTCH270557	Chronic disorganized schizophrenia	SCH	SMI	EMIS	2705571000006117
^ESCTSE250101	Severe bipolar I disorder, most recent episode manic, without psychotic features	BPD	SMI	EMIS	2501011000006119
^ESCTMI823516	Mixed bipolar affective disorder, in partial remission	BPD	SMI	EMIS	8235161000006110
^ESCTSC257794	Schizophrenia, in remission	SCH	SMI	EMIS	2577941000006118
^ESCTIN350479	Induced paranoid disorder	PSY	SMI	EMIS	3504791000006115
^ESCTSU476389	Subchronic schizoaffective schizophrenia	SCH	SMI	EMIS	4763891000006112
^ESCTSU293895	Subchronic disorganised schizophrenia	SCH	SMI	EMIS	2938951000006113
^ESCTAC476364	Acute exacerbation of chronic disorganised schizophrenia	SCH	SMI	EMIS	4763641000006116
^ESCTSC387861	Schizophreniform psychosis, depressive type	SCH	SMI	EMIS	3878611000006116
^ESCTSU419615	Subchronic schizophrenia with acute exacerbations	SCH	SMI	EMIS	4196151000006111
^ESCTMI551797	Mixed schizophrenic and affective psychosis	PSY	SMI	EMIS	5517971000006118
^ESCTSC552457	Schizophreniform psychosis, manic type	PSY	SMI	EMIS	5524571000006112
^ESCTSC476370	Schizophrenic flexibilatis cerea	SCH	SMI	EMIS	4763701000006113
^ESCTSC419617	Schizophrenia, catatonic, in remission	SCH	SMI	EMIS	4196171000006118
^ESCTBI388629	Bipolar disorder, in remission	BPD	SMI	EMIS	3886291000006119
^ESCTPS362734	Psychosis	PSY	SMI	EMIS	3627341000006115
^ESCTBI635757	Bipolar 1 disorder	BPD	SMI	EMIS	6357571000006119
^ESCTMD271798	MDI - Manic-depressive illness	BPD	SMI	EMIS	2717981000006118
^ESCTRE291636	Restzustand	SCH	SMI	EMIS	2916361000006118
^ESCTSC300026	Schizoptypal disorder	SCH	SMI	EMIS	3000261000006112
^ESCTDI306813	Disorganized schizophrenia	SCH	SMI	EMIS	3068131000006110
^ESCTAC476366	Acute exacerbation of chronic disorganized schizophrenia	SCH	SMI	EMIS	4763661000006117

Code	Term	Group	ParentGroup	CodingSystem	medcodeid
^ESCTBI352868	Bipolar I disorder, most recent episode manic, in partial remission	BPD	SMI	EMIS	3528681000006111
146H	H/O: psychosis	PSY	SMI	Read	300411000000110
Eu23z-1	[X]Brief reactive psychosis NOS	PSY	SMI	Read	367991000006119
E1143	Bipolar affect disord, currently manic, severe, no psychosis	BPD	SMI	Read	513691000006116
Eu252	[X]Schizoaffective disorder, mixed type	PSY	SMI	Read	425521000006115
Eu22y	[X]Other persistent delusional disorders	PSY	SMI	Read	401857019
Eu23	[X]Acute and transient psychotic disorders	PSY	SMI	Read	362271000006110
E1156	Bipolar affective disorder, now depressed, in full remission	BPD	SMI	Read	513861000006113
E1002	Chronic schizophrenic	SCH	SMI	Read	1234861017
Eu25	[X]Schizoaffective disorders	PSY	SMI	Read	425541000006110
Eu22y-3	[X]Paranoia querulans	PSY	SMI	Read	418181000006110
E1073	Acute exacerbation subchronic schizo-affective schizophrenia	SCH	SMI	Read	456801000006115
E1025	Catatonic schizophrenia in remission	SCH	SMI	Read	178723016
E1030	Unspecified paranoid schizophrenia	SCH	SMI	Read	294754019
E1	Non-organic psychoses	PSY	SMI	Read	294724012
EMISQPA1	Paranoid	PSY	SMI	EMIS	851701000006114
E133	Acute paranoid reaction	PSY	SMI	Read	401770015
E111z	Recurrent manic episode NOS	BPD	SMI	Read	294818012
Eu25y	[X]Other schizoaffective disorders	PSY	SMI	Read	296095012
Eu231-2	[X]Cycloid psychosis with symptoms of schizophrenia	PSY	SMI	Read	376281000006116
Eu22y-2	[X]Involutional paranoid state	PSY	SMI	Read	394461000006110
E12yz	Other paranoid states NOS	PSY	SMI	Read	294912012
EGTON118	Obsessional compulsive psychosis	PSY	SMI	EMIS	853201000006116
E1y	Other specified non-organic psychoses	PSY	SMI	Read	294949018

Code	Term	Group	ParentGroup	CodingSystem	medcodeid
Eu301	[X]Mania without psychotic symptoms	BPD	SMI	Read	296110017
Eu252-1	[X]Cyclic schizophrenia	PSY	SMI	Read	376251000006112
E11y0	Unspecified manic-depressive psychoses	BPD	SMI	Read	294892012
E106	Residual schizophrenia	SCH	SMI	Read	43595011
Eu30y	[X]Other manic episodes	BPD	SMI	Read	296118012
^ESCTBI385440	Bipolar 2 disorder	BPD	SMI	EMIS	3854401000006113
E1164	Mixed bipolar affective disorder, severe, with psychosis	BPD	SMI	Read	294874016
^ESCTSY350476	Symbiotic psychosis	PSY	SMI	EMIS	3504761000006111
EMISICD10|F2054	Residual schizophrenia, incomplete remission	SCH	SMI	EMIS	1975441000006110
Eu31-2	[X]Manic-depressive psychosis	BPD	SMI	Read	396801000006115
^ESCTAC769626	Acute polymorphic psychotic disorder with symptoms of schizophrenia	PSY	SMI	EMIS	7696261000006113
Eu31-1	[X]Manic-depressive illness	BPD	SMI	Read	396791000006116
Eu206	[X]Simple schizophrenia	SCH	SMI	Read	426881000006111
Eu313	[X]Bipolar affect disorder cur epi mild or moderate depressn	BPD	SMI	Read	367091000006118
Eu221-1	[X]Capgras syndrome	PSY	SMI	Read	914461000006118
Eu3z-1	[X]Affective psychosis NOS	BPD	SMI	Read	362781000006116
^ESCTSC476395	Schizophrenia, schizoaffective, in remission	SCH	SMI	EMIS	4763951000006110
Eu302-2	[X]Mania with mood-incongruent psychotic symptoms	BPD	SMI	Read	396711000006114
E1153	Bipolar affect disord, now depressed, severe, no psychosis	BPD	SMI	Read	513731000006112
^ESCTPA355573	Paraphrenic schizophrenia	SCH	SMI	EMIS	3555731000006114
146D	H/O: manic depressive disorder	BPD	SMI	Read	1780205015
E107	Schizo-affective schizophrenia	SCH	SMI	Read	155141000006116
Eu252-2	[X]Mixed schizophrenic and affective psychosis	PSY	SMI	Read	398631000006113
Eu24-2	[X]Induced paranoid disorder	PSY	SMI	Read	389651000006112

Code	Term	Group	ParentGroup	CodingSystem	medcodeid
Eu21-4	[X]Prepsychotic schizophrenia	PSY	SMI	Read	423271000006116
E2122	Schizotypal personality	PSY	SMI	Read	155281000006119
E122	Paraphrenia	PSY	SMI	Read	44335019
Eu2y	[X]Other nonorganic psychotic disorders	PSY	SMI	Read	412201000006113
E10y0	Atypical schizophrenia	SCH	SMI	Read	1219653018
Eu201-1	[X]Disorganised schizophrenia	SCH	SMI	Read	378051000006119
^ESCTPO502390	Post-schizophrenic depression	SCH	SMI	EMIS	5023901000006110
Eu202-1	[X]Catatonic stupor	SCH	SMI	Read	370461000006112
E115-1	Manic-depressive - now depressed	BPD	SMI	Read	294860011
E12y0	Paranoia querulans	PSY	SMI	Read	294911017
E13z-1	Psychotic episode NOS	PSY	SMI	Read	346895011
E110-99	Mania/hypomania	BPD	SMI	Read	882321000006119
E1101	Single manic episode, mild	BPD	SMI	Read	294803011
E1034	Acute exacerbation of chronic paranoid schizophrenia	SCH	SMI	Read	294758016
Eu205-1	[X]Chronic undifferentiated schizophrenia	SCH	SMI	Read	371031000006115
Eu251	[X]Schizoaffective disorder, depressive type	PSY	SMI	Read	425501000006113
E106-1	Restzustand - schizophrenia	SCH	SMI	Read	169061000006112
Eu31-3	[X]Manic-depressive reaction	BPD	SMI	Read	396071000006119
E1150	Bipolar affective disorder, currently depressed, unspecified	BPD	SMI	Read	294861010
E1050	Unspecified latent schizophrenia	PSY	SMI	Read	294765012
^ESCTSC476394	Schizoaffective schizophrenia, in remission	SCH	SMI	EMIS	4763941000006113
Eu317	[X]Bipolar affective disorder, currently in remission	BPD	SMI	Read	296130018
^ESCTFO350473	Folie Ã deux	PSY	SMI	EMIS	3504731000006119
^ESCTSU476377	Subchronic paranoid schizophrenia with acute exacerbation	SCH	SMI	EMIS	4763771000006119
Eu314	[X]Bipol aff disord, curr epis sev depress, no psychot symp	BPD	SMI	Read	367051000006112

Code	Term	Group	ParentGroup	CodingSystem	medcodeid
E1004	Acute exacerbation of chronic schizophrenia	SCH	SMI	Read	294731011
E110z	Manic disorder, single episode NOS	BPD	SMI	Read	294809010
E12-99	Paranoia	PSY	SMI	Read	882331000006116
Eu232-2	[X]Brief schizophrenifrm psych	PSY	SMI	Read	368011000006110
E13	Other nonorganic psychoses	PSY	SMI	Read	25461000006115
^ESCTAC476392	Acute exacerbation of chronic schizoaffective schizophrenia	SCH	SMI	EMIS	4763921000006118
E11y	Other and unspecified manic-depressive psychoses	BPD	SMI	Read	294891017
^ESCTMA502396	Manic	BPD	SMI	EMIS	5023961000006111
E104-1	Oneirophrenia	PSY	SMI	Read	450785011
Eu302	[X]Mania with psychotic symptoms	BPD	SMI	Read	401863011
E1154	Bipolar affect disord, now depressed, severe with psychosis	BPD	SMI	Read	513721000006114
E13y0	Psychogenic stupor	PSY	SMI	Read	294927012
Eu200-1	[X]Paraphrenic schizophrenia	SCH	SMI	Read	418261000006112
Eu205-2	[X]Restzustand schizophrenic	SCH	SMI	Read	424931000006113
E1116	Recurrent manic episodes, in full remission	BPD	SMI	Read	294817019
Eu201	[X]Hebephrenic schizophrenia	SCH	SMI	Read	388741000006110
E107-1	Cyclic schizophrenia	PSY	SMI	Read	294773015
E11zz	Other affective psychosis NOS	BPD	SMI	Read	294902017
^ESCTIN350472	Induced psychotic disorder	PSY	SMI	EMIS	3504721000006117
Eu21-1	[X]Latent schizophrenic reaction	PSY	SMI	Read	395031000006114
Eu319	[X]Bipolar affective disorder type II	BPD	SMI	Read	1785861000006112
Eu0z-2	[X]Symptomatic psychosis NOS	PSY	SMI	Read	428451000006119
E1145	Bipolar affect disord,currently manic, part/unspec remission	BPD	SMI	Read	513741000006119
E1051	Subchronic latent schizophrenia	PSY	SMI	Read	294766013

Code	Term	Group	ParentGroup	CodingSystem	medcodeid
^ESCTSU272627	Subchronic disorganized schizophrenia with acute exacerbations	SCH	SMI	EMIS	2726271000006118
^ESCTPA352741	Paranoid schizophrenia, in remission	SCH	SMI	EMIS	3527411000006119
Eu21-2	[X]Borderline schizophrenia	SCH	SMI	Read	367951000006113
^ESCTDI300559	Disorganised schizophrenia in remission	SCH	SMI	EMIS	3005591000006114
^ESCTSU476373	Subchronic catatonic schizophrenia with acute exacerbation	SCH	SMI	EMIS	4763731000006117
^ESCTLA476386	Latent schizophrenia, in remission	SCH	SMI	EMIS	4763861000006116
Eu20y-2	[X]Schizophreniform disord NOS	SCH	SMI	Read	425681000006117
E13-1	Reactive psychoses	PSY	SMI	Read	346896012
^ESCTBI276168	Bipolar I disorder, most recent episode mixed	BPD	SMI	EMIS	2761681000006119
E121	Chronic paranoid psychosis	PSY	SMI	Read	401768012
Eu23z	[X]Acute and transient psychotic disorder, unspecified	PSY	SMI	Read	401860014
Eu22y-1	[X]Delusional dysmorphophobia	PSY	SMI	Read	376501000006110
^ESCTRE502339	Reactive psychosis	PSY	SMI	EMIS	5023391000006116
EMISICD10|F2035	Undifferentiated schizophrenia, complete remission	SCH	SMI	EMIS	1975081000006110
^ESCTBI300667	Bipolar I disorder, most recent episode hypomanic	BPD	SMI	EMIS	3006671000006118
Eu26	[X]Nonorganic psychosis in remission	PSY	SMI	Read	1667591000000111
EMISQHY1	Hypomanic	BPD	SMI	EMIS	851691000006114
Eu202-4	[X]Schizophrenic flexibilatis cerea	SCH	SMI	Read	294743012
E123	Shared paranoid disorder	PSY	SMI	Read	294909014
Eu528	[X]Erotomania	PSY	SMI	Read	2532965018
Eu203-1	[X]Atypical schizophrenia	SCH	SMI	Read	366571000006114
Eu2y-1	[X]Chronic hallucinatory psychosis	PSY	SMI	Read	370981000006112
E103	Paranoid schizophrenia	SCH	SMI	Read	107878010
E1141	Bipolar affective disorder, currently manic, mild	BPD	SMI	Read	294849019

Code	Term	Group	ParentGroup	CodingSystem	medcodeid
E1033	Acute exacerbation of subchronic paranoid schizophrenia	SCH	SMI	Read	294757014
E1114	Recurrent manic episodes, severe, with psychosis	BPD	SMI	Read	294815010
^ESCTCH476379	Chronic paranoid schizophrenia with acute exacerbation	SCH	SMI	EMIS	4763791000006118
Eu21-5	[X]Prodromal schizophrenia	PSY	SMI	Read	423471000006117
Eu302-1	[X]Mania with mood-congruent psychotic symptoms	BPD	SMI	Read	396701000006111
E1053	Acute exacerbation of subchronic latent schizophrenia	PSY	SMI	Read	294768014
Eu2	[X]Schizophrenia, schizotypal and delusional disorders	PSY	SMI	Read	296022017
E10y1	Coenesthopathic schizophrenia	SCH	SMI	Read	294787015
EMISICD10|F2041	Post-schizophrenic depression, episodic with progressive deficit	SCH	SMI	EMIS	1975211000006110
E110-1	Hypomanic psychoses	BPD	SMI	Read	789221000006116
Eu220-2	[X]Paranoid state	PSY	SMI	Read	418231000006115
Eu204	[X]Post-schizophrenic depression	SCH	SMI	Read	423041000006119
E1075	Schizo-affective schizophrenia in remission	SCH	SMI	Read	155151000006119
E115	Bipolar affective disorder, currently depressed	BPD	SMI	Read	513751000006117
E1035	Paranoid schizophrenia in remission	SCH	SMI	Read	105029017
E1111	Recurrent manic episodes, mild	BPD	SMI	Read	294812013
E1112	Recurrent manic episodes, moderate	BPD	SMI	Read	294813015
E13yz	Other reactive psychoses NOS	PSY	SMI	Read	294929010
^ESCTCH386266	Chronic schizophrenia	SCH	SMI	EMIS	3862661000006112
Eu232-3	[X]Oneirophrenia	PSY	SMI	Read	403611000006116
E1174	Unspecified bipolar affective disorder,severe with psychosis	BPD	SMI	Read	82171000006116
E10-98	Schizophrenic psychoses NOS	SCH	SMI	Read	882281000006113
Eu232-1	[X]Brief schizophreniform disorder	PSY	SMI	Read	368001000006112

Code	Term	Group	ParentGroup	CodingSystem	medcodeid
1464	H/O: schizophrenia	SCH	SMI	Read	251628010
Eu333-2	[X]Manic-depress psychosis,depressed type+psychotic symptoms	BPD	SMI	Read	396781000006119
Eu230-2	[X]Cycloid psychosis	PSY	SMI	Read	376271000006119
E1010	Unspecified hebephrenic schizophrenia	SCH	SMI	Read	294735019
E1071	Subchronic schizo-affective schizophrenia	SCH	SMI	Read	123611000006110
Eu220	[X]Delusional disorder	PSY	SMI	Read	215841000000114
E103z	Paranoid schizophrenia NOS	SCH	SMI	Read	294760019
E1003	Acute exacerbation of subchronic schizophrenia	SCH	SMI	Read	294730012
E1020	Unspecified catatonic schizophrenia	SCH	SMI	Read	294744018
E1163	Mixed bipolar affective disorder, severe, without psychosis	BPD	SMI	Read	701071000006111
EMISICD10|F2065	Simple schizophrenia, complete remission	SCH	SMI	EMIS	1975601000006110
E117z	Unspecified bipolar affective disorder, NOS	BPD	SMI	Read	294888017
Eu203	[X]Undifferentiated schizophrenia	SCH	SMI	Read	296031017
E1011	Subchronic hebephrenic schizophrenia	SCH	SMI	Read	294736018
E11-3	Manic psychoses	PSY	SMI	Read	223601000000119
Eu30-1	[X]Bipolar disorder, single manic episode	BPD	SMI	Read	367151000006111
E11-1	Bipolar psychoses	BPD	SMI	Read	513871000006118
E1105	Single manic episode in partial or unspecified remission	BPD	SMI	Read	294807012
E1152	Bipolar affective disorder, currently depressed, moderate	BPD	SMI	Read	294863013
^ESCTBI317407	Bipolar disorder, full remission	BPD	SMI	EMIS	3174071000006116
^ESCT1248045	[X]Schizophrenia, schizotypal and delusional disorders	SCH	SMI	EMIS	12480451000006112
Eu20y	[X]Other schizophrenia	SCH	SMI	Read	401855010
E1173	Unspecified bipolar affective disorder, severe, no psychosis	BPD	SMI	Read	82151000006114
4926007	Schizophrenia in remission	SCH	SMI	SNOMED	9225016

Code	Term	Group	ParentGroup	CodingSystem	medcodeid
31658008	Chronic paranoid schizophrenia	SCH	SMI	SNOMED	52897013
68995007	Chronic catatonic schizophrenia	SCH	SMI	SNOMED	114616017
16990005	Subchronic schizophrenia	SCH	SMI	SNOMED	28758018
42868002	Subchronic catatonic schizophrenia	SCH	SMI	SNOMED	71539017
5464005	Brief reactive psychosis	PSY	SMI	SNOMED	10122017
79866005	Subchronic paranoid schizophrenia	SCH	SMI	SNOMED	132503015
14291003	Acute exacerbation of subchronic hebephrenic schizophrenia	SCH	SMI	SNOMED	294738017
191590005	Recurrent manic episodes	BPD	SMI	SNOMED	294810017
191627008	Bipolar affective disorder, currently depressed, NOS	BPD	SMI	SNOMED	294868016
191668004	Simple paranoid state	PSY	SMI	SNOMED	294904016
231437006	Other reactive psychoses	PSY	SMI	SNOMED	294926015
191539009	Acute exacerbation of chronic hebephrenic schizophrenia	SCH	SMI	SNOMED	294739013
191542003	Catatonic schizophrenia	SCH	SMI	SNOMED	294742019
191547009	Acute exacerbation of subchronic catatonic schizophrenia	SCH	SMI	SNOMED	294750011
58214004	Other schizophrenia NOS	SCH	SMI	SNOMED	294789017
191618007	Bipolar affective disorder, currently manic, NOS	BPD	SMI	SNOMED	294858014
191636007	Mixed bipolar affective disorder	BPD	SMI	SNOMED	294869012
191670008	Folie a deux	PSY	SMI	SNOMED	294908018
191527001	Simple schizophrenia NOS	SCH	SMI	SNOMED	294734015
191588009	Single manic episode in full remission	BPD	SMI	SNOMED	294808019
191667009	Paranoid psychosis NOS	PSY	SMI	SNOMED	294913019
191680007	Psychogenic paranoid psychosis	PSY	SMI	SNOMED	294924017
191527001	Schizophrenia simplex	SCH	SMI	SNOMED	294726014
764641000000104	Single manic episode, severe without mention of psychosis	BPD	SMI	SNOMED	294805016

Code	Term	Group	ParentGroup	CodingSystem	medcodeid
191586008	Single manic episode, severe, with psychosis	BPD	SMI	SNOMED	294806015
191621009	Bipolar affective disorder, currently manic, moderate	BPD	SMI	SNOMED	294850019
191565008	Latent schizophrenia in remission	PSY	SMI	SNOMED	294770017
191638008	Mixed bipolar affective disorder, mild	BPD	SMI	SNOMED	294871012
35252006	Hebephrenic schizophrenia NOS	SCH	SMI	SNOMED	294741014
191548004	Acute exacerbation of chronic catatonic schizophrenia	SCH	SMI	SNOMED	294751010
191584006	Single manic episode, moderate	BPD	SMI	SNOMED	294804017
191667009	Other paranoid states	PSY	SMI	SNOMED	294910016
268619003	Single manic episode, unspecified	BPD	SMI	SNOMED	294802018
191636007	Mixed bipolar affective disorder, unspecified	BPD	SMI	SNOMED	294870013
191639000	Mixed bipolar affective disorder, moderate	BPD	SMI	SNOMED	294872017
231489001	[X]Other acute and transient psychotic disorders	PSY	SMI	SNOMED	296083012
191618007	Manic-depressive - now manic	BPD	SMI	SNOMED	294847017
191618007	Bipolar affective disorder, currently manic, unspecified	BPD	SMI	SNOMED	294848010
13746004	Other and unspecified manic-depressive psychoses NOS	BPD	SMI	SNOMED	294896010
231487004	[X]Persistent delusional disorder, unspecified	PSY	SMI	SNOMED	296066015
191577003	Cenesthopathic schizophrenia	SCH	SMI	SNOMED	294788013
79584002	Unspecified bipolar affective disorder, moderate	BPD	SMI	SNOMED	294883014
191590005	Recurrent manic episodes, unspecified	BPD	SMI	SNOMED	294811018
191629006	Bipolar affective disorder, currently depressed, mild	BPD	SMI	SNOMED	294862015
16506000	Other mixed manic-depressive psychoses	BPD	SMI	SNOMED	294895014
58214004	[X]Schizophrenia, unspecified	SCH	SMI	SNOMED	296040018
191564007	Acute exacerbation of chronic latent schizophrenia	PSY	SMI	SNOMED	294769018
191562006	Chronic latent schizophrenia	PSY	SMI	SNOMED	294767016

Code	Term	Group	ParentGroup	CodingSystem	medcodeid
41836007	Unspecified bipolar affective disorder, in full remission	BPD	SMI	SNOMED	294887010
191658009	Atypical manic disorder	BPD	SMI	SNOMED	294893019
68890003	[X]Schizoaffective disorder, unspecified	PSY	SMI	SNOMED	296096013
191542003	Catatonic schizophrenia NOS	SCH	SMI	SNOMED	294753013
191559008	Latent schizophrenia	PSY	SMI	SNOMED	294764011
191643001	Mixed bipolar affective disorder, in full remission	BPD	SMI	SNOMED	294876019
13746004	Unspecified bipolar affective disorder	BPD	SMI	SNOMED	294880012
13313007	Unspecified bipolar affective disorder, mild	BPD	SMI	SNOMED	294882016
441704009	Unspecified affective psychoses NOS	BPD	SMI	SNOMED	294898011
191526005	Schizophrenic disorders	SCH	SMI	SNOMED	294725013
191527001	Simple schizophrenia	SCH	SMI	SNOMED	294727017
12939007	Chronic hebephrenic schizophrenia	SCH	SMI	SNOMED	294737010
191636007	Mixed bipolar affective disorder, NOS	BPD	SMI	SNOMED	294877011
441704009	Other and unspecified affective psychoses	BPD	SMI	SNOMED	294897018
13746004	[X]Bipolar affective disorder, unspecified	BPD	SMI	SNOMED	296135011
58214004	Unspecified schizophrenia	SCH	SMI	SNOMED	294728010
31373002	Hebephrenic schizophrenia in remission	SCH	SMI	SNOMED	294740010
58214004	Schizophrenia NOS	SCH	SMI	SNOMED	294790014
13746004	Unspecified bipolar affective disorder, unspecified	BPD	SMI	SNOMED	294881011
191677006	Acute hysterical psychosis	PSY	SMI	SNOMED	294919015
191559008	Latent schizophrenia NOS	PSY	SMI	SNOMED	294771018
268622001	Sanders disease	PSY	SMI	SNOMED	161371000006118
231489001	[X]Other acute predominantly delusional psychotic disorders	PSY	SMI	SNOMED	401859016
13746004	[X]Other bipolar affective disorders	BPD	SMI	SNOMED	401865016
764671000000105	Recurrent manic episodes, partial or unspecified remission	BPD	SMI	SNOMED	182861000006110
268619003	[X]Manic episode, unspecified	BPD	SMI	SNOMED	401864017

Code	Term	Group	ParentGroup	CodingSystem	medcodeid
417233008	[X]Paranoia	PSY	SMI	SNOMED	215871000000115
191567000	Schizo-affective schizophrenia NOS	SCH	SMI	SNOMED	155161000006117
191667009	[X]Paranoid psychosis	PSY	SMI	SNOMED	215851000000112
12200008	Poor insight into psychotic condition	PSY	SMI	SNOMED	215691000006112
31027006	[X]Schizotypal disorder	PSY	SMI	SNOMED	401856011
268619003	Manic disorder, single episode	BPD	SMI	SNOMED	401765010
764621000000106	Recurrent manic episodes, severe without mention psychosis	BPD	SMI	SNOMED	182871000006115
58214004	Other schizophrenia	SCH	SMI	SNOMED	401764014
191525009	Non-organic psychosis NOS	PSY	SMI	SNOMED	401771016
161464003	[V]Personal history of schizophrenia	SCH	SMI	SNOMED	460273017
278853003	[X]Acute schizophrenia-like psychotic disorder	PSY	SMI	SNOMED	362421000006113
191525009	[X]Unspecified nonorganic psychosis	PSY	SMI	SNOMED	401862018
191667009	Paranoid states	PSY	SMI	SNOMED	243361000006117
268617001	Acute schizophrenic episode	SCH	SMI	SNOMED	401763015
5703000	Unspecified bipolar affect disord, partial/unspec remission	BPD	SMI	SNOMED	82091000006116
429124005	[V]Personal history of manic-depressive psychosis	BPD	SMI	SNOMED	1227584015
63204009	[X]Bouffee delirante	PSY	SMI	SNOMED	367961000006110
712850003	[X]Bouffee delirante with symptoms of schizophrenia	PSY	SMI	SNOMED	367971000006115
13746004	[X]Bipolar affective disorder	BPD	SMI	SNOMED	367101000006112
192362008	[X]Bipolar affective disorder, current episode mixed	BPD	SMI	SNOMED	367121000006119
191623007	[X]Bipolar affect disorder cur epi manic with psychotic symp	BPD	SMI	SNOMED	367071000006119
191542003	[X]Catatonic schizophrenia	SCH	SMI	SNOMED	370451000006110
191559008	[X]Latent schizophrenia	PSY	SMI	SNOMED	395021000006111
191618007	Bipolar affective disorder, currently manic	BPD	SMI	SNOMED	513801000006112

Code	Term	Group	ParentGroup	CodingSystem	medcodeid
191670008	[X]Folie a deux	PSY	SMI	SNOMED	387071000006117
49512000	Bipolar affect disord, now depressed, part/unspec remission	BPD	SMI	SNOMED	513711000006118
63204009	Bouffee delirante	PSY	SMI	SNOMED	523481000006114
83225003	[X]Bipolar II disorder	BPD	SMI	SNOMED	367161000006113
58214004	[X]Cenesthopathic schizophrenia	SCH	SMI	SNOMED	370631000006117
191623007	Bipolar affect disord, currently manic,severe with psychosis	BPD	SMI	SNOMED	513701000006116
405273008	Manic mood	BPD	SMI	SNOMED	2157096015
191570001	Chronic schizo-affective schizophrenia	SCH	SMI	SNOMED	556631000006116
191567000	Unspecified schizo-affective schizophrenia	SCH	SMI	SNOMED	78531000006116
765176007	[X]Bipolar affect dis cur epi severe depres with psyc symp	BPD	SMI	SNOMED	367061000006114
38295006	[X]Paraphrenia - late	PSY	SMI	SNOMED	418251000006110
191590005	[X]Recurrent manic episodes	BPD	SMI	SNOMED	424661000006111
191542003	[X]Schizophrenic catalepsy	SCH	SMI	SNOMED	425641000006111
58214004	[X]Schizophrenifrm psychos NOS	SCH	SMI	SNOMED	425711000006116
231496004	[X]Hypomania	BPD	SMI	SNOMED	389401000006111
271428004	[X]Schizoaffective psychosis, manic type	PSY	SMI	SNOMED	425571000006119
31027006	[X]Pseudopsychopathic schizophrenia	PSY	SMI	SNOMED	423741000006116
84760002	[X]Schizophreniform psychosis, depressive type	PSY	SMI	SNOMED	425691000006119
26025008	[X]Residual schizophrenia	SCH	SMI	SNOMED	424841000006113
191542003	[X]Schizophrenic catatonia	SCH	SMI	SNOMED	425651000006113
231487004	[X]Persistent delusional disorders	PSY	SMI	SNOMED	419861000006117
68890003	[X]Schizoaffective psychosis NOS	PSY	SMI	SNOMED	425551000006112
84760002	[X]Schizoaffective psychosis, depressive type	PSY	SMI	SNOMED	425561000006114
191680007	[X]Psychogenic paranoid psychosis	PSY	SMI	SNOMED	424071000006112
191525009	Nonorganic psychosis NOS	PSY	SMI	SNOMED	223611000000117
64905009	[X]Paranoid schizophrenia	SCH	SMI	SNOMED	418221000006118

Code	Term	Group	ParentGroup	CodingSystem	medcodeid
268619003	[X]Manic episode	BPD	SMI	SNOMED	396741000006113
192362008	[X]Mixed affective episode	BPD	SMI	SNOMED	398541000006116
268619003	[X]Mania NOS	BPD	SMI	SNOMED	396691000006111
35252006	Hebephrenic schizophrenia	SCH	SMI	SNOMED	819351000006115
31027006	[X]Pseudoneurotic schizophrenia	PSY	SMI	SNOMED	423731000006114
278853003	[X]Schizophrenic reaction	SCH	SMI	SNOMED	425671000006115
41189006	[X]Sensitiver Beziehungswahn	PSY	SMI	SNOMED	425921000006114
191618007	[X]Bipolar affect disorder cur epi manic wout psychotic symp	BPD	SMI	SNOMED	367081000006116
191567000	Acute schizo affective psychosis	PSY	SMI	SNOMED	882301000006112
61831009	[X]Induced delusional disorder	PSY	SMI	SNOMED	389641000006110
13746004	Manic-depressive psychoses	BPD	SMI	SNOMED	882311000006110
61831009	[X]Induced psychotic disorder	PSY	SMI	SNOMED	389661000006114
46206005	Affective psychoses	BPD	SMI	SNOMED	473201000006114
429124005	[V]Personal history of manic-depressive psychosis	BPD	SMI	SNOMED	345141000006111
31446002	[X]Bipolar affective disorder, current episode hypomanic	BPD	SMI	SNOMED	367111000006110
712850003	[X]Acute polymorphic psychot disord with symp of schizophren	PSY	SMI	SNOMED	362381000006115
712824002	[X]Acute polymorphic psychot disord without symp of schizoph	PSY	SMI	SNOMED	362391000006117
44906001	[X]Delusional misidentification syndrome	PSY	SMI	SNOMED	914471000006113
36474008	[X]Manic-depress psychosis,depressd,no psychotic symptoms	BPD	SMI	SNOMED	396771000006117
231494001	[X]Manic stupor	BPD	SMI	SNOMED	396761000006112
191625000	Bipolar affective disorder, currently manic, full remission	BPD	SMI	SNOMED	513811000006110
58214004	[X]Schizophrenia	SCH	SMI	SNOMED	425601000006114
31027006	[X]Schizotypal personality disorder	PSY	SMI	SNOMED	425731000006110
83225003	[X]Bipolar II disorder	BPD	SMI	SNOMED	1785871000006117

Code	Term	Group	ParentGroup	CodingSystem	medcodeid
271428004	[X]Schizoaffective disorder, manic type	PSY	SMI	SNOMED	425511000006111
755301000000102	[X]Paranoid state in remission	PSY	SMI	SNOMED	1667581000000114
231437006	[X]Reactive psychosis	PSY	SMI	SNOMED	424511000006112
69322001	[X]Psychosis NOS	PSY	SMI	SNOMED	424231000006116
271428004	[X]Schizophreniform psychosis, manic type	PSY	SMI	SNOMED	425701000006119
371596008	[X]Bipolar affective disorder type I	BPD	SMI	SNOMED	1785851000006110
191572009	Acute exacerbation of chronic schizo-affective schizophrenia	SCH	SMI	SNOMED	456731000006115
1976961000006106	Schizophrenia, unspecified, other	SCH	SMI	SNOMED	1976961000006110
760721000000109	Mixed bipolar affective disorder, partial/unspec remission	BPD	SMI	SNOMED	701051000006118
63204009	BouffÃ©e dÃ©lirante	PSY	SMI	SNOMED	3527771000006116
5464005	Brief psychotic disorder	PSY	SMI	SNOMED	2586801000006115
1976891000006106	Hebephrenic schizophrenia, other	SCH	SMI	SNOMED	1976891000006110
14291003	Subchronic disorganised schizophrenia with acute exacerbations	SCH	SMI	SNOMED	2726261000006113
61831009	Folie E deux	PSY	SMI	SNOMED	3504781000006118
61831009	Induced psychosis	PSY	SMI	SNOMED	3504751000006114
31373002	Disorganized schizophrenia in remission	SCH	SMI	SNOMED	3005601000006118
61831009	Shared psychotic disorder	PSY	SMI	SNOMED	3504741000006112
61403008	Severe bipolar I disorder, most recent episode depressed without psychotic features	BPD	SMI	SNOMED	3497641000006117
27387000	Subchronic disorganized schizophrenia	SCH	SMI	SNOMED	2938961000006110
981141000006107	Delusions	PSY	SMI	SNOMED	981141000006111
192362008	Bipolar affective disorder, current episode mixed	BPD	SMI	SNOMED	4767311000006117
35252006	Disorganised schizophrenia	SCH	SMI	SNOMED	3068121000006112
191526005	Schizophrenic psychoses	SCH	SMI	SNOMED	882291000006111
247804008	Schizophrenic prodrome	SCH	SMI	SNOMED	5248961000006113
31373002	Disorganized schizophrenia, in remission	SCH	SMI	SNOMED	3005611000006115

Code	Term	Group	ParentGroup	CodingSystem	medcodeid
38295006	Involutional paraphrenia	PSY	SMI	SNOMED	3116711000006110
280949006	De Clerambaults syndrome	PSY	SMI	SNOMED	5645621000006116
191571002	Acute exacerbation of subchronic schizoaffective schizophrenia	SCH	SMI	SNOMED	4763911000006114
191548004	Chronic catatonic schizophrenia with acute exacerbation	SCH	SMI	SNOMED	4763751000006112
191542003	Schizophrenia, catatonic	SCH	SMI	SNOMED	4763691000006113
755311000000100	Non-organic psychosis in remission	PSY	SMI	SNOMED	8232541000006114
12939007	Chronic disorganised schizophrenia	SCH	SMI	SNOMED	2705561000006112
12939007	Chronic disorganized schizophrenia	SCH	SMI	SNOMED	2705571000006117
162004	Severe bipolar I disorder, most recent episode manic, without psychotic features	BPD	SMI	SNOMED	2501011000006119
760721000000109	Mixed bipolar affective disorder, in partial remission	BPD	SMI	SNOMED	8235161000006110
4926007	Schizophrenia, in remission	SCH	SMI	SNOMED	2577941000006118
61831009	Induced paranoid disorder	PSY	SMI	SNOMED	3504791000006115
191569002	Subchronic schizoaffective schizophrenia	SCH	SMI	SNOMED	4763891000006112
27387000	Subchronic disorganised schizophrenia	SCH	SMI	SNOMED	2938951000006113
191539009	Acute exacerbation of chronic disorganised schizophrenia	SCH	SMI	SNOMED	4763641000006116
84760002	Schizophreniform psychosis, depressive type	SCH	SMI	SNOMED	3878611000006116
111482003	Subchronic schizophrenia with acute exacerbations	SCH	SMI	SNOMED	4196151000006111
270901009	Mixed schizophrenic and affective psychosis	PSY	SMI	SNOMED	5517971000006118
271428004	Schizophreniform psychosis, manic type	PSY	SMI	SNOMED	5524571000006112
191542003	Schizophrenic flexibilatis cerea	SCH	SMI	SNOMED	4763701000006113
111483008	Schizophrenia, catatonic, in remission	SCH	SMI	SNOMED	4196171000006118
85248005	Bipolar disorder, in remission	BPD	SMI	SNOMED	3886291000006119
69322001	Psychosis	PSY	SMI	SNOMED	3627341000006115
371596008	Bipolar 1 disorder	BPD	SMI	SNOMED	6357571000006119

Code	Term	Group	ParentGroup	CodingSystem	medcodeid
13746004	MDI - Manic-depressive illness	BPD	SMI	SNOMED	2717981000006118
26025008	Restzustand	SCH	SMI	SNOMED	2916361000006118
31027006	Schizoptypal disorder	SCH	SMI	SNOMED	3000261000006112
35252006	Disorganized schizophrenia	SCH	SMI	SNOMED	3068131000006110
191539009	Acute exacerbation of chronic disorganized schizophrenia	SCH	SMI	SNOMED	4763661000006117
63249007	Bipolar I disorder, most recent episode manic, in partial remission	BPD	SMI	SNOMED	3528681000006111
198991000000103	H/O: psychosis	PSY	SMI	SNOMED	300411000000110
231489001	[X]Brief reactive psychosis NOS	PSY	SMI	SNOMED	367991000006119
162004	Bipolar affect disord, currently manic, severe, no psychosis	BPD	SMI	SNOMED	513691000006116
270901009	[X]Schizoaffective disorder, mixed type	PSY	SMI	SNOMED	425521000006115
231487004	[X]Other persistent delusional disorders	PSY	SMI	SNOMED	401857019
231489001	[X]Acute and transient psychotic disorders	PSY	SMI	SNOMED	362271000006110
191634005	Bipolar affective disorder, now depressed, in full remission	BPD	SMI	SNOMED	513861000006113
83746006	Chronic schizophrenic	SCH	SMI	SNOMED	1234861017
68890003	[X]Schizoaffective disorders	PSY	SMI	SNOMED	425541000006110
231487004	[X]Paranoia querulans	PSY	SMI	SNOMED	418181000006110
191571002	Acute exacerbation subchronic schizo-affective schizophrenia	SCH	SMI	SNOMED	456801000006115
111483008	Catatonic schizophrenia in remission	SCH	SMI	SNOMED	178723016
64905009	Unspecified paranoid schizophrenia	SCH	SMI	SNOMED	294754019
191525009	Non-organic psychoses	PSY	SMI	SNOMED	294724012
851701000006105	Paranoid	PSY	SMI	SNOMED	851701000006114
268624000	Acute paranoid reaction	PSY	SMI	SNOMED	401770015
191590005	Recurrent manic episode NOS	BPD	SMI	SNOMED	294818012
68890003	[X]Other schizoaffective disorders	PSY	SMI	SNOMED	296095012

Code	Term	Group	ParentGroup	CodingSystem	medcodeid
307417003	[X]Cycloid psychosis with symptoms of schizophrenia	PSY	SMI	SNOMED	376281000006116
231487004	[X]Involutional paranoid state	PSY	SMI	SNOMED	394461000006110
191667009	Other paranoid states NOS	PSY	SMI	SNOMED	294912012
853201000006100	Obsessional compulsive psychosis	PSY	SMI	SNOMED	853201000006116
191525009	Other specified non-organic psychoses	PSY	SMI	SNOMED	294949018
231494001	[X]Mania without psychotic symptoms	BPD	SMI	SNOMED	296110017
270901009	[X]Cyclic schizophrenia	PSY	SMI	SNOMED	376251000006112
13746004	Unspecified manic-depressive psychoses	BPD	SMI	SNOMED	294892012
26025008	Residual schizophrenia	SCH	SMI	SNOMED	43595011
231494001	[X]Other manic episodes	BPD	SMI	SNOMED	296118012
83225003	Bipolar 2 disorder	BPD	SMI	SNOMED	3854401000006113
191641004	Mixed bipolar affective disorder, severe, with psychosis	BPD	SMI	SNOMED	294874016
61831009	Symbiotic psychosis	PSY	SMI	SNOMED	3504761000006111
1975441000006106	Residual schizophrenia, incomplete remission	SCH	SMI	SNOMED	1975441000006110
13746004	[X]Manic-depressive psychosis	BPD	SMI	SNOMED	396801000006115
712850003	Acute polymorphic psychotic disorder with symptoms of schizophrenia	PSY	SMI	SNOMED	7696261000006113
13746004	[X]Manic-depressive illness	BPD	SMI	SNOMED	396791000006116
191527001	[X]Simple schizophrenia	SCH	SMI	SNOMED	426881000006111
191630001	[X]Bipolar affect disorder cur epi mild or moderate depressn	BPD	SMI	SNOMED	367091000006118
44906001	[X]Capgras syndrome	PSY	SMI	SNOMED	914461000006118
46206005	[X]Affective psychosis NOS	BPD	SMI	SNOMED	362781000006116
191574005	Schizophrenia, schizoaffective, in remission	SCH	SMI	SNOMED	4763951000006110
231494001	[X]Mania with mood-incongruent psychotic symptoms	BPD	SMI	SNOMED	396711000006114
61403008	Bipolar affect disord, now depressed, severe, no psychosis	BPD	SMI	SNOMED	513731000006112

Code	Term	Group	ParentGroup	CodingSystem	medcodeid
64905009	Paraphrenic schizophrenia	SCH	SMI	SNOMED	3555731000006114
400998002	H/O: manic depressive disorder	BPD	SMI	SNOMED	1780205015
191567000	Schizo-affective schizophrenia	SCH	SMI	SNOMED	155141000006116
270901009	[X]Mixed schizophrenic and affective psychosis	PSY	SMI	SNOMED	398631000006113
61831009	[X]Induced paranoid disorder	PSY	SMI	SNOMED	389651000006112
247804008	[X]Prepsychotic schizophrenia	PSY	SMI	SNOMED	423271000006116
31027006	Schizotypal personality	PSY	SMI	SNOMED	155281000006119
26472000	Paraphrenia	PSY	SMI	SNOMED	44335019
191525009	[X]Other nonorganic psychotic disorders	PSY	SMI	SNOMED	412201000006113
111484002	Atypical schizophrenia	SCH	SMI	SNOMED	1219653018
35252006	[X]Disorganised schizophrenia	SCH	SMI	SNOMED	378051000006119
231485007	Post-schizophrenic depression	SCH	SMI	SNOMED	5023901000006110
191542003	[X]Catatonic stupor	SCH	SMI	SNOMED	370461000006112
191627008	Manic-depressive - now depressed	BPD	SMI	SNOMED	294860011
191672000	Paranoia querulans	PSY	SMI	SNOMED	294911017
69322001	Psychotic episode NOS	PSY	SMI	SNOMED	346895011
268619003	Mania/hypomania	BPD	SMI	SNOMED	882321000006119
191583000	Single manic episode, mild	BPD	SMI	SNOMED	294803011
191555002	Acute exacerbation of chronic paranoid schizophrenia	SCH	SMI	SNOMED	294758016
111484002	[X]Chronic undifferentiated schizophrenia	SCH	SMI	SNOMED	371031000006115
84760002	[X]Schizoaffective disorder, depressive type	PSY	SMI	SNOMED	425501000006113
26025008	Restzustand - schizophrenia	SCH	SMI	SNOMED	169061000006112
13746004	[X]Manic-depressive reaction	BPD	SMI	SNOMED	396071000006119
191627008	Bipolar affective disorder, currently depressed, unspecified	BPD	SMI	SNOMED	294861010
191559008	Unspecified latent schizophrenia	PSY	SMI	SNOMED	294765012
191574005	Schizoaffective schizophrenia, in remission	SCH	SMI	SNOMED	4763941000006113
85248005	[X]Bipolar affective disorder, currently in remission	BPD	SMI	SNOMED	296130018

Code	Term	Group	ParentGroup	CodingSystem	medcodeid
61831009	Folie Ã deux	PSY	SMI	SNOMED	3504731000006119
191554003	Subchronic paranoid schizophrenia with acute exacerbation	SCH	SMI	SNOMED	4763771000006119
61403008	[X]Bipol aff disord, curr epis sev depress, no psychot symp	BPD	SMI	SNOMED	367051000006112
191531007	Acute exacerbation of chronic schizophrenia	SCH	SMI	SNOMED	294731011
268619003	Manic disorder, single episode NOS	BPD	SMI	SNOMED	294809010
191667009	Paranoia	PSY	SMI	SNOMED	882331000006116
278853003	[X]Brief schizophrenifrm psych	PSY	SMI	SNOMED	368011000006110
191525009	Other nonorganic psychoses	PSY	SMI	SNOMED	25461000006115
191572009	Acute exacerbation of chronic schizoaffective schizophrenia	SCH	SMI	SNOMED	4763921000006118
13746004	Other and unspecified manic-depressive psychoses	BPD	SMI	SNOMED	294891017
231494001	Manic	BPD	SMI	SNOMED	5023961000006111
307504004	Oneirophrenia	PSY	SMI	SNOMED	450785011
231494001	[X]Mania with psychotic symptoms	BPD	SMI	SNOMED	401863011
765176007	Bipolar affect disord, now depressed, severe with psychosis	BPD	SMI	SNOMED	513721000006114
191683009	Psychogenic stupor	PSY	SMI	SNOMED	294927012
64905009	[X]Paraphrenic schizophrenia	SCH	SMI	SNOMED	418261000006112
26025008	[X]Restzustand schizophrenic	SCH	SMI	SNOMED	424931000006113
191597008	Recurrent manic episodes, in full remission	BPD	SMI	SNOMED	294817019
35252006	[X]Hebephrenic schizophrenia	SCH	SMI	SNOMED	388741000006110
191567000	Cyclic schizophrenia	PSY	SMI	SNOMED	294773015
441704009	Other affective psychosis NOS	BPD	SMI	SNOMED	294902017
61831009	Induced psychotic disorder	PSY	SMI	SNOMED	3504721000006117
191559008	[X]Latent schizophrenic reaction	PSY	SMI	SNOMED	395031000006114
83225003	[X]Bipolar affective disorder type II	BPD	SMI	SNOMED	1785861000006112

Code	Term	Group	ParentGroup	CodingSystem	medcodeid
280427006	[X]Symptomatic psychosis NOS	PSY	SMI	SNOMED	428451000006119
63249007	Bipolar affect disord,currently manic, part/unspec remission	BPD	SMI	SNOMED	513741000006119
191561004	Subchronic latent schizophrenia	PSY	SMI	SNOMED	294766013
14291003	Subchronic disorganized schizophrenia with acute exacerbations	SCH	SMI	SNOMED	2726271000006118
63181006	Paranoid schizophrenia, in remission	SCH	SMI	SNOMED	3527411000006119
274952002	[X]Borderline schizophrenia	SCH	SMI	SNOMED	367951000006113
31373002	Disorganised schizophrenia in remission	SCH	SMI	SNOMED	3005591000006114
191547009	Subchronic catatonic schizophrenia with acute exacerbation	SCH	SMI	SNOMED	4763731000006117
191565008	Latent schizophrenia, in remission	SCH	SMI	SNOMED	4763861000006116
58214004	[X]Schizophreniform disord NOS	SCH	SMI	SNOMED	425681000006117
231437006	Reactive psychoses	PSY	SMI	SNOMED	346896012
16506000	Bipolar I disorder, most recent episode mixed	BPD	SMI	SNOMED	2761681000006119
268622001	Chronic paranoid psychosis	PSY	SMI	SNOMED	401768012
231489001	[X]Acute and transient psychotic disorder, unspecified	PSY	SMI	SNOMED	401860014
231487004	[X]Delusional dysmorphophobia	PSY	SMI	SNOMED	376501000006110
231437006	Reactive psychosis	PSY	SMI	SNOMED	5023391000006116
1975081000006106	Undifferentiated schizophrenia, complete remission	SCH	SMI	SNOMED	1975081000006110
31446002	Bipolar I disorder, most recent episode hypomanic	BPD	SMI	SNOMED	3006671000006118
755311000000100	[X]Nonorganic psychosis in remission	PSY	SMI	SNOMED	1667591000000111
851691000006105	Hypomanic	BPD	SMI	SNOMED	851691000006114
191542003	[X]Schizophrenic flexibilatis cerea	SCH	SMI	SNOMED	294743012
191670008	Shared paranoid disorder	PSY	SMI	SNOMED	294909014
280949006	[X]Erotomania	PSY	SMI	SNOMED	2532965018
111484002	[X]Atypical schizophrenia	SCH	SMI	SNOMED	366571000006114

Code	Term	Group	ParentGroup	CodingSystem	medcodeid
480111000000107	[X]Chronic hallucinatory psychosis	PSY	SMI	SNOMED	370981000006112
64905009	Paranoid schizophrenia	SCH	SMI	SNOMED	107878010
191620005	Bipolar affective disorder, currently manic, mild	BPD	SMI	SNOMED	294849019
191554003	Acute exacerbation of subchronic paranoid schizophrenia	SCH	SMI	SNOMED	294757014
191595000	Recurrent manic episodes, severe, with psychosis	BPD	SMI	SNOMED	294815010
191555002	Chronic paranoid schizophrenia with acute exacerbation	SCH	SMI	SNOMED	4763791000006118
247804008	[X]Prodromal schizophrenia	PSY	SMI	SNOMED	423471000006117
231494001	[X]Mania with mood-congruent psychotic symptoms	BPD	SMI	SNOMED	396701000006111
191563001	Acute exacerbation of subchronic latent schizophrenia	PSY	SMI	SNOMED	294768014
417601000000102	[X]Schizophrenia, schizotypal and delusional disorders	PSY	SMI	SNOMED	296022017
191577003	Coenesthopathic schizophrenia	SCH	SMI	SNOMED	294787015
1975211000006106	Post-schizophrenic depression, episodic with progressive deficit	SCH	SMI	SNOMED	1975211000006110
231496004	Hypomanic psychoses	BPD	SMI	SNOMED	789221000006116
191667009	[X]Paranoid state	PSY	SMI	SNOMED	418231000006115
231485007	[X]Post-schizophrenic depression	SCH	SMI	SNOMED	423041000006119
191574005	Schizo-affective schizophrenia in remission	SCH	SMI	SNOMED	155151000006119
191627008	Bipolar affective disorder, currently depressed	BPD	SMI	SNOMED	513751000006117
63181006	Paranoid schizophrenia in remission	SCH	SMI	SNOMED	105029017
191592002	Recurrent manic episodes, mild	BPD	SMI	SNOMED	294812013
191593007	Recurrent manic episodes, moderate	BPD	SMI	SNOMED	294813015
231437006	Other reactive psychoses NOS	PSY	SMI	SNOMED	294929010
83746006	Chronic schizophrenia	SCH	SMI	SNOMED	3862661000006112
278853003	[X]Oneirophrenia	PSY	SMI	SNOMED	403611000006116

Code	Term	Group	ParentGroup	CodingSystem	medcodeid
4441000	Unspecified bipolar affective disorder,severe with psychosis	BPD	SMI	SNOMED	82171000006116
191526005	Schizophrenic psychoses NOS	SCH	SMI	SNOMED	882281000006113
278853003	[X]Brief schizophreniform disorder	PSY	SMI	SNOMED	368001000006112
161468000	H/O: schizophrenia	SCH	SMI	SNOMED	251628010
765176007	[X]Manic-depress psychosis,depressed type+psychotic symptoms	BPD	SMI	SNOMED	396781000006119
307417003	[X]Cycloid psychosis	PSY	SMI	SNOMED	376271000006119
35252006	Unspecified hebephrenic schizophrenia	SCH	SMI	SNOMED	294735019
191569002	Subchronic schizo-affective schizophrenia	SCH	SMI	SNOMED	123611000006110
48500005	[X]Delusional disorder	PSY	SMI	SNOMED	215841000000114
64905009	Paranoid schizophrenia NOS	SCH	SMI	SNOMED	294760019
111482003	Acute exacerbation of subchronic schizophrenia	SCH	SMI	SNOMED	294730012
191542003	Unspecified catatonic schizophrenia	SCH	SMI	SNOMED	294744018
764591000000108	Mixed bipolar affective disorder, severe, without psychosis	BPD	SMI	SNOMED	701071000006111
1975601000006106	Simple schizophrenia, complete remission	SCH	SMI	SNOMED	1975601000006110
13746004	Unspecified bipolar affective disorder, NOS	BPD	SMI	SNOMED	294888017
111484002	[X]Undifferentiated schizophrenia	SCH	SMI	SNOMED	296031017
27387000	Subchronic hebephrenic schizophrenia	SCH	SMI	SNOMED	294736018
231494001	Manic psychoses	PSY	SMI	SNOMED	223601000000119
268619003	[X]Bipolar disorder, single manic episode	BPD	SMI	SNOMED	367151000006111
13746004	Bipolar psychoses	BPD	SMI	SNOMED	513871000006118
764731000000103	Single manic episode in partial or unspecified remission	BPD	SMI	SNOMED	294807012
191630001	Bipolar affective disorder, currently depressed, moderate	BPD	SMI	SNOMED	294863013
41836007	Bipolar disorder, full remission	BPD	SMI	SNOMED	3174071000006116
417601000000102	[X]Schizophrenia, schizotypal and delusional disorders	SCH	SMI	SNOMED	12480451000006112

Code	Term	Group	ParentGroup	CodingSystem	medcodeid
58214004	[X]Other schizophrenia	SCH	SMI	SNOMED	401855010
53049002	Unspecified bipolar affective disorder, severe, no psychosis	BPD	SMI	SNOMED	82151000006114

Table 4: Ethnicity_SNOMED_Read_EMIS

Code	Term	Group	CodingSystem	medcodeid
286009	Czech	White	SNOMED	1160331000000119
18167009	Black African	Black	SNOMED	30683015
29343004	Bulgarian	White	SNOMED	1158301000000115
33897005	Black/Afri/Carib/Black Brit: Caribbean- NI eth cat 2011 cens	Black	SNOMED	550541000006110
33897005	Chinese	Asian	SNOMED	550541000006110
33897005	RACE: Chinese	Asian	SNOMED	550541000006110
36329002	Slovak	White	SNOMED	1551471000000116
38361009	RACE: Korean	Asian	SNOMED	196671000006119
40182006	Traveller - gypsy	White	SNOMED	3146431000006116
69865008	Asian/Asian British: Chinese - NI ethnic cat 2011 census	Asian	SNOMED	501416013
69865008	Fijian	Other	SNOMED	501416013
80208004	Portuguese	White	SNOMED	133078012
81560001	Asian or Asian British: Indian - NI ethnic cat 2011 census	Asian	SNOMED	504723011
81560001	Tongan	Other	SNOMED	504723011
86275006	Mixed: White and Asian - NI ethnic category 2011 census	Mixed	SNOMED	507015012
86275006	Samoan	Other	SNOMED	507015012
90027003	RACE: Arab	Other	SNOMED	196621000006115
103579009	Race: Other	Other	SNOMED	371005013
160513005	European origin	White	SNOMED	250222012

Code	Term	Group	CodingSystem	medcodeid
160514004	African origin	Black	SNOMED	250223019
160515003	Asian origin	Asian	SNOMED	250224013
160516002	North American origin	White	SNOMED	250225014
160517006	South American origin	White	SNOMED	250226010
160518001	Australian origin	Other	SNOMED	250227018
160519009	Indian origin	Asian	SNOMED	250228011
160520003	Middle Eastern origin	Other	SNOMED	250229015
160521004	Far Eastern origin	Asian	SNOMED	250230013
160522006	West Indian origin	Black	SNOMED	250231012
160531006	Race: West indian	Black	SNOMED	250243013
162730000	O/E - ethnic group NOS	Other	SNOMED	253627011
162731001	O/E - Europeanoid	White	SNOMED	253629014
162732008	O/E - Negroid	Black	SNOMED	253630016
162734009	O/E - Australoid	Other	SNOMED	253634013
185984009	Other white ethnic group	White	SNOMED	285925010
185984009	White	White	SNOMED	285925010
185988007	Black Caribbean	Black	SNOMED	285930014
185989004	Black Black - other	Black	SNOMED	285931013
185989004	Black, other, non-mixed origin	Black	SNOMED	285931013
185990008	Black British	Black	SNOMED	285932018
185993005	Black - other African country	Black	SNOMED	285943014
185995003	Black Indian sub-continent	Black	SNOMED	285948017
185996002	Black - other Asian	Mixed	SNOMED	285949013
185998001	Black - other, mixed	Black	SNOMED	285951012
185999009	Other Black - Black/White orig	Mixed	SNOMED	285952017
186000006	Other Black - Black/Asian orig	Mixed	SNOMED	285953010
186002003	Pakistani	Asian	SNOMED	285955015
186002003	RACE: Pakistani	Asian	SNOMED	285955015
186003008	Bangladeshi	Asian	SNOMED	285956019

Code	Term	Group	CodingSystem	medcodeid
186003008	RACE: Bangladeshi	Asian	SNOMED	285956019
186005001	Other ethnic NEC (NMO)	Other	SNOMED	285988010
186005001	Other ethnic non-mixed (NMO)	Other	SNOMED	285988010
186006000	Brit. ethnic minor. spec.(NMO)	Other	SNOMED	285959014
186007009	Brit. ethnic minor. unsp (NMO)	Other	SNOMED	4740261000006118
186010002	Other African countries (NMO)	Black	SNOMED	285971015
186012005	Indian sub-continent (NMO)	Asian	SNOMED	285976013
186013000	Other Asian (NMO)	Asian	SNOMED	285977016
186014006	Irish (NMO)	White	SNOMED	285978014
186017004	Other European (NMO)	White	SNOMED	285987017
186019001	Other ethnic, mixed origin	Mixed	SNOMED	285989019
186020007	Other ethnic, Black/White orig	Mixed	SNOMED	285990011
186021006	Other ethnic, Asian/White orig	Mixed	SNOMED	285991010
186022004	Other ethnic, mixed white orig	White	SNOMED	285992015
186023009	Other ethnic, other mixed orig	Mixed	SNOMED	285993013
186035008	New Zealand ethnic group NOS	Other	SNOMED	286006015
186035008	New Zealand ethnic groups	Other	SNOMED	286006015
186035008	Other New Zealand ethnic group	Other	SNOMED	286006015
186036009	Irish Traveller - Northern Ireland ethnic cat 2011 census	White	SNOMED	286008019
186036009	New Zealand European	White	SNOMED	286008019
186036009	Pakeha	White	SNOMED	286008019
186037000	Mixed: White and Black Caribbean - NI ethnic cat 2011 census	Mixed	SNOMED	286009010
186037000	Other European in New Zealand	White	SNOMED	286009010
186039002	Mixed: White and Black African - NI ethnic cat 2011 census	Mixed	SNOMED	286012013
186039002	New Zealand Maori	Other	SNOMED	286012013
186040000	Cook Island Maori	Other	SNOMED	286013015

Code	Term	Group	CodingSystem	medcodeid
186040000	Mixed: other Mixed/multiple ethnic backgrd - NI 2011 census	Mixed	SNOMED	286013015
186041001	Asian/Asian British: Pakistani - NI ethnic cat 2011 census	Asian	SNOMED	286014014
186041001	Niuean	Other	SNOMED	286014014
186042008	Asian/Asian British: Bangladeshi - NI ethnic cat 2011 census	Asian	SNOMED	4740541000006115
186042008	Tokelauan	Other	SNOMED	4740541000006115
186044009	Black/Afri/Carib/Black Brit: African- NI eth cat 2011 census	Black	SNOMED	286018012
186044009	South East Asian	Asian	SNOMED	286018012
268914005	O/E - Mongoloid origin	Asian	SNOMED	402434017
270460000	Black Caribbean/W.I./Guyana	Black	SNOMED	5516681000006114
270461001	Black N African/Arab/Iranian	Black	SNOMED	405065012
270462008	Black E Afric Asia/Indo-Caribb	Mixed	SNOMED	514651000006112
270463003	Caribbean I./W.I./Guyana (NMO)	Black	SNOMED	405067016
270464009	N African Arab/Iranian (NMO)	Other	SNOMED	405068014
270465005	E Afric Asian/Indo-Carib (NMO)	Asian	SNOMED	405069018
270466006	Greek/Greek Cypriot (NMO)	White	SNOMED	405070017
270467002	Turkish/Turkish Cypriot (NMO)	White	SNOMED	405071018
275586009	Black North African	Black	SNOMED	411573013
275587000	Black Arab	Black	SNOMED	411574019
275588005	Black Iranian	Black	SNOMED	411575018
275589002	Black East African Asian	Mixed	SNOMED	411576017
275590006	Black Indo-Caribbean	Mixed	SNOMED	411577014
275591005	Caribbean Island (NMO)	Black	SNOMED	411578016
275592003	West Indian (NMO)	Black	SNOMED	411579012
275593008	Guyana (NMO)	Black	SNOMED	411580010
275594002	North African Arab (NMO)	Other	SNOMED	411581014

Code	Term	Group	CodingSystem	medcodeid
275595001	Iranian (NMO)	Other	SNOMED	411582019
275596000	East African Asian (NMO)	Asian	SNOMED	411583012
275597009	Indo-Caribbean (NMO)	Asian	SNOMED	411584018
275599007	Greek (NMO)	White	SNOMED	411594011
275600005	Greek Cypriot (NMO)	White	SNOMED	411595012
275601009	Turkish (NMO)	White	SNOMED	411596013
275602002	Turkish Cypriot (NMO)	White	SNOMED	411597016
276029002	O/E - Asian origin	Asian	SNOMED	412016016
309643000	Black West Indian	Black	SNOMED	453109012
309644006	Black Guyana	Black	SNOMED	453110019
312859007	Vietnamese	Asian	SNOMED	456650013
312861003	Ethnic group not recorded	Not recorded	SNOMED	456652017
315236000	White British	White	SNOMED	459726019
315237009	White Irish	White	SNOMED	459727011
315279003	Other black ethnic group	Black	SNOMED	459782019
315280000	Other Asian	Asian	SNOMED	286020010
315280000	Other ethnic group: Arab - NI ethnic category 2011 census	Other	SNOMED	286020010
315281001	Other Asian ethnic group	Asian	SNOMED	459784018
315283003	Irish traveller	White	SNOMED	459786016
315634007	Black Caribbean and White	Mixed	SNOMED	460153018
315635008	Black African and White	Mixed	SNOMED	460154012
372148003	Asian/Asian British: other Asian - NI ethnic cat 2011 census	Asian	SNOMED	459785017
372148003	Other ethnic group	Other	SNOMED	459785017
372148003	Other Pacific ethnic group	Other	SNOMED	459785017
397731000	Ethnic groups (census) NOS	Other	SNOMED	6591901000006115
401213008	White Scottish	White	SNOMED	1780407014

Code	Term	Group	CodingSystem	medcodeid
401214002	Other white British ethnic group	White	SNOMED	1780408016
413465009	RACE: Afro-caribbean	Black	SNOMED	196601000006113
413466005	RACE: Afro-caucasian	Mixed	SNOMED	196611000006111
413773004	RACE: Caucasian	White	SNOMED	196641000006110
413773004	Race: White	White	SNOMED	196641000006110
414481008	Black/Afri/Carib/Black Brit: other - NI eth cat 2011 census	Black	SNOMED	285954016
414481008	Indian	Asian	SNOMED	285954016
414551003	RACE: Japanese	Asian	SNOMED	196661000006114
414752008	RACE: Mixed	Mixed	SNOMED	196681000006116
414978006	RACE: Oriental	Asian	SNOMED	196701000006118
415226007	RACE: Not stated	Not recorded	SNOMED	196691000006118
415794004	RACE: Unknown	Other	SNOMED	196731000006114
445343003	Romanian	White	SNOMED	1158211000000111
763726001	Ethnic group not given - patient refused	Not recorded	SNOMED	12009371000006110
86461000000107	Sri Lankan - ethnic category 2001 census	Asian	SNOMED	136081000000111
88911000000101	Irish Traveller - ethnic category 2001 census	White	SNOMED	138171000000114
88921000000107	Traveller - ethnic category 2001 census	White	SNOMED	138181000000111
88931000000109	Gypsy/Romany - ethnic category 2001 census	White	SNOMED	138191000000113
88941000000100	Polish - ethnic category 2001 census	White	SNOMED	138201000000110
88951000000102	Baltic Estonian/Latvian/Lithuanian - ethn categ 2001 census	White	SNOMED	937301000006110
88961000000104	Commonwealth (Russian) Indep States - ethn categ 2001 census	White	SNOMED	937311000006113
88971000000106	Albanian - ethnic category 2001 census	White	SNOMED	138231000000116
88981000000108	Serbian - ethnic category 2001 census	White	SNOMED	157991000000110
88991000000105	Jewish - ethnic category 2001 census	White	SNOMED	138241000000113

Code	Term	Group	CodingSystem	medcodeid
89001000000105	Arab - ethnic category 2001 census	Other	SNOMED	138251000000111
89011000000107	Iranian - ethnic category 2001 census	Other	SNOMED	138261000000114
89021000000101	South and Central American - ethnic category 2001 census	White	SNOMED	138271000000119
89031000000104	Muslim - ethnic category 2001 census	Other	SNOMED	138281000000117
92391000000108	British or mixed British - ethnic category 2001 census	White	SNOMED	158341000000117
92401000000106	Irish - ethnic category 2001 census	White	SNOMED	141301000000110
92411000000108	Other White background - ethnic category 2001 census	White	SNOMED	141311000000112
92421000000102	White and Black Caribbean - ethnic category 2001 census	Mixed	SNOMED	141321000000118
92431000000100	White and Black African - ethnic category 2001 census	Mixed	SNOMED	141331000000116
92441000000109	White and Asian - ethnic category 2001 census	Mixed	SNOMED	141341000000113
92451000000107	Other Mixed background - ethnic category 2001 census	Mixed	SNOMED	141351000000111
92461000000105	Pakistani or British Pakistani - ethnic category 2001 census	Asian	SNOMED	141361000000114
92471000000103	Bangladeshi or British Bangladeshi - ethn categ 2001 census	Asian	SNOMED	937541000006115
92481000000101	Other Asian background - ethnic category 2001 census	Asian	SNOMED	141381000000117
92491000000104	African - ethnic category 2001 census	Black	SNOMED	141391000000115
92501000000105	Other Black background - ethnic category 2001 census	Black	SNOMED	158351000000119
92511000000107	Chinese - ethnic category 2001 census	Asian	SNOMED	141401000000117
92521000000101	Other - ethnic category 2001 census	Other	SNOMED	141411000000115
92531000000104	Ethnic category not stated - 2001 census	Not recorded	SNOMED	141421000000114

Code	Term	Group	CodingSystem	medcodeid
92541000000108	Scottish - ethnic category 2001 census	White	SNOMED	141431000000111
92551000000106	Welsh - ethnic category 2001 census	White	SNOMED	141441000000119
92561000000109	Northern Irish - ethnic category 2001 census	White	SNOMED	141451000000116
92571000000102	Cornish - ethnic category 2001 census	White	SNOMED	141461000000118
92581000000100	Black and Asian - ethnic category 2001 census	Mixed	SNOMED	141471000000113
92591000000103	Black and Chinese - ethnic category 2001 census	Mixed	SNOMED	141481000000110
92601000000109	Chinese and White - ethnic category 2001 census	Mixed	SNOMED	141491000000112
92611000000106	Asian and Chinese - ethnic category 2001 census	Asian	SNOMED	158361000000116
92621000000100	Other Mixed or Mixed unspecified ethnic category 2001 census	Mixed	SNOMED	937511000006119
92631000000103	Mixed Asian - ethnic category 2001 census	Asian	SNOMED	141511000000116
92641000000107	Punjabi - ethnic category 2001 census	Asian	SNOMED	141521000000110
92651000000105	Kashmiri - ethnic category 2001 census	Asian	SNOMED	141531000000112
92661000000108	East African Asian - ethnic category 2001 census	Asian	SNOMED	141541000000115
92671000000101	Tamil - ethnic category 2001 census	Asian	SNOMED	141551000000117
92681000000104	British Asian - ethnic category 2001 census	Asian	SNOMED	141561000000119
92691000000102	Caribbean Asian - ethnic category 2001 census	Asian	SNOMED	141571000000114
92701000000102	Other Asian or Asian unspecified ethnic category 2001 census	Asian	SNOMED	937651000006117
92711000000100	Somali - ethnic category 2001 census	Black	SNOMED	141591000000113
92721000000106	Mixed Black - ethnic category 2001 census	Black	SNOMED	158371000000111
92731000000108	Nigerian - ethnic category 2001 census	Black	SNOMED	141601000000119
92741000000104	Other Black or Black unspecified ethnic category 2001 census	Black	SNOMED	937731000006115
92751000000101	Vietnamese - ethnic category 2001 census	Asian	SNOMED	141621000000111
92761000000103	Japanese - ethnic category 2001 census	Asian	SNOMED	141631000000113
92771000000105	Filipino - ethnic category 2001 census	Asian	SNOMED	141641000000116
92781000000107	Malaysian - ethnic category 2001 census	Asian	SNOMED	141651000000118

Code	Term	Group	CodingSystem	medcodeid
92791000000109	Cypriot (part not stated) - ethnic category 2001 census	White	SNOMED	141661000000115
93921000000101	Ulster Scots - ethnic category 2001 census	White	SNOMED	142691000000116
93931000000104	Greek - ethnic category 2001 census	White	SNOMED	142701000000116
93941000000108	Greek Cypriot - ethnic category 2001 census	White	SNOMED	142711000000119
93951000000106	Turkish Cypriot - ethnic category 2001 census	White	SNOMED	142721000000113
93961000000109	Italian - ethnic category 2001 census	White	SNOMED	158481000000115
93981000000100	Kosovan - ethnic category 2001 census	White	SNOMED	142741000000118
93991000000103	Bosnian - ethnic category 2001 census	White	SNOMED	142751000000115
94001000000108	Croatian - ethnic category 2001 census	White	SNOMED	142761000000117
94011000000105	Other republics former Yugoslavia - ethnic categ 2001 census	White	SNOMED	937371000006116
94021000000104	Mixed Irish and other White - ethnic category 2001 census	White	SNOMED	142781000000114
94031000000102	Other mixed White - ethnic category 2001 census	White	SNOMED	142791000000111
94041000000106	Oth White European/European unsp/Mixed European 2001 census	White	SNOMED	937391000006115
94051000000109	Other White or White unspecified ethnic category 2001 census	White	SNOMED	937411000006115
94061000000107	North African - ethnic category 2001 census	Other	SNOMED	142811000000112
94071000000100	Mid East (excl Israeli, Iranian & Arab) - eth cat 2001 cens	Other	SNOMED	937871000006114
94081000000103	Israeli - ethnic category 2001 census	White	SNOMED	142831000000116
94091000000101	Kurdish - ethnic category 2001 census	Other	SNOMED	142841000000113
94101000000109	Moroccan - ethnic category 2001 census	Other	SNOMED	142851000000111
94111000000106	Latin American - ethnic category 2001 census	White	SNOMED	142861000000114
94121000000100	Mauritian/Seychellois/Maldivian/St Helena eth cat 2001census	Asian	SNOMED	937941000006111
94131000000103	Buddhist - ethnic category 2001 census	Other	SNOMED	142881000000117

Code	Term	Group	CodingSystem	medcodeid
94141000000107	Sikh - ethnic category 2001 census	Other	SNOMED	142891000000115
94151000000105	Any other group - ethnic category 2001 census	Other	SNOMED	142901000000119
107691000000105	Caribbean - ethnic category 2001 census	Black	SNOMED	154401000000118
110401000000103	Turkish - ethnic category 2001 census	White	SNOMED	156921000000110
110751000000108	Indian or British Indian - ethnic category 2001 census	Asian	SNOMED	157271000000119
110761000000106	English - ethnic category 2001 census	White	SNOMED	157281000000117
110771000000104	Black and White - ethnic category 2001 census	Mixed	SNOMED	157291000000115
110781000000102	Sinhalese - ethnic category 2001 census	Asian	SNOMED	157301000000116
110791000000100	Black British - ethnic category 2001 census	Black	SNOMED	157311000000119
110831000000107	Hindu - ethnic category 2001 census	Other	SNOMED	157351000000115
296841000000102	Yemeni	Other	SNOMED	523591000000116
494131000000105	White British - ethnic category 2001 census	White	SNOMED	1063981000000117
494161000000100	White Irish - ethnic category 2001 census	White	SNOMED	1064041000000111
718131000000106	Nepali	Asian	SNOMED	1572831000000110
907641000000103	British or mixed British - ethnic category 2001 census	White	SNOMED	2333461000000112
976591000000101	New Zealand ethnic groups	Other	SNOMED	2484551000000111
976611000000109	Traveller - gypsy	White	SNOMED	2484591000000115
976631000000101	White:Eng/Welsh/Scot/NI/Brit - England and Wales 2011 census	White	SNOMED	1968051000006116
976651000000108	White: Irish - England and Wales ethnic category 2011 census	White	SNOMED	2484671000000118
976671000000104	White: Gypsy/Irish Traveller - Eng+Wales eth cat 2011 census	White	SNOMED	1968071000006114
976691000000100	White: other White backgrd- Eng+Wales ethnic cat 2011 census	White	SNOMED	1968081000006112
976711000000103	Mixed: White+Black Caribbean - Eng+Wales eth cat 2011 census	Mixed	SNOMED	1968091000006110

Code	Term	Group	CodingSystem	medcodeid
976731000000106	Mixed: White+Black African - Eng+Wales eth cat 2011 census	Mixed	SNOMED	1968101000006116
976751000000104	Mixed: White+Asian - Eng+Wales ethnic category 2011 census	Mixed	SNOMED	1968111000006118
976771000000108	Mixed: other Mixed/multiple backgrd - Eng+Wales 2011 census	Mixed	SNOMED	1968121000006114
976791000000107	Asian/Asian Brit: Indian - Eng+Wales ethnic cat 2011 census	Asian	SNOMED	1968131000006112
976811000000108	Asian/Asian British:Pakistani- Eng+Wales eth cat 2011 census	Asian	SNOMED	1968141000006119
976831000000100	Asian/Asian Brit: Bangladeshi- Eng+Wales eth cat 2011 census	Asian	SNOMED	1968151000006117
976851000000107	Asian/Asian Brit: Chinese - Eng+Wales ethnic cat 2011 census	Asian	SNOMED	1968161000006115
976871000000103	Asian/Asian Brit: other Asian- Eng+Wales eth cat 2011 census	Asian	SNOMED	1968171000006110
976891000000104	Black/African/Carib/Black Brit: African- Eng+Wales 2011 cens	Black	SNOMED	1968181000006113
976911000000101	Black/African/Caribbn/Black Brit: Caribbean - Eng+Wales 2011	Black	SNOMED	1968191000006111
976931000000109	Black/Afr/Carib/Black Brit: other Black- Eng+Wales 2011 cens	Black	SNOMED	1968201000006114
976951000000102	Other ethnic group: Arab - Eng+Wales ethnic cat 2011 census	Other	SNOMED	1968211000006112
976971000000106	Other ethnic: any other grp - Eng+Wales eth cat 2011 census	Other	SNOMED	1968221000006116
977351000000100	White - Northern Ireland ethnic category 2011 census	White	SNOMED	2486161000000112
977371000000109	Irish Traveller - Northern Ireland ethnic cat 2011 census	White	SNOMED	1968251000006113
977371000000109	New Zealand European	White	SNOMED	1968251000006113

Code	Term	Group	CodingSystem	medcodeid
977391000000108	Mixed: White and Black Caribbean - NI ethnic cat 2011 census	Mixed	SNOMED	1968261000006110
977391000000108	Other European in New Zealand	White	SNOMED	1968261000006110
977411000000108	Mixed: White and Black African - NI ethnic cat 2011 census	Mixed	SNOMED	1968271000006115
977411000000108	New Zealand Maori	Other	SNOMED	1968271000006115
977431000000100	Mixed: White and Asian - NI ethnic category 2011 census	Mixed	SNOMED	1968281000006117
977431000000100	Samoan	Other	SNOMED	1968281000006117
977551000000106	Cook Island Maori	Other	SNOMED	1968291000006119
977551000000106	Mixed: other Mixed/multiple ethnic backgrd - NI 2011 census	Mixed	SNOMED	1968291000006119
977591000000103	Asian or Asian British: Indian - NI ethnic cat 2011 census	Asian	SNOMED	1968301000006118
977591000000103	Tongan	Other	SNOMED	1968301000006118
977711000000100	Asian/Asian British: Pakistani - NI ethnic cat 2011 census	Asian	SNOMED	1968311000006115
977711000000100	Niuean	Other	SNOMED	1968311000006115
977731000000108	Asian/Asian British: Bangladeshi - NI ethnic cat 2011 census	Asian	SNOMED	1968321000006111
977731000000108	Tokelauan	Other	SNOMED	1968321000006111
977751000000101	Asian/Asian British: Chinese - NI ethnic cat 2011 census	Asian	SNOMED	1968331000006114
977751000000101	Fijian	Other	SNOMED	1968331000006114
977771000000105	Asian/Asian British: other Asian - NI ethnic cat 2011 census	Asian	SNOMED	1968341000006116
977771000000105	Other Pacific ethnic group	Other	SNOMED	1968341000006116
977791000000109	Black/Afri/Carib/Black Brit: African- NI eth cat 2011 census	Black	SNOMED	1968351000006119
977791000000109	South East Asian	Asian	SNOMED	1968351000006119

Code	Term	Group	CodingSystem	medcodeid
977811000000105	Black/Afri/Carib/Black Brit: Caribbean- NI eth cat 2011 cens	Black	SNOMED	1968361000006117
977811000000105	Chinese	Asian	SNOMED	1968361000006117
977831000000102	Black/Afri/Carib/Black Brit: other - NI eth cat 2011 census	Black	SNOMED	1968371000006112
977831000000102	Indian	Asian	SNOMED	1968371000006112
977851000000109	Other Asian	Asian	SNOMED	1968381000006110
977851000000109	Other ethnic group: Arab - NI ethnic category 2011 census	Other	SNOMED	1968381000006110
977871000000100	Other ethnic group: any other grp- NI ethnic cat 2011 census	Other	SNOMED	1968391000006113
977911000000103	White: Scottish - Scotland ethnic category 2011 census	White	SNOMED	2487281000000112
977931000000106	White: other British - Scotland ethnic category 2011 census	White	SNOMED	2487321000000116
977951000000104	White: Irish - Scotland ethnic category 2011 census	White	SNOMED	2487361000000112
977971000000108	White: Gypsy/Irish Traveller - Scotland ethnic cat 2011 cens	White	SNOMED	1968441000006112
978011000000101	White: Polish - Scotland ethnic category 2011 census	White	SNOMED	2487481000000113
978031000000109	White: other White ethnic grp- Scotland ethnic cat 2011 cens	White	SNOMED	1968461000006111
978051000000102	Mixed/multiple ethnic grps: any- Scot ethnic cat 2011 census	Mixed	SNOMED	1968471000006116
978071000000106	Asian: Pakistani/Pakistani Scot/Pakistani Brit- Scot 2011	Asian	SNOMED	1968481000006118
978111000000100	Asian: Indian, Indian Scot/Indian Brit- Scotland 2011 census	Asian	SNOMED	1968491000006115
978171000000105	Bangladeshi, Bangladeshi Scot or Bangladeshi Brit- Scot 2011	Asian	SNOMED	1968501000006111

Code	Term	Group	CodingSystem	medcodeid
978191000000109	Asian: Chinese - Scotland ethnic category 2011 census	Asian	SNOMED	1968511000006114
978211000000108	Asian: other Asian group - Scotland ethnic cat 2011 census	Asian	SNOMED	1968521000006118
978231000000100	African: African/African Scot/African Brit - Scotland 2011	Black	SNOMED	1968531000006115
978251000000107	African: any other African - Scotland ethnic cat 2011 census	Black	SNOMED	1968541000006113
978271000000103	Carib/Black: Caribbean/Carib Scot/Carib Brit- Scotland 2011	Black	SNOMED	1968551000006110
978341000000102	Carib/Black: Black/Black Scot/Black Brit- Scotland 2011 cens	Black	SNOMED	1968561000006112
978361000000101	Carib/Black: any other Black/Caribbean grp - Scotland 2011	Black	SNOMED	1968571000006117
978381000000105	Other ethnic grp: Arab/Arab Scot/Arab British- Scotland 2011	Other	SNOMED	1968581000006119
978401000000105	Other ethnic grp: any other ethnic grp- Scotland 2011 census	Other	SNOMED	1968591000006116
9SA4-2	Iranian (NMO)	Other	Read	411582019
9SA5	Other African countries (NMO)	Black	Read	285971015
9SA6	E Afric Asian/Indo-Carib (NMO)	Asian	Read	405069018
9SA6-1	East African Asian (NMO)	Asian	Read	411583012
9SA6-2	Indo-Caribbean (NMO)	Asian	Read	411584018
9SA7	Indian sub-continent (NMO)	Asian	Read	285976013
9SA8	Other Asian (NMO)	Asian	Read	285977016
9SA9	Irish (NMO)	White	Read	285978014
9SAA	Greek/Greek Cypriot (NMO)	White	Read	405070017
9SAA-1	Greek (NMO)	White	Read	411594011
9SAA-2	Greek Cypriot (NMO)	White	Read	411595012
9SAB	Turkish/Turkish Cypriot (NMO)	White	Read	405071018

Code	Term	Group	CodingSystem	medcodeid
9SAB-1	Turkish (NMO)	White	Read	411596013
9SAB-2	Turkish Cypriot (NMO)	White	Read	411597016
9SAC	Other European (NMO)	White	Read	285987017
9SAD	Other ethnic NEC (NMO)	Other	Read	285988010
9SB	Other ethnic, mixed origin	Mixed	Read	285989019
9SB1	Other ethnic, Black/White orig	Mixed	Read	285990011
9SB2	Other ethnic, Asian/White orig	Mixed	Read	285991010
9SB3	Other ethnic, mixed white orig	White	Read	285992015
9SB4	Other ethnic, other mixed orig	Mixed	Read	285993013
9SB5	Black Caribbean and White	Mixed	Read	460153018
9SB6	Black African and White	Mixed	Read	460154012
9SC	Vietnamese	Asian	Read	456650013
9SD	Ethnic group not given - patient refused	Not recorded	Read	456651012
9SE	Ethnic group not recorded	Not recorded	Read	456652017
9SG	Other black ethnic group	Black	Read	459782019
9SH	Other Asian ethnic group	Asian	Read	459784018
9SI	Irish traveller	White	Read	459786016
9SJ	Other ethnic group	Other	Read	459785017
9SZ	Ethnic groups (census) NOS	Other	Read	286003011
9t00	White:Eng/Welsh/Scot/NI/Brit - England and Wales 2011 census	White	Read	1968051000006116
9t01	White: Irish - England and Wales ethnic category 2011 census	White	Read	2484671000000118
9t02	White: Gypsy/Irish Traveller - Eng+Wales eth cat 2011 census	White	Read	1968071000006114
9t03	White: other White backgrd- Eng+Wales ethnic cat 2011 census	White	Read	1968081000006112

Code	Term	Group	CodingSystem	medcodeid
9t04	Mixed: White+Black Caribbean - Eng+Wales eth cat 2011 census	Mixed	Read	1968091000006110
9t05	Mixed: White+Black African - Eng+Wales eth cat 2011 census	Mixed	Read	1968101000006116
9t06	Mixed: White+Asian - Eng+Wales ethnic category 2011 census	Mixed	Read	1968111000006118
9t07	Mixed: other Mixed/multiple backgrd - Eng+Wales 2011 census	Mixed	Read	1968121000006114
9t08	Asian/Asian Brit: Indian - Eng+Wales ethnic cat 2011 census	Asian	Read	1968131000006112
9t09	Asian/Asian British:Pakistani- Eng+Wales eth cat 2011 census	Asian	Read	1968141000006119
9t0A	Asian/Asian Brit: Bangladeshi- Eng+Wales eth cat 2011 census	Asian	Read	1968151000006117
9t0B	Asian/Asian Brit: Chinese - Eng+Wales ethnic cat 2011 census	Asian	Read	1968161000006115
9t0C	Asian/Asian Brit: other Asian- Eng+Wales eth cat 2011 census	Asian	Read	1968171000006110
9t0D	Black/African/Carib/Black Brit: African- Eng+Wales 2011 cens	Black	Read	1968181000006113
9t0E	Black/African/Caribbn/Black Brit: Caribbean - Eng+Wales 2011	Black	Read	1968191000006111
9t0F	Black/Afr/Carib/Black Brit: other Black- Eng+Wales 2011 cens	Black	Read	1968201000006114
9t0G	Other ethnic group: Arab - Eng+Wales ethnic cat 2011 census	Other	Read	1968211000006112
9t0H	Other ethnic: any other grp - Eng+Wales eth cat 2011 census	Other	Read	1968221000006116
9T1	New Zealand ethnic groups	Other	Read	286006015
9t10	White - Northern Ireland ethnic category 2011 census	White	Read	2486161000000112

Code	Term	Group	CodingSystem	medcodeid
9t11	Irish Traveller - Northern Ireland ethnic cat 2011 census	White	Read	286007012
9T11	New Zealand European	White	Read	286007012
9T11-1	Pakeha	White	Read	286008019
9t12	Mixed: White and Black Caribbean - NI ethnic cat 2011 census	Mixed	Read	1968261000006110
9T12	Other European in New Zealand	White	Read	1968261000006110
9t13	Mixed: White and Black African - NI ethnic cat 2011 census	Mixed	Read	1968271000006115
9T13	New Zealand Maori	Other	Read	1968271000006115
9t14	Mixed: White and Asian - NI ethnic category 2011 census	Mixed	Read	1968281000006117
9T14	Samoan	Other	Read	1968281000006117
9t15	Mixed: other Mixed/multiple ethnic backgrd - NI 2011 census	Mixed	Read	1968291000006119
9T15	Cook Island Maori	Other	Read	1968291000006119
9t16	Asian or Asian British: Indian - NI ethnic cat 2011 census	Asian	Read	504723011
9T16	Tongan	Other	Read	504723011
9t1C	Black/Afri/Carib/Black Brit: Caribbean- NI eth cat 2011 cens	Black	Read	550541000006110
9T1C	Chinese	Asian	Read	550541000006110
9t1D	Black/Afri/Carib/Black Brit: other - NI eth cat 2011 census	Black	Read	1968371000006112
9T1D	Indian	Asian	Read	1968371000006112
9t1E	Other ethnic group: Arab - NI ethnic category 2011 census	Other	Read	286020010
9T1E	Other Asian	Asian	Read	286020010
9t1F	Other ethnic group: any other grp- NI ethnic cat 2011 census	Other	Read	1968391000006113

Code	Term	Group	CodingSystem	medcodeid
9T1Y	Other New Zealand ethnic group	Other	Read	286021014
9T1Z	New Zealand ethnic group NOS	Other	Read	286022019
9T2	Traveller - gypsy	White	Read	2484591000000115
9t20	White: Scottish - Scotland ethnic category 2011 census	White	Read	2487281000000112
9t21	White: other British - Scotland ethnic category 2011 census	White	Read	2487321000000116
9t22	White: Irish - Scotland ethnic category 2011 census	White	Read	2487361000000112
9t23	White: Gypsy/Irish Traveller - Scotland ethnic cat 2011 cens	White	Read	1968441000006112
9t24	White: Polish - Scotland ethnic category 2011 census	White	Read	2487481000000113
9t25	White: other White ethnic grp- Scotland ethnic cat 2011 cens	White	Read	1968461000006111
9t26	Mixed/multiple ethnic grps: any- Scot ethnic cat 2011 census	Mixed	Read	1968471000006116
9t27	Asian: Pakistani/Pakistani Scot/Pakistani Brit-Scot 2011	Asian	Read	1968481000006118
9t28	Asian: Indian, Indian Scot/Indian Brit- Scotland 2011 census	Asian	Read	1968491000006115
9t29	Bangladeshi, Bangladeshi Scot or Bangladeshi Brit- Scot 2011	Asian	Read	1968501000006111
9t2A	Asian: Chinese - Scotland ethnic category 2011 census	Asian	Read	1968511000006114
9t2B	Asian: other Asian group - Scotland ethnic cat 2011 census	Asian	Read	1968521000006118
9t2C	African: African/African Scot/African Brit - Scotland 2011	Black	Read	1968531000006115
9t2D	African: any other African - Scotland ethnic cat 2011 census	Black	Read	1968541000006113

Code	Term	Group	CodingSystem	medcodeid
9t2E	Carib/Black: Caribbean/Carib Scot/Carib Brit-Scotland 2011	Black	Read	1968551000006110
9t2F	Carib/Black: Black/Black Scot/Black Brit-Scotland 2011 cens	Black	Read	1968561000006112
9t2G	Carib/Black: any other Black/Caribbean grp - Scotland 2011	Black	Read	1968571000006117
9t2H	Other ethnic grp: Arab/Arab Scot/Arab British-Scotland 2011	Other	Read	1968581000006119
9t2J	Other ethnic grp: any other ethnic grp- Scotland 2011 census	Other	Read	1968591000006116
9T3	Yemeni	Other	Read	523591000000116
9T4	Romanian	White	Read	1158211000000111
9T5	Bulgarian	White	Read	1158301000000115
9T6	Czech	White	Read	1160331000000119
9T7	Slovak	White	Read	1551471000000116
9T8	Portuguese	White	Read	133078012
9T9	Nepali	Asian	Read	1572831000000110
1341	European origin	White	Read	250222012
1342	African origin	Black	Read	250223019
1343	Asian origin	Asian	Read	250224013
1344	North American origin	White	Read	250225014
1345	South American origin	White	Read	250226010
1346	Australian origin	Other	Read	250227018
1347	Indian origin	Asian	Read	250228011
9iA	Other Asian background - ethnic category 2001 census	Asian	Read	141381000000117
9iA1	Punjabi - ethnic category 2001 census	Asian	Read	141521000000110
9iA2	Kashmiri - ethnic category 2001 census	Asian	Read	141531000000112
9iA3	East African Asian - ethnic category 2001 census	Asian	Read	141541000000115
9iA4	Sri Lankan - ethnic category 2001 census	Asian	Read	136081000000111

Code	Term	Group	CodingSystem	medcodeid
9iA5	Tamil - ethnic category 2001 census	Asian	Read	141551000000117
9iA6	Sinhalese - ethnic category 2001 census	Asian	Read	157301000000116
9iA7	Caribbean Asian - ethnic category 2001 census	Asian	Read	141571000000114
9iA8	British Asian - ethnic category 2001 census	Asian	Read	141561000000119
9iA9	Mixed Asian - ethnic category 2001 census	Asian	Read	141511000000116
9iAA	Other Asian or Asian unspecified ethnic category 2001 census	Asian	Read	937651000006117
9iB	Caribbean - ethnic category 2001 census	Black	Read	154401000000118
9iC	African - ethnic category 2001 census	Black	Read	141391000000115
9iD	Other Black background - ethnic category 2001 census	Black	Read	158351000000119
9iD0	Somali - ethnic category 2001 census	Black	Read	141591000000113
9iD1	Nigerian - ethnic category 2001 census	Black	Read	141601000000119
9iD2	Black British - ethnic category 2001 census	Black	Read	157311000000119
9iD3	Mixed Black - ethnic category 2001 census	Black	Read	158371000000111
9iD4	Other Black or Black unspecified ethnic category 2001 census	Black	Read	937731000006115
9iE	Chinese - ethnic category 2001 census	Asian	Read	141401000000117
9iF	Other - ethnic category 2001 census	Other	Read	141411000000115
9iF0	Vietnamese - ethnic category 2001 census	Asian	Read	141621000000111
9iF1	Japanese - ethnic category 2001 census	Asian	Read	141631000000113
9iF2	Filipino - ethnic category 2001 census	Asian	Read	141641000000116
9iF3	Malaysian - ethnic category 2001 census	Asian	Read	141651000000118
9iF4	Buddhist - ethnic category 2001 census	Other	Read	142881000000117
9iF5	Hindu - ethnic category 2001 census	Other	Read	157351000000115
9iF6	Jewish - ethnic category 2001 census	White	Read	138241000000113
9iF7	Muslim - ethnic category 2001 census	Other	Read	138281000000117
9iF8	Sikh - ethnic category 2001 census	Other	Read	142891000000115
9iF9	Arab - ethnic category 2001 census	Other	Read	138251000000111

Code	Term	Group	CodingSystem	medcodeid
9iFA	North African - ethnic category 2001 census	Other	Read	142811000000112
9iFB	Mid East (excl Israeli, Iranian & Arab) - eth cat 2001 cens	Other	Read	937871000006114
9iFC	Israeli - ethnic category 2001 census	White	Read	142831000000116
9iFD	Iranian - ethnic category 2001 census	Other	Read	138261000000114
9iFE	Kurdish - ethnic category 2001 census	Other	Read	142841000000113
9iFF	Moroccan - ethnic category 2001 census	Other	Read	142851000000111
9iFG	Latin American - ethnic category 2001 census	White	Read	142861000000114
9iFH	South and Central American - ethnic category 2001 census	White	Read	138271000000119
9iFJ	Mauritian/Seychellois/Maldivian/St Helena eth cat 2001census	Asian	Read	937941000006111
9iFK	Any other group - ethnic category 2001 census	Other	Read	142901000000119
9iG	Ethnic category not stated - 2001 census	Not recorded	Read	141421000000114
9S1	White	White	Read	285925010
9S10	White British	White	Read	459726019
9S11	White Irish	White	Read	459727011
9S12	Other white ethnic group	White	Read	459728018
9S13	White Scottish	White	Read	1780407014
9S14	Other white British ethnic group	White	Read	1780408016
9S2	Black Caribbean	Black	Read	514611000006111
9S3	Black African	Black	Read	30683015
9S4	Black, other, non-mixed origin	Black	Read	285931013
9S41	Black British	Black	Read	285932018
9S42	Black Caribbean/W.I./Guyana	Black	Read	405064011
9S42-1	Black Caribbean	Black	Read	285930014
9S42-2	Black West Indian	Black	Read	453109012
9S42-3	Black Guyana	Black	Read	453110019

Code	Term	Group	CodingSystem	medcodeid
9S43	Black N African/Arab/Iranian	Black	Read	405065012
9S43-1	Black North African	Black	Read	411573013
9S43-2	Black Arab	Black	Read	411574019
9S43-3	Black Iranian	Black	Read	411575018
9S44	Black - other African country	Black	Read	285943014
9S45	Black E Afric Asia/Indo-Caribb	Mixed	Read	514651000006112
9S45-1	Black East African Asian	Mixed	Read	411576017
9S45-2	Black Indo-Caribbean	Mixed	Read	411577014
9S46	Black Indian sub-continent	Black	Read	285948017
9S47	Black - other Asian	Mixed	Read	285949013
9S48	Black Black - other	Black	Read	285950013
9S5	Black - other, mixed	Black	Read	285951012
9S51	Other Black - Black/White orig	Mixed	Read	285952017
9S52	Other Black - Black/Asian orig	Mixed	Read	285953010
9S6	Indian	Asian	Read	285954016
9S7	Pakistani	Asian	Read	285955015
9S8	Bangladeshi	Asian	Read	285956019
9S9	Chinese	Asian	Read	56590016
9SA	Other ethnic non-mixed (NMO)	Other	Read	285958018
9SA1	Brit. ethnic minor. spec.(NMO)	Other	Read	285959014
9SA2	Brit. ethnic minor. unsp (NMO)	Other	Read	285960016
9SA3	Caribbean I./W.I./Guyana (NMO)	Black	Read	405067016
9SA3-1	Caribbean Island (NMO)	Black	Read	411578016
9SA3-2	West Indian (NMO)	Black	Read	411579012
9SA3-3	Guyana (NMO)	Black	Read	411580010
9SA4	N African Arab/Iranian (NMO)	Other	Read	405068014
9SA4-1	North African Arab (NMO)	Other	Read	411581014
9t17	Asian/Asian British: Pakistani - NI ethnic cat 2011 census	Asian	Read	1968311000006115

Code	Term	Group	CodingSystem	medcodeid
9T17	Niuean	Other	Read	1968311000006115
9t18	Asian/Asian British: Bangladeshi - NI ethnic cat 2011 census	Asian	Read	286015010
9T18	Tokelauan	Other	Read	286015010
9t19	Asian/Asian British: Chinese - NI ethnic cat 2011 census	Asian	Read	501416013
9T19	Fijian	Other	Read	501416013
9t1A	Asian/Asian British: other Asian - NI ethnic cat 2011 census	Asian	Read	286017019
9T1A	Other Pacific ethnic group	Other	Read	286017019
9t1B	Black/Afri/Carib/Black Brit: African- NI eth cat 2011 census	Black	Read	286018012
9T1B	South East Asian	Asian	Read	286018012
1348	Middle Eastern origin	Other	Read	250229015
1349	Far Eastern origin	Asian	Read	250230013
2261	O/E - Europeanoid	White	Read	253629014
2262	O/E - Negroid	Black	Read	253630016
2263	O/E - Mongoloid origin	Asian	Read	402434017
2263-1	O/E - Asian origin	Asian	Read	412016016
2264	O/E - Australoid	Other	Read	253634013
134A	West Indian origin	Black	Read	250231012
134B	RACE: Caucasian	White	Read	196641000006110
134C	RACE: Arab	Other	Read	196621000006115
134D	RACE: Chinese	Asian	Read	196651000006112
134E	RACE: Japanese	Asian	Read	196661000006114
134F	RACE: Korean	Asian	Read	196671000006119
134G	RACE: Oriental	Asian	Read	196701000006118
134H	RACE: Afro-caribbean	Black	Read	196601000006113
134I	RACE: Bangladeshi	Asian	Read	196631000006117

Code	Term	Group	CodingSystem	medcodeid
134J	RACE: Mixed	Mixed	Read	196681000006116
134K	Race: West indian	Black	Read	250243013
134L	RACE: Afro-caucasian	Mixed	Read	196611000006111
134M	RACE: Pakistani	Asian	Read	196721000006111
134N	Race: White	White	Read	2537217015
134O	RACE: Unknown	Other	Read	196731000006114
134P	RACE: Not stated	Not recorded	Read	196691000006118
134P-1	Race: Other	Other	Read	371005013
226Z	O/E - ethnic group NOS	Other	Read	253635014
9i0	British or mixed British - ethnic category 2001 census	White	Read	158341000000117
9i00	White British - ethnic category 2001 census	White	Read	1063981000000117
9i1	Irish - ethnic category 2001 census	White	Read	141301000000110
9i10	White Irish - ethnic category 2001 census	White	Read	1064041000000111
9i2	Other White background - ethnic category 2001 census	White	Read	141311000000112
9i20	English - ethnic category 2001 census	White	Read	157281000000117
9i21	Scottish - ethnic category 2001 census	White	Read	141431000000111
9i22	Welsh - ethnic category 2001 census	White	Read	141441000000119
9i23	Cornish - ethnic category 2001 census	White	Read	141461000000118
9i24	Northern Irish - ethnic category 2001 census	White	Read	141451000000116
9i25	Ulster Scots - ethnic category 2001 census	White	Read	142691000000116
9i26	Cypriot (part not stated) - ethnic category 2001 census	White	Read	141661000000115
9i27	Greek - ethnic category 2001 census	White	Read	142701000000116
9i28	Greek Cypriot - ethnic category 2001 census	White	Read	142711000000119
9i29	Turkish - ethnic category 2001 census	White	Read	156921000000110
9i2A	Turkish Cypriot - ethnic category 2001 census	White	Read	142721000000113

Code	Term	Group	CodingSystem	medcodeid
9i2B	Italian - ethnic category 2001 census	White	Read	158481000000115
9i2C	Irish Traveller - ethnic category 2001 census	White	Read	138171000000114
9i2D	Traveller - ethnic category 2001 census	White	Read	138181000000111
9i2E	Gypsy/Romany - ethnic category 2001 census	White	Read	138191000000113
9i2F	Polish - ethnic category 2001 census	White	Read	138201000000110
9i2G	Baltic Estonian/Latvian/Lithuanian - ethn categ 2001 census	White	Read	937301000006110
9i2H	Commonwealth (Russian) Indep States - ethn categ 2001 census	White	Read	937311000006113
9i2J	Kosovan - ethnic category 2001 census	White	Read	142741000000118
9i2K	Albanian - ethnic category 2001 census	White	Read	138231000000116
9i2L	Bosnian - ethnic category 2001 census	White	Read	142751000000115
9i2M	Croatian - ethnic category 2001 census	White	Read	142761000000117
9i2N	Serbian - ethnic category 2001 census	White	Read	157991000000110
9i2P	Other republics former Yugoslavia - ethnic categ 2001 census	White	Read	937371000006116
9i2Q	Mixed Irish and other White - ethnic category 2001 census	White	Read	142781000000114
9i2R	Oth White European/European unsp/Mixed European 2001 census	White	Read	937391000006115
9i2S	Other mixed White - ethnic category 2001 census	White	Read	142791000000111
9i2T	Other White or White unspecified ethnic category 2001 census	White	Read	937411000006115
9i3	White and Black Caribbean - ethnic category 2001 census	Mixed	Read	141321000000118
9i4	White and Black African - ethnic category 2001 census	Mixed	Read	141331000000116
9i5	White and Asian - ethnic category 2001 census	Mixed	Read	141341000000113

Code	Term	Group	CodingSystem	medcodeid
9i6	Other Mixed background - ethnic category 2001 census	Mixed	Read	141351000000111
9i60	Black and Asian - ethnic category 2001 census	Mixed	Read	141471000000113
9i61	Black and Chinese - ethnic category 2001 census	Mixed	Read	141481000000110
9i62	Black and White - ethnic category 2001 census	Mixed	Read	157291000000115
9i63	Chinese and White - ethnic category 2001 census	Mixed	Read	141491000000112
9i64	Asian and Chinese - ethnic category 2001 census	Asian	Read	158361000000116
9i65	Other Mixed or Mixed unspecified ethnic category 2001 census	Mixed	Read	937511000006119
9i7	Indian or British Indian - ethnic category 2001 census	Asian	Read	157271000000119
9i8	Pakistani or British Pakistani - ethnic category 2001 census	Asian	Read	141361000000114
9i9	Bangladeshi or British Bangladeshi - ethn categ 2001 census	Asian	Read	937541000006115
ESCTBL5	Black - ethnic group	Black	EMIS	459730016
ESCTMI5	Mixed ethnic census group	Mixed	EMIS	459729014
ESCTBL5-1	Black	Black	EMIS	459731017

Table 5: BMIover30_SNOMED

Cluster_ID	Cluster_description	SNOMED_code	SNOMED_code_description	PCD_Refset_ID	Service_and_Ruleset
BMI30_COD	Body mass index (BMI) codes >= 30 without an associated BMI value	162690006	On examination - obese (finding)	999011051000230106	PLE GDPPR | QOF Obesity
BMI30_COD	Body mass index (BMI) codes >= 30 without an associated BMI value	162864005	Body mass index 30+ - obesity (finding)	999011051000230106	PLE GDPPR | QOF Obesity

Cluster_ID	Cluster_description	SNOMED_code	SNOMED_code_description	PCD_Refset_ID	Service_and_Ruleset
BMI30_COD	Body mass index (BMI) codes >= 30 without an associated BMI value	408512008	Body mass index 40+ - severely obese (finding)	999011051000230106	PLE GDPPR | QOF Obesity
BMI30_COD	Body mass index (BMI) codes >= 30 without an associated BMI value	414915002	Obese (finding)	999011051000230106	PLE GDPPR | QOF Obesity
BMI30_COD	Body mass index (BMI) codes >= 30 without an associated BMI value	443371000124107	Obese class I (finding)	999011051000230106	PLE GDPPR | QOF Obesity
BMI30_COD	Body mass index (BMI) codes >= 30 without an associated BMI value	443381000124105	Obese class II (finding)	999011051000230106	PLE GDPPR | QOF Obesity
BMI30_COD	Body mass index (BMI) codes >= 30 without an associated BMI value	914721000000105	Obese class I (body mass index 30.0 - 34.9) (finding)	999011051000230106	PLE GDPPR | QOF Obesity
BMI30_COD	Body mass index (BMI) codes >= 30 without an associated BMI value	914731000000107	Obese class II (body mass index 35.0 - 39.9) (finding)	999011051000230106	PLE GDPPR | QOF Obesity
BMI30_COD	Body mass index (BMI) codes >= 30 without an associated BMI value	914741000000103	Obese class III (body mass index equal to or greater than 40.0) (finding)	999011051000230106	PLE GDPPR | QOF Obesity

Table 6: CurrentSmoker_SNOMED

Cluster_ID	Cluster_description	SNOMED_code	SNOMED_code_description	PCD_Refset_ID	Service_and_Ruleset
LSMOK_COD	Smoker codes	134406006	Smoking reduced (finding)	999004211000230104	INLIQ Smoking | PLE GDPPR | QOF Smoking
LSMOK_COD	Smoker codes	160603005	Light cigarette smoker (1-9 cigs/day) (finding)	999004211000230104	INLIQ Smoking | PLE GDPPR | QOF Smoking

Cluster_ID	Cluster_description	SNOMED_code	SNOMED_code_description	PCD_Refset_ID	Service_and_Ruleset
LSMOK_COD	Smoker codes	160604004	Moderate cigarette smoker (10-19 cigs/day) (finding)	999004211000230104	INLIQ Smoking | PLE GDPPR | QOF Smoking
LSMOK_COD	Smoker codes	160605003	Heavy cigarette smoker (20-39 cigs/day) (finding)	999004211000230104	INLIQ Smoking | PLE GDPPR | QOF Smoking
LSMOK_COD	Smoker codes	160606002	Very heavy cigarette smoker (40+ cigs/day) (finding)	999004211000230104	INLIQ Smoking | PLE GDPPR | QOF Smoking
LSMOK_COD	Smoker codes	160612007	Keeps trying to stop smoking (finding)	999004211000230104	INLIQ Smoking | PLE GDPPR | QOF Smoking
LSMOK_COD	Smoker codes	160613002	Admitted tobacco consumption possibly untrue (finding)	999004211000230104	INLIQ Smoking | PLE GDPPR | QOF Smoking
LSMOK_COD	Smoker codes	160616005	Trying to give up smoking (finding)	999004211000230104	INLIQ Smoking | PLE GDPPR | QOF Smoking
LSMOK_COD	Smoker codes	160619003	Rolls own cigarettes (finding)	999004211000230104	INLIQ Smoking | PLE GDPPR | QOF Smoking
LSMOK_COD	Smoker codes	203191000000107	Wants to stop smoking (finding)	999004211000230104	INLIQ Smoking | PLE GDPPR | QOF Smoking
LSMOK_COD	Smoker codes	225934006	Smokes in bed (finding)	999004211000230104	INLIQ Smoking | PLE GDPPR | QOF Smoking
LSMOK_COD	Smoker codes	230056004	Cigarette consumption (observable entity)	999004211000230104	INLIQ Smoking | PLE GDPPR | QOF Smoking

Cluster_ID	Cluster_description	SNOMED_code	SNOMED_code_description	PCD_Refset_ID	Service_and_Ruleset
LSMOK_COD	Smoker codes	230057008	Cigar consumption (observable entity)	999004211000230104	INLIQ Smoking | PLE GDPPR | QOF Smoking
LSMOK_COD	Smoker codes	230058003	Pipe tobacco consumption (observable entity)	999004211000230104	INLIQ Smoking | PLE GDPPR | QOF Smoking
LSMOK_COD	Smoker codes	230059006	Occasional cigarette smoker (finding)	999004211000230104	INLIQ Smoking | PLE GDPPR | QOF Smoking
LSMOK_COD	Smoker codes	230060001	Light cigarette smoker (finding)	999004211000230104	INLIQ Smoking | PLE GDPPR | QOF Smoking
LSMOK_COD	Smoker codes	230062009	Moderate cigarette smoker (finding)	999004211000230104	INLIQ Smoking | PLE GDPPR | QOF Smoking
LSMOK_COD	Smoker codes	230063004	Heavy cigarette smoker (finding)	999004211000230104	INLIQ Smoking | PLE GDPPR | QOF Smoking
LSMOK_COD	Smoker codes	230064005	Very heavy cigarette smoker (finding)	999004211000230104	INLIQ Smoking | PLE GDPPR | QOF Smoking
LSMOK_COD	Smoker codes	230065006	Chain smoker (finding)	999004211000230104	INLIQ Smoking | PLE GDPPR | QOF Smoking
LSMOK_COD	Smoker codes	266918002	Tobacco smoking consumption (observable entity)	999004211000230104	INLIQ Smoking | PLE GDPPR | QOF Smoking
LSMOK_COD	Smoker codes	266920004	Trivial cigarette smoker (less than one cigarette/day) (finding)	999004211000230104	INLIQ Smoking | PLE GDPPR | QOF Smoking

Cluster_ID	Cluster_description	SNOMED_code	SNOMED_code_description	PCD_Refset_ID	Service_and_Ruleset
LSMOK_COD	Smoker codes	266929003	Smoking started (finding)	999004211000230104	INLIQ Smoking | PLE GDPPR | QOF Smoking
LSMOK_COD	Smoker codes	308438006	Smoking restarted (finding)	999004211000230104	INLIQ Smoking | PLE GDPPR | QOF Smoking
LSMOK_COD	Smoker codes	394871007	Thinking about stopping smoking (finding)	999004211000230104	INLIQ Smoking | PLE GDPPR | QOF Smoking
LSMOK_COD	Smoker codes	394872000	Ready to stop smoking (finding)	999004211000230104	INLIQ Smoking | PLE GDPPR | QOF Smoking
LSMOK_COD	Smoker codes	394873005	Not interested in stopping smoking (finding)	999004211000230104	INLIQ Smoking | PLE GDPPR | QOF Smoking
LSMOK_COD	Smoker codes	401159003	Reason for restarting smoking (observable entity)	999004211000230104	INLIQ Smoking | PLE GDPPR | QOF Smoking
LSMOK_COD	Smoker codes	413173009	Minutes from waking to first tobacco consumption (observable entity)	999004211000230104	INLIQ Smoking | PLE GDPPR | QOF Smoking
LSMOK_COD	Smoker codes	428041000124106	Occasional tobacco smoker (finding)	999004211000230104	INLIQ Smoking | PLE GDPPR | QOF Smoking
LSMOK_COD	Smoker codes	446172000	Failed attempt to stop smoking (finding)	999004211000230104	INLIQ Smoking | PLE GDPPR | QOF Smoking
LSMOK_COD	Smoker codes	449868002	Smokes tobacco daily (finding)	999004211000230104	INLIQ Smoking | PLE GDPPR | QOF Smoking

Cluster_ID	Cluster_description	SNOMED_code	SNOMED_code_description	PCD_Refset_ID	Service_and_Ruleset
LSMOK_COD	Smoker codes	56578002	Moderate smoker (20 or less per day) (finding)	999004211000230104	INLIQ Smoking | PLE GDPPR | QOF Smoking
LSMOK_COD	Smoker codes	56771006	Heavy smoker (over 20 per day) (finding)	999004211000230104	INLIQ Smoking | PLE GDPPR | QOF Smoking
LSMOK_COD	Smoker codes	59978006	Cigar smoker (finding)	999004211000230104	INLIQ Smoking | PLE GDPPR | QOF Smoking
LSMOK_COD	Smoker codes	65568007	Cigarette smoker (finding)	999004211000230104	INLIQ Smoking | PLE GDPPR | QOF Smoking
LSMOK_COD	Smoker codes	77176002	Smoker (finding)	999004211000230104	INLIQ Smoking | PLE GDPPR | QOF Smoking
LSMOK_COD	Smoker codes	82302008	Pipe smoker (finding)	999004211000230104	INLIQ Smoking | PLE GDPPR | QOF Smoking
LSMOK_COD	Smoker codes	836001000000109	Waterpipe tobacco consumption (observable entity)	999004211000230104	INLIQ Smoking | PLE GDPPR | QOF Smoking
