# Supplementary material for: Inequalities in cancer screening participation between adults with and without severe mental illness: results from a cross-sectional analysis of primary care data on English Screening Programmes
Source: Br J Cancer. 2023 May 4;129(1):81–93. doi: 10.1038/s41416-023-02249-3 (PMC10307861; doi:10.1038/s41416-023-02249-3)
Supplement: Supplementary file 2 — Appendix 2 [file 41416_2023_2249_MOESM2_ESM.docx]

Appendix 2: SM Idiagnosis_SNOMED_Read_EMIS

| Code | Term | Group | ParentGroup | CodingSystem | medcodeid |
| --- | --- | --- | --- | --- | --- |
| E1005 | Schizophrenia in remission | SCH | SMI | Read | 9225016 |
| E1032 | Chronic paranoid schizophrenia | SCH | SMI | Read | 52897013 |
| E1022 | Chronic catatonic schizophrenia | SCH | SMI | Read | 114616017 |
| E1001 | Subchronic schizophrenia | SCH | SMI | Read | 28758018 |
| E1021 | Subchronic catatonic schizophrenia | SCH | SMI | Read | 71539017 |
| E13y1 | Brief reactive psychosis | PSY | SMI | Read | 10122017 |
| E1031 | Subchronic paranoid schizophrenia | SCH | SMI | Read | 132503015 |
| E1013 | Acute exacerbation of subchronic hebephrenic schizophrenia | SCH | SMI | Read | 294738017 |
| E111 | Recurrent manic episodes | BPD | SMI | Read | 294810017 |
| E115z | Bipolar affective disorder, currently depressed, NOS | BPD | SMI | Read | 294868016 |
| E120 | Simple paranoid state | PSY | SMI | Read | 294904016 |
| E13y | Other reactive psychoses | PSY | SMI | Read | 294926015 |
| E1014 | Acute exacerbation of chronic hebephrenic schizophrenia | SCH | SMI | Read | 294739013 |
| E102 | Catatonic schizophrenia | SCH | SMI | Read | 294742019 |
| E1023 | Acute exacerbation of subchronic catatonic schizophrenia | SCH | SMI | Read | 294750011 |
| E10yz | Other schizophrenia NOS | SCH | SMI | Read | 294789017 |
| E114z | Bipolar affective disorder, currently manic, NOS | BPD | SMI | Read | 294858014 |
| E116 | Mixed bipolar affective disorder | BPD | SMI | Read | 294869012 |
| E123-1 | Folie a deux | PSY | SMI | Read | 294908018 |
| E100z | Simple schizophrenia NOS | SCH | SMI | Read | 294734015 |
| E1106 | Single manic episode in full remission | BPD | SMI | Read | 294808019 |
| E12z | Paranoid psychosis NOS | PSY | SMI | Read | 294913019 |
| E134 | Psychogenic paranoid psychosis | PSY | SMI | Read | 294924017 |
| E100-1 | Schizophrenia simplex | SCH | SMI | Read | 294726014 |
| E1103 | Single manic episode, severe without mention of psychosis | BPD | SMI | Read | 294805016 |
| E1104 | Single manic episode, severe, with psychosis | BPD | SMI | Read | 294806015 |
| E1142 | Bipolar affective disorder, currently manic, moderate | BPD | SMI | Read | 294850019 |
| E1055 | Latent schizophrenia in remission | PSY | SMI | Read | 294770017 |
| E1161 | Mixed bipolar affective disorder, mild | BPD | SMI | Read | 294871012 |
| E101z | Hebephrenic schizophrenia NOS | SCH | SMI | Read | 294741014 |
| E1024 | Acute exacerbation of chronic catatonic schizophrenia | SCH | SMI | Read | 294751010 |
| E1102 | Single manic episode, moderate | BPD | SMI | Read | 294804017 |
| E12y | Other paranoid states | PSY | SMI | Read | 294910016 |
| E1100 | Single manic episode, unspecified | BPD | SMI | Read | 294802018 |
| E1160 | Mixed bipolar affective disorder, unspecified | BPD | SMI | Read | 294870013 |
| E1162 | Mixed bipolar affective disorder, moderate | BPD | SMI | Read | 294872017 |
| Eu23y | [X]Other acute and transient psychotic disorders | PSY | SMI | Read | 296083012 |
| E114-1 | Manic-depressive - now manic | BPD | SMI | Read | 294847017 |
| E1140 | Bipolar affective disorder, currently manic, unspecified | BPD | SMI | Read | 294848010 |
| E11yz | Other and unspecified manic-depressive psychoses NOS | BPD | SMI | Read | 294896010 |
| Eu22z | [X]Persistent delusional disorder, unspecified | PSY | SMI | Read | 296066015 |
| E10y-1 | Cenesthopathic schizophrenia | SCH | SMI | Read | 294788013 |
| E1172 | Unspecified bipolar affective disorder, moderate | BPD | SMI | Read | 294883014 |
| E1110 | Recurrent manic episodes, unspecified | BPD | SMI | Read | 294811018 |
| E1151 | Bipolar affective disorder, currently depressed, mild | BPD | SMI | Read | 294862015 |
| E11y3 | Other mixed manic-depressive psychoses | BPD | SMI | Read | 294895014 |
| Eu20z | [X]Schizophrenia, unspecified | SCH | SMI | Read | 296040018 |
| E1054 | Acute exacerbation of chronic latent schizophrenia | PSY | SMI | Read | 294769018 |
| E1052 | Chronic latent schizophrenia | PSY | SMI | Read | 294767016 |
| E1176 | Unspecified bipolar affective disorder, in full remission | BPD | SMI | Read | 294887010 |
| E11y1 | Atypical manic disorder | BPD | SMI | Read | 294893019 |
| Eu25z | [X]Schizoaffective disorder, unspecified | PSY | SMI | Read | 296096013 |
| E102z | Catatonic schizophrenia NOS | SCH | SMI | Read | 294753013 |
| E105 | Latent schizophrenia | PSY | SMI | Read | 294764011 |
| E1166 | Mixed bipolar affective disorder, in full remission | BPD | SMI | Read | 294876019 |
| E117 | Unspecified bipolar affective disorder | BPD | SMI | Read | 294880012 |
| E1171 | Unspecified bipolar affective disorder, mild | BPD | SMI | Read | 294882016 |
| E11z0 | Unspecified affective psychoses NOS | BPD | SMI | Read | 294898011 |
| E10 | Schizophrenic disorders | SCH | SMI | Read | 294725013 |
| E100 | Simple schizophrenia | SCH | SMI | Read | 294727017 |
| E1012 | Chronic hebephrenic schizophrenia | SCH | SMI | Read | 294737010 |
| E116z | Mixed bipolar affective disorder, NOS | BPD | SMI | Read | 294877011 |
| E11z | Other and unspecified affective psychoses | BPD | SMI | Read | 294897018 |
| Eu31z | [X]Bipolar affective disorder, unspecified | BPD | SMI | Read | 296135011 |
| E1000 | Unspecified schizophrenia | SCH | SMI | Read | 294728010 |
| E1015 | Hebephrenic schizophrenia in remission | SCH | SMI | Read | 294740010 |
| E10z | Schizophrenia NOS | SCH | SMI | Read | 294790014 |
| E1170 | Unspecified bipolar affective disorder, unspecified | BPD | SMI | Read | 294881011 |
| E131 | Acute hysterical psychosis | PSY | SMI | Read | 294919015 |
| E105z | Latent schizophrenia NOS | PSY | SMI | Read | 294771018 |
| E121-1 | Sanders disease | PSY | SMI | Read | 161371000006118 |
| Eu233 | [X]Other acute predominantly delusional psychotic disorders | PSY | SMI | Read | 401859016 |
| Eu31y | [X]Other bipolar affective disorders | BPD | SMI | Read | 401865016 |
| E1115 | Recurrent manic episodes, partial or unspecified remission | BPD | SMI | Read | 182861000006110 |
| Eu30z | [X]Manic episode, unspecified | BPD | SMI | Read | 401864017 |
| Eu220-5 | [X]Paranoia | PSY | SMI | Read | 215871000000115 |
| E107z | Schizo-affective schizophrenia NOS | SCH | SMI | Read | 155161000006117 |
| Eu220-1 | [X]Paranoid psychosis | PSY | SMI | Read | 215851000000112 |
| 286-1 | Poor insight into psychotic condition | PSY | SMI | Read | 215691000006112 |
| Eu21 | [X]Schizotypal disorder | PSY | SMI | Read | 401856011 |
| E110 | Manic disorder, single episode | BPD | SMI | Read | 401765010 |
| E1113 | Recurrent manic episodes, severe without mention psychosis | BPD | SMI | Read | 182871000006115 |
| E10y | Other schizophrenia | SCH | SMI | Read | 401764014 |
| E1z | Non-organic psychosis NOS | PSY | SMI | Read | 401771016 |
| ZV110 | [V]Personal history of schizophrenia | SCH | SMI | Read | 460273017 |
| Eu232 | [X]Acute schizophrenia-like psychotic disorder | PSY | SMI | Read | 362421000006113 |
| Eu2z | [X]Unspecified nonorganic psychosis | PSY | SMI | Read | 401862018 |
| E12 | Paranoid states | PSY | SMI | Read | 243361000006117 |
| E104 | Acute schizophrenic episode | SCH | SMI | Read | 401763015 |
| E1175 | Unspecified bipolar affect disord, partial/unspec remission | BPD | SMI | Read | 82091000006116 |
| ZV111-2 | [V]Personal history of manic-depressive psychosis | BPD | SMI | Read | 1227584015 |
| Eu230-1 | [X]Bouffee delirante | PSY | SMI | Read | 367961000006110 |
| Eu231-1 | [X]Bouffee delirante with symptoms of schizophrenia | PSY | SMI | Read | 367971000006115 |
| Eu31 | [X]Bipolar affective disorder | BPD | SMI | Read | 367101000006112 |
| Eu316 | [X]Bipolar affective disorder, current episode mixed | BPD | SMI | Read | 367121000006119 |
| Eu312 | [X]Bipolar affect disorder cur epi manic with psychotic symp | BPD | SMI | Read | 367071000006119 |
| Eu202 | [X]Catatonic schizophrenia | SCH | SMI | Read | 370451000006110 |
| Eu21-3 | [X]Latent schizophrenia | PSY | SMI | Read | 395021000006111 |
| E114 | Bipolar affective disorder, currently manic | BPD | SMI | Read | 513801000006112 |
| Eu24-1 | [X]Folie a deux | PSY | SMI | Read | 387071000006117 |
| E1155 | Bipolar affect disord, now depressed, part/unspec remission | BPD | SMI | Read | 513711000006118 |
| E133-1 | Bouffee delirante | PSY | SMI | Read | 523481000006114 |
| Eu31y-1 | [X]Bipolar II disorder | BPD | SMI | Read | 367161000006113 |
| Eu20y-1 | [X]Cenesthopathic schizophrenia | SCH | SMI | Read | 370631000006117 |
| E1144 | Bipolar affect disord, currently manic,severe with psychosis | BPD | SMI | Read | 513701000006116 |
| 1S42 | Manic mood | BPD | SMI | Read | 2157096015 |
| E1072 | Chronic schizo-affective schizophrenia | SCH | SMI | Read | 556631000006116 |
| E1070 | Unspecified schizo-affective schizophrenia | SCH | SMI | Read | 78531000006116 |
| Eu315 | [X]Bipolar affect dis cur epi severe depres with psyc symp | BPD | SMI | Read | 367061000006114 |
| Eu220-3 | [X]Paraphrenia - late | PSY | SMI | Read | 418251000006110 |
| Eu31y-2 | [X]Recurrent manic episodes | BPD | SMI | Read | 424661000006111 |
| Eu202-2 | [X]Schizophrenic catalepsy | SCH | SMI | Read | 425641000006111 |
| Eu20y-3 | [X]Schizophrenifrm psychos NOS | SCH | SMI | Read | 425711000006116 |
| Eu300 | [X]Hypomania | BPD | SMI | Read | 389401000006111 |
| Eu250-1 | [X]Schizoaffective psychosis, manic type | PSY | SMI | Read | 425571000006119 |
| Eu21-7 | [X]Pseudopsychopathic schizophrenia | PSY | SMI | Read | 423741000006116 |
| Eu251-2 | [X]Schizophreniform psychosis, depressive type | PSY | SMI | Read | 425691000006119 |
| Eu205 | [X]Residual schizophrenia | SCH | SMI | Read | 424841000006113 |
| Eu202-3 | [X]Schizophrenic catatonia | SCH | SMI | Read | 425651000006113 |
| Eu22 | [X]Persistent delusional disorders | PSY | SMI | Read | 419861000006117 |
| Eu25z-1 | [X]Schizoaffective psychosis NOS | PSY | SMI | Read | 425551000006112 |
| Eu251-1 | [X]Schizoaffective psychosis, depressive type | PSY | SMI | Read | 425561000006114 |
| Eu233-2 | [X]Psychogenic paranoid psychosis | PSY | SMI | Read | 424071000006112 |
| E13z | Nonorganic psychosis NOS | PSY | SMI | Read | 223611000000117 |
| Eu200 | [X]Paranoid schizophrenia | SCH | SMI | Read | 418221000006118 |
| Eu30 | [X]Manic episode | BPD | SMI | Read | 396741000006113 |
| Eu3y0-1 | [X]Mixed affective episode | BPD | SMI | Read | 398541000006116 |
| Eu30z-1 | [X]Mania NOS | BPD | SMI | Read | 396691000006111 |
| E101 | Hebephrenic schizophrenia | SCH | SMI | Read | 819351000006115 |
| Eu21-6 | [X]Pseudoneurotic schizophrenia | PSY | SMI | Read | 423731000006114 |
| Eu232-4 | [X]Schizophrenic reaction | SCH | SMI | Read | 425671000006115 |
| Eu220-4 | [X]Sensitiver Beziehungswahn | PSY | SMI | Read | 425921000006114 |
| Eu311 | [X]Bipolar affect disorder cur epi manic wout psychotic symp | BPD | SMI | Read | 367081000006116 |
| E107-99 | Acute schizo affective psychosis | PSY | SMI | Read | 882301000006112 |
| Eu24 | [X]Induced delusional disorder | PSY | SMI | Read | 389641000006110 |
| E11-99 | Manic-depressive psychoses | BPD | SMI | Read | 882311000006110 |
| Eu24-3 | [X]Induced psychotic disorder | PSY | SMI | Read | 389661000006114 |
| E11 | Affective psychoses | BPD | SMI | Read | 473201000006114 |
| ZV111-1 | [V]Personal history of manic-depressive psychosis | BPD | SMI | Read | 345141000006111 |
| Eu310 | [X]Bipolar affective disorder, current episode hypomanic | BPD | SMI | Read | 367111000006110 |
| Eu231 | [X]Acute polymorphic psychot disord with symp of schizophren | PSY | SMI | Read | 362381000006115 |
| Eu230 | [X]Acute polymorphic psychot disord without symp of schizoph | PSY | SMI | Read | 362391000006117 |
| Eu221 | [X]Delusional misidentification syndrome | PSY | SMI | Read | 914471000006113 |
| Eu332-3 | [X]Manic-depress psychosis,depressd,no psychotic symptoms | BPD | SMI | Read | 396771000006117 |
| Eu302-3 | [X]Manic stupor | BPD | SMI | Read | 396761000006112 |
| E1146 | Bipolar affective disorder, currently manic, full remission | BPD | SMI | Read | 513811000006110 |
| Eu20 | [X]Schizophrenia | SCH | SMI | Read | 425601000006114 |
| Eu21-8 | [X]Schizotypal personality disorder | PSY | SMI | Read | 425731000006110 |
| Eu319-1 | [X]Bipolar II disorder | BPD | SMI | Read | 1785871000006117 |
| Eu250 | [X]Schizoaffective disorder, manic type | PSY | SMI | Read | 425511000006111 |
| Eu223 | [X]Paranoid state in remission | PSY | SMI | Read | 1667581000000114 |
| Eu23z-2 | [X]Reactive psychosis | PSY | SMI | Read | 424511000006112 |
| Eu2z-1 | [X]Psychosis NOS | PSY | SMI | Read | 424231000006116 |
| Eu250-2 | [X]Schizophreniform psychosis, manic type | PSY | SMI | Read | 425701000006119 |
| Eu318 | [X]Bipolar affective disorder type I | BPD | SMI | Read | 1785851000006110 |
| E1074 | Acute exacerbation of chronic schizo-affective schizophrenia | SCH | SMI | Read | 456731000006115 |
| EMISICD10\|F2098 | Schizophrenia, unspecified, other | SCH | SMI | EMIS | 1976961000006110 |
| E1165 | Mixed bipolar affective disorder, partial/unspec remission | BPD | SMI | Read | 701051000006118 |
| ^ESCTBO352777 | BouffÃ©e dÃ©lirante | PSY | SMI | EMIS | 3527771000006116 |
| ^ESCTBR258680 | Brief psychotic disorder | PSY | SMI | EMIS | 2586801000006115 |
| EMISICD10\|F2018 | Hebephrenic schizophrenia, other | SCH | SMI | EMIS | 1976891000006110 |
| ^ESCTSU272626 | Subchronic disorganised schizophrenia with acute exacerbations | SCH | SMI | EMIS | 2726261000006113 |
| ^ESCTFO350478 | Folie E deux | PSY | SMI | EMIS | 3504781000006118 |
| ^ESCTIN350475 | Induced psychosis | PSY | SMI | EMIS | 3504751000006114 |
| ^ESCTDI300560 | Disorganized schizophrenia in remission | SCH | SMI | EMIS | 3005601000006118 |
| ^ESCTSH350474 | Shared psychotic disorder | PSY | SMI | EMIS | 3504741000006112 |
| ^ESCTSE349764 | Severe bipolar I disorder, most recent episode depressed without psychotic features | BPD | SMI | EMIS | 3497641000006117 |
| ^ESCTSU293896 | Subchronic disorganized schizophrenia | SCH | SMI | EMIS | 2938961000006110 |
| EMISCDE13 | Delusions | PSY | SMI | EMIS | 981141000006111 |
| ^ESCTBI476731 | Bipolar affective disorder, current episode mixed | BPD | SMI | EMIS | 4767311000006117 |
| ^ESCTDI306812 | Disorganised schizophrenia | SCH | SMI | EMIS | 3068121000006112 |
| E10-99 | Schizophrenic psychoses | SCH | SMI | Read | 882291000006111 |
| ^ESCTSC524896 | Schizophrenic prodrome | SCH | SMI | EMIS | 5248961000006113 |
| ^ESCTDI300561 | Disorganized schizophrenia, in remission | SCH | SMI | EMIS | 3005611000006115 |
| ^ESCTIN311671 | Involutional paraphrenia | PSY | SMI | EMIS | 3116711000006110 |
| ^ESCTDE564562 | De Clerambaults syndrome | PSY | SMI | EMIS | 5645621000006116 |
| ^ESCTAC476391 | Acute exacerbation of subchronic schizoaffective schizophrenia | SCH | SMI | EMIS | 4763911000006114 |
| ^ESCTCH476375 | Chronic catatonic schizophrenia with acute exacerbation | SCH | SMI | EMIS | 4763751000006112 |
| ^ESCTSC476369 | Schizophrenia, catatonic | SCH | SMI | EMIS | 4763691000006113 |
| ^ESCTNO823254 | Non-organic psychosis in remission | PSY | SMI | EMIS | 8232541000006114 |
| ^ESCTCH270556 | Chronic disorganised schizophrenia | SCH | SMI | EMIS | 2705561000006112 |
| ^ESCTCH270557 | Chronic disorganized schizophrenia | SCH | SMI | EMIS | 2705571000006117 |
| ^ESCTSE250101 | Severe bipolar I disorder, most recent episode manic, without psychotic features | BPD | SMI | EMIS | 2501011000006119 |
| ^ESCTMI823516 | Mixed bipolar affective disorder, in partial remission | BPD | SMI | EMIS | 8235161000006110 |
| ^ESCTSC257794 | Schizophrenia, in remission | SCH | SMI | EMIS | 2577941000006118 |
| ^ESCTIN350479 | Induced paranoid disorder | PSY | SMI | EMIS | 3504791000006115 |
| ^ESCTSU476389 | Subchronic schizoaffective schizophrenia | SCH | SMI | EMIS | 4763891000006112 |
| ^ESCTSU293895 | Subchronic disorganised schizophrenia | SCH | SMI | EMIS | 2938951000006113 |
| ^ESCTAC476364 | Acute exacerbation of chronic disorganised schizophrenia | SCH | SMI | EMIS | 4763641000006116 |
| ^ESCTSC387861 | Schizophreniform psychosis, depressive type | SCH | SMI | EMIS | 3878611000006116 |
| ^ESCTSU419615 | Subchronic schizophrenia with acute exacerbations | SCH | SMI | EMIS | 4196151000006111 |
| ^ESCTMI551797 | Mixed schizophrenic and affective psychosis | PSY | SMI | EMIS | 5517971000006118 |
| ^ESCTSC552457 | Schizophreniform psychosis, manic type | PSY | SMI | EMIS | 5524571000006112 |
| ^ESCTSC476370 | Schizophrenic flexibilatis cerea | SCH | SMI | EMIS | 4763701000006113 |
| ^ESCTSC419617 | Schizophrenia, catatonic, in remission | SCH | SMI | EMIS | 4196171000006118 |
| ^ESCTBI388629 | Bipolar disorder, in remission | BPD | SMI | EMIS | 3886291000006119 |
| ^ESCTPS362734 | Psychosis | PSY | SMI | EMIS | 3627341000006115 |
| ^ESCTBI635757 | Bipolar 1 disorder | BPD | SMI | EMIS | 6357571000006119 |
| ^ESCTMD271798 | MDI - Manic-depressive illness | BPD | SMI | EMIS | 2717981000006118 |
| ^ESCTRE291636 | Restzustand | SCH | SMI | EMIS | 2916361000006118 |
| ^ESCTSC300026 | Schizoptypal disorder | SCH | SMI | EMIS | 3000261000006112 |
| ^ESCTDI306813 | Disorganized schizophrenia | SCH | SMI | EMIS | 3068131000006110 |
| ^ESCTAC476366 | Acute exacerbation of chronic disorganized schizophrenia | SCH | SMI | EMIS | 4763661000006117 |
| ^ESCTBI352868 | Bipolar I disorder, most recent episode manic, in partial remission | BPD | SMI | EMIS | 3528681000006111 |
| 146H | H/O: psychosis | PSY | SMI | Read | 300411000000110 |
| Eu23z-1 | [X]Brief reactive psychosis NOS | PSY | SMI | Read | 367991000006119 |
| E1143 | Bipolar affect disord, currently manic, severe, no psychosis | BPD | SMI | Read | 513691000006116 |
| Eu252 | [X]Schizoaffective disorder, mixed type | PSY | SMI | Read | 425521000006115 |
| Eu22y | [X]Other persistent delusional disorders | PSY | SMI | Read | 401857019 |
| Eu23 | [X]Acute and transient psychotic disorders | PSY | SMI | Read | 362271000006110 |
| E1156 | Bipolar affective disorder, now depressed, in full remission | BPD | SMI | Read | 513861000006113 |
| E1002 | Chronic schizophrenic | SCH | SMI | Read | 1234861017 |
| Eu25 | [X]Schizoaffective disorders | PSY | SMI | Read | 425541000006110 |
| Eu22y-3 | [X]Paranoia querulans | PSY | SMI | Read | 418181000006110 |
| E1073 | Acute exacerbation subchronic schizo-affective schizophrenia | SCH | SMI | Read | 456801000006115 |
| E1025 | Catatonic schizophrenia in remission | SCH | SMI | Read | 178723016 |
| E1030 | Unspecified paranoid schizophrenia | SCH | SMI | Read | 294754019 |
| E1 | Non-organic psychoses | PSY | SMI | Read | 294724012 |
| EMISQPA1 | Paranoid | PSY | SMI | EMIS | 851701000006114 |
| E133 | Acute paranoid reaction | PSY | SMI | Read | 401770015 |
| E111z | Recurrent manic episode NOS | BPD | SMI | Read | 294818012 |
| Eu25y | [X]Other schizoaffective disorders | PSY | SMI | Read | 296095012 |
| Eu231-2 | [X]Cycloid psychosis with symptoms of schizophrenia | PSY | SMI | Read | 376281000006116 |
| Eu22y-2 | [X]Involutional paranoid state | PSY | SMI | Read | 394461000006110 |
| E12yz | Other paranoid states NOS | PSY | SMI | Read | 294912012 |
| EGTON118 | Obsessional compulsive psychosis | PSY | SMI | EMIS | 853201000006116 |
| E1y | Other specified non-organic psychoses | PSY | SMI | Read | 294949018 |
| Eu301 | [X]Mania without psychotic symptoms | BPD | SMI | Read | 296110017 |
| Eu252-1 | [X]Cyclic schizophrenia | PSY | SMI | Read | 376251000006112 |
| E11y0 | Unspecified manic-depressive psychoses | BPD | SMI | Read | 294892012 |
| E106 | Residual schizophrenia | SCH | SMI | Read | 43595011 |
| Eu30y | [X]Other manic episodes | BPD | SMI | Read | 296118012 |
| ^ESCTBI385440 | Bipolar 2 disorder | BPD | SMI | EMIS | 3854401000006113 |
| E1164 | Mixed bipolar affective disorder, severe, with psychosis | BPD | SMI | Read | 294874016 |
| ^ESCTSY350476 | Symbiotic psychosis | PSY | SMI | EMIS | 3504761000006111 |
| EMISICD10\|F2054 | Residual schizophrenia, incomplete remission | SCH | SMI | EMIS | 1975441000006110 |
| Eu31-2 | [X]Manic-depressive psychosis | BPD | SMI | Read | 396801000006115 |
| ^ESCTAC769626 | Acute polymorphic psychotic disorder with symptoms of schizophrenia | PSY | SMI | EMIS | 7696261000006113 |
| Eu31-1 | [X]Manic-depressive illness | BPD | SMI | Read | 396791000006116 |
| Eu206 | [X]Simple schizophrenia | SCH | SMI | Read | 426881000006111 |
| Eu313 | [X]Bipolar affect disorder cur epi mild or moderate depressn | BPD | SMI | Read | 367091000006118 |
| Eu221-1 | [X]Capgras syndrome | PSY | SMI | Read | 914461000006118 |
| Eu3z-1 | [X]Affective psychosis NOS | BPD | SMI | Read | 362781000006116 |
| ^ESCTSC476395 | Schizophrenia, schizoaffective, in remission | SCH | SMI | EMIS | 4763951000006110 |
| Eu302-2 | [X]Mania with mood-incongruent psychotic symptoms | BPD | SMI | Read | 396711000006114 |
| E1153 | Bipolar affect disord, now depressed, severe, no psychosis | BPD | SMI | Read | 513731000006112 |
| ^ESCTPA355573 | Paraphrenic schizophrenia | SCH | SMI | EMIS | 3555731000006114 |
| 146D | H/O: manic depressive disorder | BPD | SMI | Read | 1780205015 |
| E107 | Schizo-affective schizophrenia | SCH | SMI | Read | 155141000006116 |
| Eu252-2 | [X]Mixed schizophrenic and affective psychosis | PSY | SMI | Read | 398631000006113 |
| Eu24-2 | [X]Induced paranoid disorder | PSY | SMI | Read | 389651000006112 |
| Eu21-4 | [X]Prepsychotic schizophrenia | PSY | SMI | Read | 423271000006116 |
| E2122 | Schizotypal personality | PSY | SMI | Read | 155281000006119 |
| E122 | Paraphrenia | PSY | SMI | Read | 44335019 |
| Eu2y | [X]Other nonorganic psychotic disorders | PSY | SMI | Read | 412201000006113 |
| E10y0 | Atypical schizophrenia | SCH | SMI | Read | 1219653018 |
| Eu201-1 | [X]Disorganised schizophrenia | SCH | SMI | Read | 378051000006119 |
| ^ESCTPO502390 | Post-schizophrenic depression | SCH | SMI | EMIS | 5023901000006110 |
| Eu202-1 | [X]Catatonic stupor | SCH | SMI | Read | 370461000006112 |
| E115-1 | Manic-depressive - now depressed | BPD | SMI | Read | 294860011 |
| E12y0 | Paranoia querulans | PSY | SMI | Read | 294911017 |
| E13z-1 | Psychotic episode NOS | PSY | SMI | Read | 346895011 |
| E110-99 | Mania/hypomania | BPD | SMI | Read | 882321000006119 |
| E1101 | Single manic episode, mild | BPD | SMI | Read | 294803011 |
| E1034 | Acute exacerbation of chronic paranoid schizophrenia | SCH | SMI | Read | 294758016 |
| Eu205-1 | [X]Chronic undifferentiated schizophrenia | SCH | SMI | Read | 371031000006115 |
| Eu251 | [X]Schizoaffective disorder, depressive type | PSY | SMI | Read | 425501000006113 |
| E106-1 | Restzustand - schizophrenia | SCH | SMI | Read | 169061000006112 |
| Eu31-3 | [X]Manic-depressive reaction | BPD | SMI | Read | 396071000006119 |
| E1150 | Bipolar affective disorder, currently depressed, unspecified | BPD | SMI | Read | 294861010 |
| E1050 | Unspecified latent schizophrenia | PSY | SMI | Read | 294765012 |
| ^ESCTSC476394 | Schizoaffective schizophrenia, in remission | SCH | SMI | EMIS | 4763941000006113 |
| Eu317 | [X]Bipolar affective disorder, currently in remission | BPD | SMI | Read | 296130018 |
| ^ESCTFO350473 | Folie Ã  deux | PSY | SMI | EMIS | 3504731000006119 |
| ^ESCTSU476377 | Subchronic paranoid schizophrenia with acute exacerbation | SCH | SMI | EMIS | 4763771000006119 |
| Eu314 | [X]Bipol aff disord, curr epis sev depress, no psychot symp | BPD | SMI | Read | 367051000006112 |
| E1004 | Acute exacerbation of chronic schizophrenia | SCH | SMI | Read | 294731011 |
| E110z | Manic disorder, single episode NOS | BPD | SMI | Read | 294809010 |
| E12-99 | Paranoia | PSY | SMI | Read | 882331000006116 |
| Eu232-2 | [X]Brief schizophrenifrm psych | PSY | SMI | Read | 368011000006110 |
| E13 | Other nonorganic psychoses | PSY | SMI | Read | 25461000006115 |
| ^ESCTAC476392 | Acute exacerbation of chronic schizoaffective schizophrenia | SCH | SMI | EMIS | 4763921000006118 |
| E11y | Other and unspecified manic-depressive psychoses | BPD | SMI | Read | 294891017 |
| ^ESCTMA502396 | Manic | BPD | SMI | EMIS | 5023961000006111 |
| E104-1 | Oneirophrenia | PSY | SMI | Read | 450785011 |
| Eu302 | [X]Mania with psychotic symptoms | BPD | SMI | Read | 401863011 |
| E1154 | Bipolar affect disord, now depressed, severe with psychosis | BPD | SMI | Read | 513721000006114 |
| E13y0 | Psychogenic stupor | PSY | SMI | Read | 294927012 |
| Eu200-1 | [X]Paraphrenic schizophrenia | SCH | SMI | Read | 418261000006112 |
| Eu205-2 | [X]Restzustand schizophrenic | SCH | SMI | Read | 424931000006113 |
| E1116 | Recurrent manic episodes, in full remission | BPD | SMI | Read | 294817019 |
| Eu201 | [X]Hebephrenic schizophrenia | SCH | SMI | Read | 388741000006110 |
| E107-1 | Cyclic schizophrenia | PSY | SMI | Read | 294773015 |
| E11zz | Other affective psychosis NOS | BPD | SMI | Read | 294902017 |
| ^ESCTIN350472 | Induced psychotic disorder | PSY | SMI | EMIS | 3504721000006117 |
| Eu21-1 | [X]Latent schizophrenic reaction | PSY | SMI | Read | 395031000006114 |
| Eu319 | [X]Bipolar affective disorder type II | BPD | SMI | Read | 1785861000006112 |
| Eu0z-2 | [X]Symptomatic psychosis NOS | PSY | SMI | Read | 428451000006119 |
| E1145 | Bipolar affect disord,currently manic, part/unspec remission | BPD | SMI | Read | 513741000006119 |
| E1051 | Subchronic latent schizophrenia | PSY | SMI | Read | 294766013 |
| ^ESCTSU272627 | Subchronic disorganized schizophrenia with acute exacerbations | SCH | SMI | EMIS | 2726271000006118 |
| ^ESCTPA352741 | Paranoid schizophrenia, in remission | SCH | SMI | EMIS | 3527411000006119 |
| Eu21-2 | [X]Borderline schizophrenia | SCH | SMI | Read | 367951000006113 |
| ^ESCTDI300559 | Disorganised schizophrenia in remission | SCH | SMI | EMIS | 3005591000006114 |
| ^ESCTSU476373 | Subchronic catatonic schizophrenia with acute exacerbation | SCH | SMI | EMIS | 4763731000006117 |
| ^ESCTLA476386 | Latent schizophrenia, in remission | SCH | SMI | EMIS | 4763861000006116 |
| Eu20y-2 | [X]Schizophreniform disord NOS | SCH | SMI | Read | 425681000006117 |
| E13-1 | Reactive psychoses | PSY | SMI | Read | 346896012 |
| ^ESCTBI276168 | Bipolar I disorder, most recent episode mixed | BPD | SMI | EMIS | 2761681000006119 |
| E121 | Chronic paranoid psychosis | PSY | SMI | Read | 401768012 |
| Eu23z | [X]Acute and transient psychotic disorder, unspecified | PSY | SMI | Read | 401860014 |
| Eu22y-1 | [X]Delusional dysmorphophobia | PSY | SMI | Read | 376501000006110 |
| ^ESCTRE502339 | Reactive psychosis | PSY | SMI | EMIS | 5023391000006116 |
| EMISICD10\|F2035 | Undifferentiated schizophrenia, complete remission | SCH | SMI | EMIS | 1975081000006110 |
| ^ESCTBI300667 | Bipolar I disorder, most recent episode hypomanic | BPD | SMI | EMIS | 3006671000006118 |
| Eu26 | [X]Nonorganic psychosis in remission | PSY | SMI | Read | 1667591000000111 |
| EMISQHY1 | Hypomanic | BPD | SMI | EMIS | 851691000006114 |
| Eu202-4 | [X]Schizophrenic flexibilatis cerea | SCH | SMI | Read | 294743012 |
| E123 | Shared paranoid disorder | PSY | SMI | Read | 294909014 |
| Eu528 | [X]Erotomania | PSY | SMI | Read | 2532965018 |
| Eu203-1 | [X]Atypical schizophrenia | SCH | SMI | Read | 366571000006114 |
| Eu2y-1 | [X]Chronic hallucinatory psychosis | PSY | SMI | Read | 370981000006112 |
| E103 | Paranoid schizophrenia | SCH | SMI | Read | 107878010 |
| E1141 | Bipolar affective disorder, currently manic, mild | BPD | SMI | Read | 294849019 |
| E1033 | Acute exacerbation of subchronic paranoid schizophrenia | SCH | SMI | Read | 294757014 |
| E1114 | Recurrent manic episodes, severe, with psychosis | BPD | SMI | Read | 294815010 |
| ^ESCTCH476379 | Chronic paranoid schizophrenia with acute exacerbation | SCH | SMI | EMIS | 4763791000006118 |
| Eu21-5 | [X]Prodromal schizophrenia | PSY | SMI | Read | 423471000006117 |
| Eu302-1 | [X]Mania with mood-congruent psychotic symptoms | BPD | SMI | Read | 396701000006111 |
| E1053 | Acute exacerbation of subchronic latent schizophrenia | PSY | SMI | Read | 294768014 |
| Eu2 | [X]Schizophrenia, schizotypal and delusional disorders | PSY | SMI | Read | 296022017 |
| E10y1 | Coenesthopathic schizophrenia | SCH | SMI | Read | 294787015 |
| EMISICD10\|F2041 | Post-schizophrenic depression, episodic with progressive deficit | SCH | SMI | EMIS | 1975211000006110 |
| E110-1 | Hypomanic psychoses | BPD | SMI | Read | 789221000006116 |
| Eu220-2 | [X]Paranoid state | PSY | SMI | Read | 418231000006115 |
| Eu204 | [X]Post-schizophrenic depression | SCH | SMI | Read | 423041000006119 |
| E1075 | Schizo-affective schizophrenia in remission | SCH | SMI | Read | 155151000006119 |
| E115 | Bipolar affective disorder, currently depressed | BPD | SMI | Read | 513751000006117 |
| E1035 | Paranoid schizophrenia in remission | SCH | SMI | Read | 105029017 |
| E1111 | Recurrent manic episodes, mild | BPD | SMI | Read | 294812013 |
| E1112 | Recurrent manic episodes, moderate | BPD | SMI | Read | 294813015 |
| E13yz | Other reactive psychoses NOS | PSY | SMI | Read | 294929010 |
| ^ESCTCH386266 | Chronic schizophrenia | SCH | SMI | EMIS | 3862661000006112 |
| Eu232-3 | [X]Oneirophrenia | PSY | SMI | Read | 403611000006116 |
| E1174 | Unspecified bipolar affective disorder,severe with psychosis | BPD | SMI | Read | 82171000006116 |
| E10-98 | Schizophrenic psychoses NOS | SCH | SMI | Read | 882281000006113 |
| Eu232-1 | [X]Brief schizophreniform disorder | PSY | SMI | Read | 368001000006112 |
| 1464 | H/O: schizophrenia | SCH | SMI | Read | 251628010 |
| Eu333-2 | [X]Manic-depress psychosis,depressed type+psychotic symptoms | BPD | SMI | Read | 396781000006119 |
| Eu230-2 | [X]Cycloid psychosis | PSY | SMI | Read | 376271000006119 |
| E1010 | Unspecified hebephrenic schizophrenia | SCH | SMI | Read | 294735019 |
| E1071 | Subchronic schizo-affective schizophrenia | SCH | SMI | Read | 123611000006110 |
| Eu220 | [X]Delusional disorder | PSY | SMI | Read | 215841000000114 |
| E103z | Paranoid schizophrenia NOS | SCH | SMI | Read | 294760019 |
| E1003 | Acute exacerbation of subchronic schizophrenia | SCH | SMI | Read | 294730012 |
| E1020 | Unspecified catatonic schizophrenia | SCH | SMI | Read | 294744018 |
| E1163 | Mixed bipolar affective disorder, severe, without psychosis | BPD | SMI | Read | 701071000006111 |
| EMISICD10\|F2065 | Simple schizophrenia, complete remission | SCH | SMI | EMIS | 1975601000006110 |
| E117z | Unspecified bipolar affective disorder, NOS | BPD | SMI | Read | 294888017 |
| Eu203 | [X]Undifferentiated schizophrenia | SCH | SMI | Read | 296031017 |
| E1011 | Subchronic hebephrenic schizophrenia | SCH | SMI | Read | 294736018 |
| E11-3 | Manic psychoses | PSY | SMI | Read | 223601000000119 |
| Eu30-1 | [X]Bipolar disorder, single manic episode | BPD | SMI | Read | 367151000006111 |
| E11-1 | Bipolar psychoses | BPD | SMI | Read | 513871000006118 |
| E1105 | Single manic episode in partial or unspecified remission | BPD | SMI | Read | 294807012 |
| E1152 | Bipolar affective disorder, currently depressed, moderate | BPD | SMI | Read | 294863013 |
| ^ESCTBI317407 | Bipolar disorder, full remission | BPD | SMI | EMIS | 3174071000006116 |
| ^ESCT1248045 | [X]Schizophrenia, schizotypal and delusional disorders | SCH | SMI | EMIS | 12480451000006112 |
| Eu20y | [X]Other schizophrenia | SCH | SMI | Read | 401855010 |
| E1173 | Unspecified bipolar affective disorder, severe, no psychosis | BPD | SMI | Read | 82151000006114 |
| 4926007 | Schizophrenia in remission | SCH | SMI | SNOMED | 9225016 |
| 31658008 | Chronic paranoid schizophrenia | SCH | SMI | SNOMED | 52897013 |
| 68995007 | Chronic catatonic schizophrenia | SCH | SMI | SNOMED | 114616017 |
| 16990005 | Subchronic schizophrenia | SCH | SMI | SNOMED | 28758018 |
| 42868002 | Subchronic catatonic schizophrenia | SCH | SMI | SNOMED | 71539017 |
| 5464005 | Brief reactive psychosis | PSY | SMI | SNOMED | 10122017 |
| 79866005 | Subchronic paranoid schizophrenia | SCH | SMI | SNOMED | 132503015 |
| 14291003 | Acute exacerbation of subchronic hebephrenic schizophrenia | SCH | SMI | SNOMED | 294738017 |
| 191590005 | Recurrent manic episodes | BPD | SMI | SNOMED | 294810017 |
| 191627008 | Bipolar affective disorder, currently depressed, NOS | BPD | SMI | SNOMED | 294868016 |
| 191668004 | Simple paranoid state | PSY | SMI | SNOMED | 294904016 |
| 231437006 | Other reactive psychoses | PSY | SMI | SNOMED | 294926015 |
| 191539009 | Acute exacerbation of chronic hebephrenic schizophrenia | SCH | SMI | SNOMED | 294739013 |
| 191542003 | Catatonic schizophrenia | SCH | SMI | SNOMED | 294742019 |
| 191547009 | Acute exacerbation of subchronic catatonic schizophrenia | SCH | SMI | SNOMED | 294750011 |
| 58214004 | Other schizophrenia NOS | SCH | SMI | SNOMED | 294789017 |
| 191618007 | Bipolar affective disorder, currently manic, NOS | BPD | SMI | SNOMED | 294858014 |
| 191636007 | Mixed bipolar affective disorder | BPD | SMI | SNOMED | 294869012 |
| 191670008 | Folie a deux | PSY | SMI | SNOMED | 294908018 |
| 191527001 | Simple schizophrenia NOS | SCH | SMI | SNOMED | 294734015 |
| 191588009 | Single manic episode in full remission | BPD | SMI | SNOMED | 294808019 |
| 191667009 | Paranoid psychosis NOS | PSY | SMI | SNOMED | 294913019 |
| 191680007 | Psychogenic paranoid psychosis | PSY | SMI | SNOMED | 294924017 |
| 191527001 | Schizophrenia simplex | SCH | SMI | SNOMED | 294726014 |
| 764641000000104 | Single manic episode, severe without mention of psychosis | BPD | SMI | SNOMED | 294805016 |
| 191586008 | Single manic episode, severe, with psychosis | BPD | SMI | SNOMED | 294806015 |
| 191621009 | Bipolar affective disorder, currently manic, moderate | BPD | SMI | SNOMED | 294850019 |
| 191565008 | Latent schizophrenia in remission | PSY | SMI | SNOMED | 294770017 |
| 191638008 | Mixed bipolar affective disorder, mild | BPD | SMI | SNOMED | 294871012 |
| 35252006 | Hebephrenic schizophrenia NOS | SCH | SMI | SNOMED | 294741014 |
| 191548004 | Acute exacerbation of chronic catatonic schizophrenia | SCH | SMI | SNOMED | 294751010 |
| 191584006 | Single manic episode, moderate | BPD | SMI | SNOMED | 294804017 |
| 191667009 | Other paranoid states | PSY | SMI | SNOMED | 294910016 |
| 268619003 | Single manic episode, unspecified | BPD | SMI | SNOMED | 294802018 |
| 191636007 | Mixed bipolar affective disorder, unspecified | BPD | SMI | SNOMED | 294870013 |
| 191639000 | Mixed bipolar affective disorder, moderate | BPD | SMI | SNOMED | 294872017 |
| 231489001 | [X]Other acute and transient psychotic disorders | PSY | SMI | SNOMED | 296083012 |
| 191618007 | Manic-depressive - now manic | BPD | SMI | SNOMED | 294847017 |
| 191618007 | Bipolar affective disorder, currently manic, unspecified | BPD | SMI | SNOMED | 294848010 |
| 13746004 | Other and unspecified manic-depressive psychoses NOS | BPD | SMI | SNOMED | 294896010 |
| 231487004 | [X]Persistent delusional disorder, unspecified | PSY | SMI | SNOMED | 296066015 |
| 191577003 | Cenesthopathic schizophrenia | SCH | SMI | SNOMED | 294788013 |
| 79584002 | Unspecified bipolar affective disorder, moderate | BPD | SMI | SNOMED | 294883014 |
| 191590005 | Recurrent manic episodes, unspecified | BPD | SMI | SNOMED | 294811018 |
| 191629006 | Bipolar affective disorder, currently depressed, mild | BPD | SMI | SNOMED | 294862015 |
| 16506000 | Other mixed manic-depressive psychoses | BPD | SMI | SNOMED | 294895014 |
| 58214004 | [X]Schizophrenia, unspecified | SCH | SMI | SNOMED | 296040018 |
| 191564007 | Acute exacerbation of chronic latent schizophrenia | PSY | SMI | SNOMED | 294769018 |
| 191562006 | Chronic latent schizophrenia | PSY | SMI | SNOMED | 294767016 |
| 41836007 | Unspecified bipolar affective disorder, in full remission | BPD | SMI | SNOMED | 294887010 |
| 191658009 | Atypical manic disorder | BPD | SMI | SNOMED | 294893019 |
| 68890003 | [X]Schizoaffective disorder, unspecified | PSY | SMI | SNOMED | 296096013 |
| 191542003 | Catatonic schizophrenia NOS | SCH | SMI | SNOMED | 294753013 |
| 191559008 | Latent schizophrenia | PSY | SMI | SNOMED | 294764011 |
| 191643001 | Mixed bipolar affective disorder, in full remission | BPD | SMI | SNOMED | 294876019 |
| 13746004 | Unspecified bipolar affective disorder | BPD | SMI | SNOMED | 294880012 |
| 13313007 | Unspecified bipolar affective disorder, mild | BPD | SMI | SNOMED | 294882016 |
| 441704009 | Unspecified affective psychoses NOS | BPD | SMI | SNOMED | 294898011 |
| 191526005 | Schizophrenic disorders | SCH | SMI | SNOMED | 294725013 |
| 191527001 | Simple schizophrenia | SCH | SMI | SNOMED | 294727017 |
| 12939007 | Chronic hebephrenic schizophrenia | SCH | SMI | SNOMED | 294737010 |
| 191636007 | Mixed bipolar affective disorder, NOS | BPD | SMI | SNOMED | 294877011 |
| 441704009 | Other and unspecified affective psychoses | BPD | SMI | SNOMED | 294897018 |
| 13746004 | [X]Bipolar affective disorder, unspecified | BPD | SMI | SNOMED | 296135011 |
| 58214004 | Unspecified schizophrenia | SCH | SMI | SNOMED | 294728010 |
| 31373002 | Hebephrenic schizophrenia in remission | SCH | SMI | SNOMED | 294740010 |
| 58214004 | Schizophrenia NOS | SCH | SMI | SNOMED | 294790014 |
| 13746004 | Unspecified bipolar affective disorder, unspecified | BPD | SMI | SNOMED | 294881011 |
| 191677006 | Acute hysterical psychosis | PSY | SMI | SNOMED | 294919015 |
| 191559008 | Latent schizophrenia NOS | PSY | SMI | SNOMED | 294771018 |
| 268622001 | Sanders disease | PSY | SMI | SNOMED | 161371000006118 |
| 231489001 | [X]Other acute predominantly delusional psychotic disorders | PSY | SMI | SNOMED | 401859016 |
| 13746004 | [X]Other bipolar affective disorders | BPD | SMI | SNOMED | 401865016 |
| 764671000000105 | Recurrent manic episodes, partial or unspecified remission | BPD | SMI | SNOMED | 182861000006110 |
| 268619003 | [X]Manic episode, unspecified | BPD | SMI | SNOMED | 401864017 |
| 417233008 | [X]Paranoia | PSY | SMI | SNOMED | 215871000000115 |
| 191567000 | Schizo-affective schizophrenia NOS | SCH | SMI | SNOMED | 155161000006117 |
| 191667009 | [X]Paranoid psychosis | PSY | SMI | SNOMED | 215851000000112 |
| 12200008 | Poor insight into psychotic condition | PSY | SMI | SNOMED | 215691000006112 |
| 31027006 | [X]Schizotypal disorder | PSY | SMI | SNOMED | 401856011 |
| 268619003 | Manic disorder, single episode | BPD | SMI | SNOMED | 401765010 |
| 764621000000106 | Recurrent manic episodes, severe without mention psychosis | BPD | SMI | SNOMED | 182871000006115 |
| 58214004 | Other schizophrenia | SCH | SMI | SNOMED | 401764014 |
| 191525009 | Non-organic psychosis NOS | PSY | SMI | SNOMED | 401771016 |
| 161464003 | [V]Personal history of schizophrenia | SCH | SMI | SNOMED | 460273017 |
| 278853003 | [X]Acute schizophrenia-like psychotic disorder | PSY | SMI | SNOMED | 362421000006113 |
| 191525009 | [X]Unspecified nonorganic psychosis | PSY | SMI | SNOMED | 401862018 |
| 191667009 | Paranoid states | PSY | SMI | SNOMED | 243361000006117 |
| 268617001 | Acute schizophrenic episode | SCH | SMI | SNOMED | 401763015 |
| 5703000 | Unspecified bipolar affect disord, partial/unspec remission | BPD | SMI | SNOMED | 82091000006116 |
| 429124005 | [V]Personal history of manic-depressive psychosis | BPD | SMI | SNOMED | 1227584015 |
| 63204009 | [X]Bouffee delirante | PSY | SMI | SNOMED | 367961000006110 |
| 712850003 | [X]Bouffee delirante with symptoms of schizophrenia | PSY | SMI | SNOMED | 367971000006115 |
| 13746004 | [X]Bipolar affective disorder | BPD | SMI | SNOMED | 367101000006112 |
| 192362008 | [X]Bipolar affective disorder, current episode mixed | BPD | SMI | SNOMED | 367121000006119 |
| 191623007 | [X]Bipolar affect disorder cur epi manic with psychotic symp | BPD | SMI | SNOMED | 367071000006119 |
| 191542003 | [X]Catatonic schizophrenia | SCH | SMI | SNOMED | 370451000006110 |
| 191559008 | [X]Latent schizophrenia | PSY | SMI | SNOMED | 395021000006111 |
| 191618007 | Bipolar affective disorder, currently manic | BPD | SMI | SNOMED | 513801000006112 |
| 191670008 | [X]Folie a deux | PSY | SMI | SNOMED | 387071000006117 |
| 49512000 | Bipolar affect disord, now depressed, part/unspec remission | BPD | SMI | SNOMED | 513711000006118 |
| 63204009 | Bouffee delirante | PSY | SMI | SNOMED | 523481000006114 |
| 83225003 | [X]Bipolar II disorder | BPD | SMI | SNOMED | 367161000006113 |
| 58214004 | [X]Cenesthopathic schizophrenia | SCH | SMI | SNOMED | 370631000006117 |
| 191623007 | Bipolar affect disord, currently manic,severe with psychosis | BPD | SMI | SNOMED | 513701000006116 |
| 405273008 | Manic mood | BPD | SMI | SNOMED | 2157096015 |
| 191570001 | Chronic schizo-affective schizophrenia | SCH | SMI | SNOMED | 556631000006116 |
| 191567000 | Unspecified schizo-affective schizophrenia | SCH | SMI | SNOMED | 78531000006116 |
| 765176007 | [X]Bipolar affect dis cur epi severe depres with psyc symp | BPD | SMI | SNOMED | 367061000006114 |
| 38295006 | [X]Paraphrenia - late | PSY | SMI | SNOMED | 418251000006110 |
| 191590005 | [X]Recurrent manic episodes | BPD | SMI | SNOMED | 424661000006111 |
| 191542003 | [X]Schizophrenic catalepsy | SCH | SMI | SNOMED | 425641000006111 |
| 58214004 | [X]Schizophrenifrm psychos NOS | SCH | SMI | SNOMED | 425711000006116 |
| 231496004 | [X]Hypomania | BPD | SMI | SNOMED | 389401000006111 |
| 271428004 | [X]Schizoaffective psychosis, manic type | PSY | SMI | SNOMED | 425571000006119 |
| 31027006 | [X]Pseudopsychopathic schizophrenia | PSY | SMI | SNOMED | 423741000006116 |
| 84760002 | [X]Schizophreniform psychosis, depressive type | PSY | SMI | SNOMED | 425691000006119 |
| 26025008 | [X]Residual schizophrenia | SCH | SMI | SNOMED | 424841000006113 |
| 191542003 | [X]Schizophrenic catatonia | SCH | SMI | SNOMED | 425651000006113 |
| 231487004 | [X]Persistent delusional disorders | PSY | SMI | SNOMED | 419861000006117 |
| 68890003 | [X]Schizoaffective psychosis NOS | PSY | SMI | SNOMED | 425551000006112 |
| 84760002 | [X]Schizoaffective psychosis, depressive type | PSY | SMI | SNOMED | 425561000006114 |
| 191680007 | [X]Psychogenic paranoid psychosis | PSY | SMI | SNOMED | 424071000006112 |
| 191525009 | Nonorganic psychosis NOS | PSY | SMI | SNOMED | 223611000000117 |
| 64905009 | [X]Paranoid schizophrenia | SCH | SMI | SNOMED | 418221000006118 |
| 268619003 | [X]Manic episode | BPD | SMI | SNOMED | 396741000006113 |
| 192362008 | [X]Mixed affective episode | BPD | SMI | SNOMED | 398541000006116 |
| 268619003 | [X]Mania NOS | BPD | SMI | SNOMED | 396691000006111 |
| 35252006 | Hebephrenic schizophrenia | SCH | SMI | SNOMED | 819351000006115 |
| 31027006 | [X]Pseudoneurotic schizophrenia | PSY | SMI | SNOMED | 423731000006114 |
| 278853003 | [X]Schizophrenic reaction | SCH | SMI | SNOMED | 425671000006115 |
| 41189006 | [X]Sensitiver Beziehungswahn | PSY | SMI | SNOMED | 425921000006114 |
| 191618007 | [X]Bipolar affect disorder cur epi manic wout psychotic symp | BPD | SMI | SNOMED | 367081000006116 |
| 191567000 | Acute schizo affective psychosis | PSY | SMI | SNOMED | 882301000006112 |
| 61831009 | [X]Induced delusional disorder | PSY | SMI | SNOMED | 389641000006110 |
| 13746004 | Manic-depressive psychoses | BPD | SMI | SNOMED | 882311000006110 |
| 61831009 | [X]Induced psychotic disorder | PSY | SMI | SNOMED | 389661000006114 |
| 46206005 | Affective psychoses | BPD | SMI | SNOMED | 473201000006114 |
| 429124005 | [V]Personal history of manic-depressive psychosis | BPD | SMI | SNOMED | 345141000006111 |
| 31446002 | [X]Bipolar affective disorder, current episode hypomanic | BPD | SMI | SNOMED | 367111000006110 |
| 712850003 | [X]Acute polymorphic psychot disord with symp of schizophren | PSY | SMI | SNOMED | 362381000006115 |
| 712824002 | [X]Acute polymorphic psychot disord without symp of schizoph | PSY | SMI | SNOMED | 362391000006117 |
| 44906001 | [X]Delusional misidentification syndrome | PSY | SMI | SNOMED | 914471000006113 |
| 36474008 | [X]Manic-depress psychosis,depressd,no psychotic symptoms | BPD | SMI | SNOMED | 396771000006117 |
| 231494001 | [X]Manic stupor | BPD | SMI | SNOMED | 396761000006112 |
| 191625000 | Bipolar affective disorder, currently manic, full remission | BPD | SMI | SNOMED | 513811000006110 |
| 58214004 | [X]Schizophrenia | SCH | SMI | SNOMED | 425601000006114 |
| 31027006 | [X]Schizotypal personality disorder | PSY | SMI | SNOMED | 425731000006110 |
| 83225003 | [X]Bipolar II disorder | BPD | SMI | SNOMED | 1785871000006117 |
| 271428004 | [X]Schizoaffective disorder, manic type | PSY | SMI | SNOMED | 425511000006111 |
| 755301000000102 | [X]Paranoid state in remission | PSY | SMI | SNOMED | 1667581000000114 |
| 231437006 | [X]Reactive psychosis | PSY | SMI | SNOMED | 424511000006112 |
| 69322001 | [X]Psychosis NOS | PSY | SMI | SNOMED | 424231000006116 |
| 271428004 | [X]Schizophreniform psychosis, manic type | PSY | SMI | SNOMED | 425701000006119 |
| 371596008 | [X]Bipolar affective disorder type I | BPD | SMI | SNOMED | 1785851000006110 |
| 191572009 | Acute exacerbation of chronic schizo-affective schizophrenia | SCH | SMI | SNOMED | 456731000006115 |
| 1976961000006106 | Schizophrenia, unspecified, other | SCH | SMI | SNOMED | 1976961000006110 |
| 760721000000109 | Mixed bipolar affective disorder, partial/unspec remission | BPD | SMI | SNOMED | 701051000006118 |
| 63204009 | BouffÃ©e dÃ©lirante | PSY | SMI | SNOMED | 3527771000006116 |
| 5464005 | Brief psychotic disorder | PSY | SMI | SNOMED | 2586801000006115 |
| 1976891000006106 | Hebephrenic schizophrenia, other | SCH | SMI | SNOMED | 1976891000006110 |
| 14291003 | Subchronic disorganised schizophrenia with acute exacerbations | SCH | SMI | SNOMED | 2726261000006113 |
| 61831009 | Folie E deux | PSY | SMI | SNOMED | 3504781000006118 |
| 61831009 | Induced psychosis | PSY | SMI | SNOMED | 3504751000006114 |
| 31373002 | Disorganized schizophrenia in remission | SCH | SMI | SNOMED | 3005601000006118 |
| 61831009 | Shared psychotic disorder | PSY | SMI | SNOMED | 3504741000006112 |
| 61403008 | Severe bipolar I disorder, most recent episode depressed without psychotic features | BPD | SMI | SNOMED | 3497641000006117 |
| 27387000 | Subchronic disorganized schizophrenia | SCH | SMI | SNOMED | 2938961000006110 |
| 981141000006107 | Delusions | PSY | SMI | SNOMED | 981141000006111 |
| 192362008 | Bipolar affective disorder, current episode mixed | BPD | SMI | SNOMED | 4767311000006117 |
| 35252006 | Disorganised schizophrenia | SCH | SMI | SNOMED | 3068121000006112 |
| 191526005 | Schizophrenic psychoses | SCH | SMI | SNOMED | 882291000006111 |
| 247804008 | Schizophrenic prodrome | SCH | SMI | SNOMED | 5248961000006113 |
| 31373002 | Disorganized schizophrenia, in remission | SCH | SMI | SNOMED | 3005611000006115 |
| 38295006 | Involutional paraphrenia | PSY | SMI | SNOMED | 3116711000006110 |
| 280949006 | De Clerambaults syndrome | PSY | SMI | SNOMED | 5645621000006116 |
| 191571002 | Acute exacerbation of subchronic schizoaffective schizophrenia | SCH | SMI | SNOMED | 4763911000006114 |
| 191548004 | Chronic catatonic schizophrenia with acute exacerbation | SCH | SMI | SNOMED | 4763751000006112 |
| 191542003 | Schizophrenia, catatonic | SCH | SMI | SNOMED | 4763691000006113 |
| 755311000000100 | Non-organic psychosis in remission | PSY | SMI | SNOMED | 8232541000006114 |
| 12939007 | Chronic disorganised schizophrenia | SCH | SMI | SNOMED | 2705561000006112 |
| 12939007 | Chronic disorganized schizophrenia | SCH | SMI | SNOMED | 2705571000006117 |
| 162004 | Severe bipolar I disorder, most recent episode manic, without psychotic features | BPD | SMI | SNOMED | 2501011000006119 |
| 760721000000109 | Mixed bipolar affective disorder, in partial remission | BPD | SMI | SNOMED | 8235161000006110 |
| 4926007 | Schizophrenia, in remission | SCH | SMI | SNOMED | 2577941000006118 |
| 61831009 | Induced paranoid disorder | PSY | SMI | SNOMED | 3504791000006115 |
| 191569002 | Subchronic schizoaffective schizophrenia | SCH | SMI | SNOMED | 4763891000006112 |
| 27387000 | Subchronic disorganised schizophrenia | SCH | SMI | SNOMED | 2938951000006113 |
| 191539009 | Acute exacerbation of chronic disorganised schizophrenia | SCH | SMI | SNOMED | 4763641000006116 |
| 84760002 | Schizophreniform psychosis, depressive type | SCH | SMI | SNOMED | 3878611000006116 |
| 111482003 | Subchronic schizophrenia with acute exacerbations | SCH | SMI | SNOMED | 4196151000006111 |
| 270901009 | Mixed schizophrenic and affective psychosis | PSY | SMI | SNOMED | 5517971000006118 |
| 271428004 | Schizophreniform psychosis, manic type | PSY | SMI | SNOMED | 5524571000006112 |
| 191542003 | Schizophrenic flexibilatis cerea | SCH | SMI | SNOMED | 4763701000006113 |
| 111483008 | Schizophrenia, catatonic, in remission | SCH | SMI | SNOMED | 4196171000006118 |
| 85248005 | Bipolar disorder, in remission | BPD | SMI | SNOMED | 3886291000006119 |
| 69322001 | Psychosis | PSY | SMI | SNOMED | 3627341000006115 |
| 371596008 | Bipolar 1 disorder | BPD | SMI | SNOMED | 6357571000006119 |
| 13746004 | MDI - Manic-depressive illness | BPD | SMI | SNOMED | 2717981000006118 |
| 26025008 | Restzustand | SCH | SMI | SNOMED | 2916361000006118 |
| 31027006 | Schizoptypal disorder | SCH | SMI | SNOMED | 3000261000006112 |
| 35252006 | Disorganized schizophrenia | SCH | SMI | SNOMED | 3068131000006110 |
| 191539009 | Acute exacerbation of chronic disorganized schizophrenia | SCH | SMI | SNOMED | 4763661000006117 |
| 63249007 | Bipolar I disorder, most recent episode manic, in partial remission | BPD | SMI | SNOMED | 3528681000006111 |
| 198991000000103 | H/O: psychosis | PSY | SMI | SNOMED | 300411000000110 |
| 231489001 | [X]Brief reactive psychosis NOS | PSY | SMI | SNOMED | 367991000006119 |
| 162004 | Bipolar affect disord, currently manic, severe, no psychosis | BPD | SMI | SNOMED | 513691000006116 |
| 270901009 | [X]Schizoaffective disorder, mixed type | PSY | SMI | SNOMED | 425521000006115 |
| 231487004 | [X]Other persistent delusional disorders | PSY | SMI | SNOMED | 401857019 |
| 231489001 | [X]Acute and transient psychotic disorders | PSY | SMI | SNOMED | 362271000006110 |
| 191634005 | Bipolar affective disorder, now depressed, in full remission | BPD | SMI | SNOMED | 513861000006113 |
| 83746006 | Chronic schizophrenic | SCH | SMI | SNOMED | 1234861017 |
| 68890003 | [X]Schizoaffective disorders | PSY | SMI | SNOMED | 425541000006110 |
| 231487004 | [X]Paranoia querulans | PSY | SMI | SNOMED | 418181000006110 |
| 191571002 | Acute exacerbation subchronic schizo-affective schizophrenia | SCH | SMI | SNOMED | 456801000006115 |
| 111483008 | Catatonic schizophrenia in remission | SCH | SMI | SNOMED | 178723016 |
| 64905009 | Unspecified paranoid schizophrenia | SCH | SMI | SNOMED | 294754019 |
| 191525009 | Non-organic psychoses | PSY | SMI | SNOMED | 294724012 |
| 851701000006105 | Paranoid | PSY | SMI | SNOMED | 851701000006114 |
| 268624000 | Acute paranoid reaction | PSY | SMI | SNOMED | 401770015 |
| 191590005 | Recurrent manic episode NOS | BPD | SMI | SNOMED | 294818012 |
| 68890003 | [X]Other schizoaffective disorders | PSY | SMI | SNOMED | 296095012 |
| 307417003 | [X]Cycloid psychosis with symptoms of schizophrenia | PSY | SMI | SNOMED | 376281000006116 |
| 231487004 | [X]Involutional paranoid state | PSY | SMI | SNOMED | 394461000006110 |
| 191667009 | Other paranoid states NOS | PSY | SMI | SNOMED | 294912012 |
| 853201000006100 | Obsessional compulsive psychosis | PSY | SMI | SNOMED | 853201000006116 |
| 191525009 | Other specified non-organic psychoses | PSY | SMI | SNOMED | 294949018 |
| 231494001 | [X]Mania without psychotic symptoms | BPD | SMI | SNOMED | 296110017 |
| 270901009 | [X]Cyclic schizophrenia | PSY | SMI | SNOMED | 376251000006112 |
| 13746004 | Unspecified manic-depressive psychoses | BPD | SMI | SNOMED | 294892012 |
| 26025008 | Residual schizophrenia | SCH | SMI | SNOMED | 43595011 |
| 231494001 | [X]Other manic episodes | BPD | SMI | SNOMED | 296118012 |
| 83225003 | Bipolar 2 disorder | BPD | SMI | SNOMED | 3854401000006113 |
| 191641004 | Mixed bipolar affective disorder, severe, with psychosis | BPD | SMI | SNOMED | 294874016 |
| 61831009 | Symbiotic psychosis | PSY | SMI | SNOMED | 3504761000006111 |
| 1975441000006106 | Residual schizophrenia, incomplete remission | SCH | SMI | SNOMED | 1975441000006110 |
| 13746004 | [X]Manic-depressive psychosis | BPD | SMI | SNOMED | 396801000006115 |
| 712850003 | Acute polymorphic psychotic disorder with symptoms of schizophrenia | PSY | SMI | SNOMED | 7696261000006113 |
| 13746004 | [X]Manic-depressive illness | BPD | SMI | SNOMED | 396791000006116 |
| 191527001 | [X]Simple schizophrenia | SCH | SMI | SNOMED | 426881000006111 |
| 191630001 | [X]Bipolar affect disorder cur epi mild or moderate depressn | BPD | SMI | SNOMED | 367091000006118 |
| 44906001 | [X]Capgras syndrome | PSY | SMI | SNOMED | 914461000006118 |
| 46206005 | [X]Affective psychosis NOS | BPD | SMI | SNOMED | 362781000006116 |
| 191574005 | Schizophrenia, schizoaffective, in remission | SCH | SMI | SNOMED | 4763951000006110 |
| 231494001 | [X]Mania with mood-incongruent psychotic symptoms | BPD | SMI | SNOMED | 396711000006114 |
| 61403008 | Bipolar affect disord, now depressed, severe, no psychosis | BPD | SMI | SNOMED | 513731000006112 |
| 64905009 | Paraphrenic schizophrenia | SCH | SMI | SNOMED | 3555731000006114 |
| 400998002 | H/O: manic depressive disorder | BPD | SMI | SNOMED | 1780205015 |
| 191567000 | Schizo-affective schizophrenia | SCH | SMI | SNOMED | 155141000006116 |
| 270901009 | [X]Mixed schizophrenic and affective psychosis | PSY | SMI | SNOMED | 398631000006113 |
| 61831009 | [X]Induced paranoid disorder | PSY | SMI | SNOMED | 389651000006112 |
| 247804008 | [X]Prepsychotic schizophrenia | PSY | SMI | SNOMED | 423271000006116 |
| 31027006 | Schizotypal personality | PSY | SMI | SNOMED | 155281000006119 |
| 26472000 | Paraphrenia | PSY | SMI | SNOMED | 44335019 |
| 191525009 | [X]Other nonorganic psychotic disorders | PSY | SMI | SNOMED | 412201000006113 |
| 111484002 | Atypical schizophrenia | SCH | SMI | SNOMED | 1219653018 |
| 35252006 | [X]Disorganised schizophrenia | SCH | SMI | SNOMED | 378051000006119 |
| 231485007 | Post-schizophrenic depression | SCH | SMI | SNOMED | 5023901000006110 |
| 191542003 | [X]Catatonic stupor | SCH | SMI | SNOMED | 370461000006112 |
| 191627008 | Manic-depressive - now depressed | BPD | SMI | SNOMED | 294860011 |
| 191672000 | Paranoia querulans | PSY | SMI | SNOMED | 294911017 |
| 69322001 | Psychotic episode NOS | PSY | SMI | SNOMED | 346895011 |
| 268619003 | Mania/hypomania | BPD | SMI | SNOMED | 882321000006119 |
| 191583000 | Single manic episode, mild | BPD | SMI | SNOMED | 294803011 |
| 191555002 | Acute exacerbation of chronic paranoid schizophrenia | SCH | SMI | SNOMED | 294758016 |
| 111484002 | [X]Chronic undifferentiated schizophrenia | SCH | SMI | SNOMED | 371031000006115 |
| 84760002 | [X]Schizoaffective disorder, depressive type | PSY | SMI | SNOMED | 425501000006113 |
| 26025008 | Restzustand - schizophrenia | SCH | SMI | SNOMED | 169061000006112 |
| 13746004 | [X]Manic-depressive reaction | BPD | SMI | SNOMED | 396071000006119 |
| 191627008 | Bipolar affective disorder, currently depressed, unspecified | BPD | SMI | SNOMED | 294861010 |
| 191559008 | Unspecified latent schizophrenia | PSY | SMI | SNOMED | 294765012 |
| 191574005 | Schizoaffective schizophrenia, in remission | SCH | SMI | SNOMED | 4763941000006113 |
| 85248005 | [X]Bipolar affective disorder, currently in remission | BPD | SMI | SNOMED | 296130018 |
| 61831009 | Folie Ã  deux | PSY | SMI | SNOMED | 3504731000006119 |
| 191554003 | Subchronic paranoid schizophrenia with acute exacerbation | SCH | SMI | SNOMED | 4763771000006119 |
| 61403008 | [X]Bipol aff disord, curr epis sev depress, no psychot symp | BPD | SMI | SNOMED | 367051000006112 |
| 191531007 | Acute exacerbation of chronic schizophrenia | SCH | SMI | SNOMED | 294731011 |
| 268619003 | Manic disorder, single episode NOS | BPD | SMI | SNOMED | 294809010 |
| 191667009 | Paranoia | PSY | SMI | SNOMED | 882331000006116 |
| 278853003 | [X]Brief schizophrenifrm psych | PSY | SMI | SNOMED | 368011000006110 |
| 191525009 | Other nonorganic psychoses | PSY | SMI | SNOMED | 25461000006115 |
| 191572009 | Acute exacerbation of chronic schizoaffective schizophrenia | SCH | SMI | SNOMED | 4763921000006118 |
| 13746004 | Other and unspecified manic-depressive psychoses | BPD | SMI | SNOMED | 294891017 |
| 231494001 | Manic | BPD | SMI | SNOMED | 5023961000006111 |
| 307504004 | Oneirophrenia | PSY | SMI | SNOMED | 450785011 |
| 231494001 | [X]Mania with psychotic symptoms | BPD | SMI | SNOMED | 401863011 |
| 765176007 | Bipolar affect disord, now depressed, severe with psychosis | BPD | SMI | SNOMED | 513721000006114 |
| 191683009 | Psychogenic stupor | PSY | SMI | SNOMED | 294927012 |
| 64905009 | [X]Paraphrenic schizophrenia | SCH | SMI | SNOMED | 418261000006112 |
| 26025008 | [X]Restzustand schizophrenic | SCH | SMI | SNOMED | 424931000006113 |
| 191597008 | Recurrent manic episodes, in full remission | BPD | SMI | SNOMED | 294817019 |
| 35252006 | [X]Hebephrenic schizophrenia | SCH | SMI | SNOMED | 388741000006110 |
| 191567000 | Cyclic schizophrenia | PSY | SMI | SNOMED | 294773015 |
| 441704009 | Other affective psychosis NOS | BPD | SMI | SNOMED | 294902017 |
| 61831009 | Induced psychotic disorder | PSY | SMI | SNOMED | 3504721000006117 |
| 191559008 | [X]Latent schizophrenic reaction | PSY | SMI | SNOMED | 395031000006114 |
| 83225003 | [X]Bipolar affective disorder type II | BPD | SMI | SNOMED | 1785861000006112 |
| 280427006 | [X]Symptomatic psychosis NOS | PSY | SMI | SNOMED | 428451000006119 |
| 63249007 | Bipolar affect disord,currently manic, part/unspec remission | BPD | SMI | SNOMED | 513741000006119 |
| 191561004 | Subchronic latent schizophrenia | PSY | SMI | SNOMED | 294766013 |
| 14291003 | Subchronic disorganized schizophrenia with acute exacerbations | SCH | SMI | SNOMED | 2726271000006118 |
| 63181006 | Paranoid schizophrenia, in remission | SCH | SMI | SNOMED | 3527411000006119 |
| 274952002 | [X]Borderline schizophrenia | SCH | SMI | SNOMED | 367951000006113 |
| 31373002 | Disorganised schizophrenia in remission | SCH | SMI | SNOMED | 3005591000006114 |
| 191547009 | Subchronic catatonic schizophrenia with acute exacerbation | SCH | SMI | SNOMED | 4763731000006117 |
| 191565008 | Latent schizophrenia, in remission | SCH | SMI | SNOMED | 4763861000006116 |
| 58214004 | [X]Schizophreniform disord NOS | SCH | SMI | SNOMED | 425681000006117 |
| 231437006 | Reactive psychoses | PSY | SMI | SNOMED | 346896012 |
| 16506000 | Bipolar I disorder, most recent episode mixed | BPD | SMI | SNOMED | 2761681000006119 |
| 268622001 | Chronic paranoid psychosis | PSY | SMI | SNOMED | 401768012 |
| 231489001 | [X]Acute and transient psychotic disorder, unspecified | PSY | SMI | SNOMED | 401860014 |
| 231487004 | [X]Delusional dysmorphophobia | PSY | SMI | SNOMED | 376501000006110 |
| 231437006 | Reactive psychosis | PSY | SMI | SNOMED | 5023391000006116 |
| 1975081000006106 | Undifferentiated schizophrenia, complete remission | SCH | SMI | SNOMED | 1975081000006110 |
| 31446002 | Bipolar I disorder, most recent episode hypomanic | BPD | SMI | SNOMED | 3006671000006118 |
| 755311000000100 | [X]Nonorganic psychosis in remission | PSY | SMI | SNOMED | 1667591000000111 |
| 851691000006105 | Hypomanic | BPD | SMI | SNOMED | 851691000006114 |
| 191542003 | [X]Schizophrenic flexibilatis cerea | SCH | SMI | SNOMED | 294743012 |
| 191670008 | Shared paranoid disorder | PSY | SMI | SNOMED | 294909014 |
| 280949006 | [X]Erotomania | PSY | SMI | SNOMED | 2532965018 |
| 111484002 | [X]Atypical schizophrenia | SCH | SMI | SNOMED | 366571000006114 |
| 480111000000107 | [X]Chronic hallucinatory psychosis | PSY | SMI | SNOMED | 370981000006112 |
| 64905009 | Paranoid schizophrenia | SCH | SMI | SNOMED | 107878010 |
| 191620005 | Bipolar affective disorder, currently manic, mild | BPD | SMI | SNOMED | 294849019 |
| 191554003 | Acute exacerbation of subchronic paranoid schizophrenia | SCH | SMI | SNOMED | 294757014 |
| 191595000 | Recurrent manic episodes, severe, with psychosis | BPD | SMI | SNOMED | 294815010 |
| 191555002 | Chronic paranoid schizophrenia with acute exacerbation | SCH | SMI | SNOMED | 4763791000006118 |
| 247804008 | [X]Prodromal schizophrenia | PSY | SMI | SNOMED | 423471000006117 |
| 231494001 | [X]Mania with mood-congruent psychotic symptoms | BPD | SMI | SNOMED | 396701000006111 |
| 191563001 | Acute exacerbation of subchronic latent schizophrenia | PSY | SMI | SNOMED | 294768014 |
| 417601000000102 | [X]Schizophrenia, schizotypal and delusional disorders | PSY | SMI | SNOMED | 296022017 |
| 191577003 | Coenesthopathic schizophrenia | SCH | SMI | SNOMED | 294787015 |
| 1975211000006106 | Post-schizophrenic depression, episodic with progressive deficit | SCH | SMI | SNOMED | 1975211000006110 |
| 231496004 | Hypomanic psychoses | BPD | SMI | SNOMED | 789221000006116 |
| 191667009 | [X]Paranoid state | PSY | SMI | SNOMED | 418231000006115 |
| 231485007 | [X]Post-schizophrenic depression | SCH | SMI | SNOMED | 423041000006119 |
| 191574005 | Schizo-affective schizophrenia in remission | SCH | SMI | SNOMED | 155151000006119 |
| 191627008 | Bipolar affective disorder, currently depressed | BPD | SMI | SNOMED | 513751000006117 |
| 63181006 | Paranoid schizophrenia in remission | SCH | SMI | SNOMED | 105029017 |
| 191592002 | Recurrent manic episodes, mild | BPD | SMI | SNOMED | 294812013 |
| 191593007 | Recurrent manic episodes, moderate | BPD | SMI | SNOMED | 294813015 |
| 231437006 | Other reactive psychoses NOS | PSY | SMI | SNOMED | 294929010 |
| 83746006 | Chronic schizophrenia | SCH | SMI | SNOMED | 3862661000006112 |
| 278853003 | [X]Oneirophrenia | PSY | SMI | SNOMED | 403611000006116 |
| 4441000 | Unspecified bipolar affective disorder,severe with psychosis | BPD | SMI | SNOMED | 82171000006116 |
| 191526005 | Schizophrenic psychoses NOS | SCH | SMI | SNOMED | 882281000006113 |
| 278853003 | [X]Brief schizophreniform disorder | PSY | SMI | SNOMED | 368001000006112 |
| 161468000 | H/O: schizophrenia | SCH | SMI | SNOMED | 251628010 |
| 765176007 | [X]Manic-depress psychosis,depressed type+psychotic symptoms | BPD | SMI | SNOMED | 396781000006119 |
| 307417003 | [X]Cycloid psychosis | PSY | SMI | SNOMED | 376271000006119 |
| 35252006 | Unspecified hebephrenic schizophrenia | SCH | SMI | SNOMED | 294735019 |
| 191569002 | Subchronic schizo-affective schizophrenia | SCH | SMI | SNOMED | 123611000006110 |
| 48500005 | [X]Delusional disorder | PSY | SMI | SNOMED | 215841000000114 |
| 64905009 | Paranoid schizophrenia NOS | SCH | SMI | SNOMED | 294760019 |
| 111482003 | Acute exacerbation of subchronic schizophrenia | SCH | SMI | SNOMED | 294730012 |
| 191542003 | Unspecified catatonic schizophrenia | SCH | SMI | SNOMED | 294744018 |
| 764591000000108 | Mixed bipolar affective disorder, severe, without psychosis | BPD | SMI | SNOMED | 701071000006111 |
| 1975601000006106 | Simple schizophrenia, complete remission | SCH | SMI | SNOMED | 1975601000006110 |
| 13746004 | Unspecified bipolar affective disorder, NOS | BPD | SMI | SNOMED | 294888017 |
| 111484002 | [X]Undifferentiated schizophrenia | SCH | SMI | SNOMED | 296031017 |
| 27387000 | Subchronic hebephrenic schizophrenia | SCH | SMI | SNOMED | 294736018 |
| 231494001 | Manic psychoses | PSY | SMI | SNOMED | 223601000000119 |
| 268619003 | [X]Bipolar disorder, single manic episode | BPD | SMI | SNOMED | 367151000006111 |
| 13746004 | Bipolar psychoses | BPD | SMI | SNOMED | 513871000006118 |
| 764731000000103 | Single manic episode in partial or unspecified remission | BPD | SMI | SNOMED | 294807012 |
| 191630001 | Bipolar affective disorder, currently depressed, moderate | BPD | SMI | SNOMED | 294863013 |
| 41836007 | Bipolar disorder, full remission | BPD | SMI | SNOMED | 3174071000006116 |
| 417601000000102 | [X]Schizophrenia, schizotypal and delusional disorders | SCH | SMI | SNOMED | 12480451000006112 |
| 58214004 | [X]Other schizophrenia | SCH | SMI | SNOMED | 401855010 |
| 53049002 | Unspecified bipolar affective disorder, severe, no psychosis | BPD | SMI | SNOMED | 82151000006114 |
